# Transition of children with life-limiting conditions to adult care and healthcare use: a systematic review

**DOI:** 10.1038/s41390-021-01396-8

**Published:** 2021-03-02

**Authors:** Stuart W. Jarvis, Daniel Roberts, Kate Flemming, Gerry Richardson, Lorna K. Fraser

**Affiliations:** 1 Martin House Research Centre, University of York, York, UK; 2 Leeds Children’s Hospital, Leeds, UK; 3 Department of Health Sciences, University of York, York, UK; 4 Centre for Health Economics, University of York, York, UK

## Abstract

**Background:**

Improved survival has led to increasing numbers of children with life-limiting conditions transitioning to adult healthcare services. There are concerns that transition may lead to a reduction in care quality and increases in emergency care. This review explores evidence for differences in health or social care use post- versus pre-transition to adult services.

**Methods:**

MEDLINE, EMBASE, CINAHL, PsychINFO and Social Science Citation Index were searched. Studies published in English since 1990 including individuals with any life-limiting condition post- and pre-transition and reporting a health or social care use outcome were included. Data were extracted and quality assessed by one reviewer with 30% checked by an independent reviewer.

**Results:**

Nineteen papers (18 studies) met the inclusion criteria. There was evidence for both increases and decreases (post- versus pre-transition) in outpatient attendance, inpatient admissions, inpatient bed days and health service costs; for increases in Emergency Department visits and for decreases in individuals receiving physiotherapy.

**Conclusions:**

Evidence for changes in healthcare use post- versus pre-transition is mixed and conflicting, although there is evidence for an increase in Emergency Department visits and a reduction in access to physiotherapy. More high-quality research is needed to better link changes in care to the transition.

**Impact:**

- Evidence for changes in healthcare use associated with transition to adult services is conflicting.
- Emergency Department visits increase and access to physiotherapy decreases at transition.
- There are marked differences between care patterns in the United States and Canada.

## Introduction

Life-limiting conditions (LLCs) include conditions that limit life—i.e. cause premature death, such as Duchenne muscular dystrophy, Batten’s disease and conditions that threaten life—i.e. may cause early death but may be cured, such as cancer or liver failure. There are increasing numbers of children and young people with LLCs.^1,2^ Although individual conditions are rare, there are many more children and young people with LLCs in the United Kingdom (approx. 85,000 in 2017/18^3^) than with diabetes mellitus (approx. 36,000 in 2018^4^) and at least 500,000 children living with similar conditions in the United States.^5^

Children with LLCs often receive specialist paediatric care before transitioning to adult services, typically from age 16 to 19 years in the United Kingdom^6^ and planned to be around age 18 years in the United States.^7^ There are many differences in delivery of paediatric and adult care, including relationship continuity and condition expertise.^6,8^ In childhood, allied health services, such as physiotherapy, are provided on an ongoing basis in the UK. There may be direct access to a specialist hospital ward when needed without having to first go through primary care or an Emergency Department. Care is often coordinated by the paediatric specialist with parents directly involved in decisions.^9^ Adults may have care coordinated by a primary care practitioner, with little experience of their condition and with whom they may have had little childhood contact. Allied health services are booked in blocks, with possible gaps in provision (there may be no equivalent adult service).^10^ Hospital access may require primary care or Emergency Department referral.^11^ Where the young people have capacity, they are expected to take more responsibility for care and care decisions, reducing parental involvement.^9^

The transition can be poorly defined and care may also continue in paediatric settings.^12^ Primary care transition training can be lacking, despite efforts at improvement.^8^ Transition can appear abrupt^6,13^ and support varies between conditions, but staff continuity, specialist adolescent clinics, good communication and planning are vital.^6^ Guidelines in the United Kingdom and United States highlight the challenges and need for planning.^9,14^ The United Kingdom Chief Medical Officer’s report^8^ into child health called for more research on transition for children with long-term conditions.

Systematic reviews have mainly focussed on transition experiences, interventions and biological indicators of disease progression, not healthcare use.^15–19^ Two reviews looked at experiences and found that many young people felt they lacked knowledge about their conditions, felt unprepared and feared that adult providers had similar limitations.^15,17^ One assessed loss to follow-up and lapse of care, reporting losses of between 7 and 61% depending on country and definition used.^15^ Other reviews considered healthcare use, but from the perspective of whether there was continuity of care, not quantifying differences in (for example) contacts post- versus pre-transition.^16,18^ These found poor continuity and no standardised transition with, instead, condition-specific programmes. Where biological indicators were assessed, they were commonly compared between intervention and control groups rather than post- versus pre-transition.^18,19^

In short, appraisal of the evidence for changes in healthcare post- versus pre-transition is lacking. Changes are likely to be important to young people and their families. Any increases in (for example) emergency hospital care may have negative implications for them through emotional trauma, disruption and (also, potentially, for health service providers) financial costs.^20–25^

This systematic review aimed to assess evidence for a change in health or social care use for young people with LLC post- compared to pre-transition. This information is needed to identify conditions and areas of care where use varies across the transition, useful for targeting future interventions and research.

## Methods

The protocol was registered on PROSPERO^26^ (ref. CRD42019156282.^27^). Reporting follows the Preferred Reporting Items for Systematic reviews and Meta-Analyses (PRISMA)^28^ and Synthesis Without Meta-Analysis^29^ guidelines.

### Eligibility criteria

Observational studies, randomised controlled trials and studies using quasi-experimental methods (e.g. interrupted time series, regression discontinuity) published in English from 1990 onwards were included if meeting the following criteria (presented in the Population, Exposure, Outcome format). Studies published before 1990 were excluded as widespread survival to adulthood in the LLC population is a relatively recent phenomenon, making older studies less relevant. Systematic reviews were not included, but for any reviews otherwise meeting inclusion criteria, the studies in the review were considered for inclusion.

#### Population

Children and young people with a LLC, including those treated in both paediatric and adult services (or, if assignment to post- and pre-transition groups was not explicit, including a range of ages spanning at least 15–19 years of age), in Organisation for Economic Co-operation and Development countries. The country limitation avoided comparisons with countries with potentially very different epidemiology and treatment of conditions—e.g. higher prevalence and poorer outcomes for HIV in many developing countries.^30^ Where a study included conditions that may or may not be LLC, depending on severity (e.g. cerebral palsy) only subgroups judged to have a LLC (if provided) were included.

#### Exposure

The transition from paediatric to adult healthcare services (i.e. in outpatient, inpatient and primary care).

#### Outcomes

Any measures of health or social care use, excluding those that were purely prescribing (e.g. intravenous antibiotic courses were of interest, oral antibiotic courses were not). Outcomes identified a priori were hospital admissions, bed days, Emergency Department visits, primary care visits and overall costs (direct costs to providers and direct or indirect costs to children and young people and their families).

#### Assignment to paediatric or adult groups

Study assignments to post- and pre-transition groups were used. It was planned, for any studies that neither assigned individuals to groups nor defined age of transition, to assign the group up to and closest to 16 years as pre-transition and the youngest group aged ≥19 as post-transition, based on common transition ages in the countries studied.^6–8^ However, these rules were not used as all included studies did their own assignment.

### Information sources

MEDLINE, EMBASE, CINAHL, PsychINFO and Social Science Citation Index were searched from 1 January 1990 to 27 April 2020 (date of last search). Forward and reverse citation searching was used for the included studies.

### Search strategy

The search strategy (supplementary material) was developed using the following concepts:

- LLC (using previously developed search strategies^31^)
  AND
- Children/young people (extended from published search strategies^14,15,19,32–35^)
  AND
- Transition (extended from published search strategies^14,15,19,32,34^)

### Study records

Records were de-duplicated using Endnote^36^ and uploaded to Covidence^37^ for screening.

### Selection process

One reviewer (S.W.J.) screened titles and abstracts against the eligibility criteria. A second reviewer (D.R.) independently screened 20%. Where disagreements occurred, records were retained for full-text screening.

Final selection used full-text records, with two reviewers (S.W.J. and D.R.) assessing all records. A third reviewer (L.K.F.) resolved disagreements.

### Data extraction and quality assessment process

One reviewer (S.W.J.) extracted data and assessed quality, with 30% checked by a fourth reviewer (D.G.-S., see “Acknowledgements”). Disagreements were resolved by discussion. Extracted data items are listed in the extraction form (Supplementary Material). Quality was assessed using a modified Newcastle–Ottawa scale^38^ (Supplementary Material) with one inapplicable question omitted (demonstration that the outcome of interest was not present at the beginning of the study).

### Summary measures

Outcome data were transformed to standardised measures where possible^39^: mean difference or incidence rate ratio where numbers of outcomes were reported and odds ratios where percentage risk or relative risk was reported. Some reported measures (medians, median differences) were not transformable.

### Data synthesis

A meta-analysis was planned,^27^ but studies were insufficiently comparable to undertake this (see “Results”). Alternative approaches were considered: summarising effect estimates^40^ was rejected for similar reasons to conducting a meta-analysis (study designs and measures were too heterogeneous). Instead, *p* value combination^40,41^ and vote counting^40^ were used. Data were synthesised by outcome as differences by outcome were of interest (i.e. not only whether care use changed but for which outcomes).

*p* Value combination summarises the strength of evidence for an effect by combining *p* values reported in studies with a common effect direction. Fisher’s *p* value^41^ was used as a summary measure, indicating whether at least one study showed evidence of an effect. This was visualised using albatross plots,^42^ which plot *p* value and effect direction against sample size. Visual guidelines show the expected plotting position of studies of differing sample sizes with the same effect size—if the true effect size of an intervention was a standardised mean difference of 0.1, then studies of different sizes would cluster around this guideline. Missing *p* values were estimated from 95% confidence intervals (CIs)^43^ or Fisher’s exact test.^44^

As not all studies provided *p* values or data from which these could be estimated, vote counting (counting studies showing effects in each direction, irrespective of statistical significance^45^) was also conducted. This was visualised using harvest plots,^46^ bar plots showing the number of studies (number of individual bars) reporting effects in each direction (irrespective of significance) and no effect and their quality scores (height of bar).

### Assessment of reporting bias and the strength of the body of evidence

Low numbers of studies with directly comparable effect measures (<10 for all outcomes^47^) prevented the use of funnel plots and associated statistical tests for reporting bias.

Overall strength of evidence was planned^27^ to be assessed using the GRADE framework.^48^ As no overall effect sizes were estimated, this was not done.

## Results

**Fig. 1:**
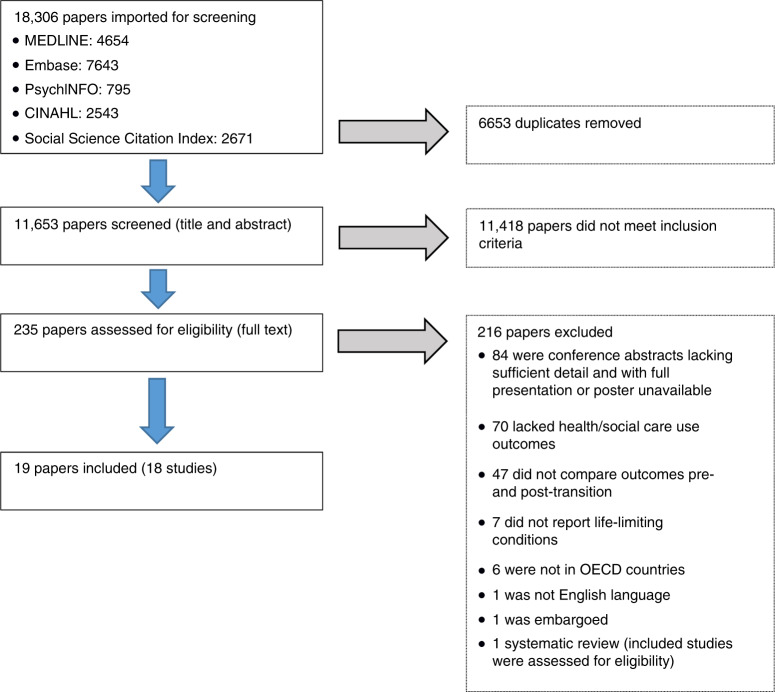
PRISMA diagram for screening and study selection. Blue arrows show progression of papers between stages and grey arrows show papers rejected at each stage. Numbers are shown from each database and for each reason for rejection at full text eligibility assessment.

Nineteen papers met inclusion criteria (Table 1) reporting on 18 observational studies—1 study was reported in 2 papers.^49,50^ One systematic review^51^ was also found to be relevant, but its relevant included studies had already been identified in the search and screening process. Figure 1 provides the PRISMA^28^ diagram.

**Table 1:** Characteristics of the included studies.

Study (ID)	Condition	Country	Setting	Design	Focus	Sample size	Groups compared	Outcomes	Measures	Transformed measures	Conclusions
Young et al.^49,50^ (01)	Cerebral palsy	Canada	6 treatment centres, Ontario	X-section	Age-related treatment patterns	1064	13–17 years (mean 15.3) versus 23–32 years (mean 26.4)	OP attendances ED visits IP admissions Bed days	Mean per person per year	IRR Mean difference	Decrease in OP attendances Decrease in IP admissions
Liljenquist et al.^52^ (02)	Cerebral palsy	USA	National cohort study	Historical longitudinal cohort	Physio during transition	35,290	Cohort: 13–16 years versus 21–26 years	Receipt of physiotherapy	% having visit per year	OR (but different and unknown time base)	Decrease in physiotherapy after transition
Roquet et al.^53^ (04)	Cerebral palsy	France	Brittany—survey	X-section (survey)	Healthcare use differences across transition	54	12–17 years versus 18–24 years	GP visits Receipt of physiotherapy	% having visit per year	OR	Decrease in rehabilitation service use
Duguépéroux et al.^65^ (04)	Cystic fibrosis	France	Single clinic	Longitudinal cohort	Clinical changes during transition	68	One year before transition versus 1 year after (median transition age 21 years)	OP attendances IV antibiotic courses Receipt of physiotherapy	Mean per person per year % having visit per year	IRR Mean difference OR	No negative impact
Tuchman and Schwartz^56^ (05)	Cystic fibrosis	USA	National registry	X-section (matched)	Health outcomes at transition	1322	Transitioned compared 1 year after transition to matched the non-transitioned group	IP admissions IV antibiotic courses	Mean per person per year	IRR Mean difference	No change
Collins et al.^58^ (06)	Cystic fibrosis	Australia	Single hospital	Longitudinal cohort	Hospital attendance at transition	44	Two years before transition versus 2 years after	OP attendances Bed days Days on IV antibiotics	Mean difference	Mean difference	Increase in OP visits, IP admissions and home IV antibiotic days
Crowley et al.^59^ (07)	Cystic fibrosis	USA	Single clinic	Longitudinal cohort	Association between social complexity and outcomes after transition	133	Two years before transition versus 2 years after	OP attendances Inpatient admissions	Mean per person per year	IRR Mean difference	Decrease in OP visits
Welsner^104^ (08)	Cystic fibrosis	Germany	Single hospital	Historical longitudinal cohort	Clinical changes during transition	39	1 year before transitions compared to 1 year after	Outpatient attendances Inpatient admissions	Mean per person per year	IRR Mean difference	Increase in OP visits and IP admissions
Biersteker^57^ (09)	HIV	USA	Single clinic	Longitudinal cohort	Outcomes of transition	25	1 year before transition compared to 1 year after	Outpatient attendances	Median per person per year	—	Decrease in OP attendance
Gray et al.^62^ (10)	HIV	USA	National registry	X-section	Care outcomes for those with HIV	3111	13–17 year olds versus 26+ years	Receipt of HIV care	% having visit per year	OR	Decrease in care
Akchurin et al.^64^ (11)	Renal	Canada	Single clinic	Historical longitudinal cohort	Medication adherence at transition	25	19–32 years versus to 9–17 years	OP attendances IP admissions ED visits	Mean per person per year	IRR Mean difference	Decrease in IP admissions Increase in ED visits
Pape et al.^67^ (12)	Renal	Germany	Single clinic	Longitudinal cohort	Comparing transition routes	59	1 year before transition to 1 year after	OP attendances	Mean per person per year	IRR Mean difference	No significant change
Samuel et al.^66^ (13)	Renal	Canada	National registry (excluding Quebec)	Historical longitudinal cohort	Hospital attendance at transition	92	15–18 years versus 19–21 years	IP admissions	Mean per person per year	IRR Mean difference	Decreasing IP admissions
Levine et al.^60^ (14)	Renal	USA	Multiple hospitals	X-section	Differences in healthcare use between children and adults	142	12–17 years versus 18–31 years	IP admissions ED visits Bed days	Model coefficients	IRR	Increase in IP admissions
Blinder et al.^61^ (15)	Sickle cell	USA	5 states’ routine records	X-section	Age-related treatment patterns	1113	>18 years versus ≤18 years (age range 0–50 overall)	OP attendances Total costs ED visits Bed days	Mean per person per year	IRR Mean difference	Increase in ED visits and bed days
Young et al.^63^ (16)	Spina bifida	Canada	Ontario—routine records	X-section	Age-related treatment patterns	284	13–17 years (mean 15.3) versus 23–32 years (mean 26.3)	OP attendances IP admissions ED visits	Mean per person per year	IRR Mean difference	Decrease in IP admissions Increase in ED visits
Cohen et al.^54^ (17)	Complex conditions	Canada	Ontario—routine records	Historical longitudinal cohort	Healthcare use at transition	2520	16–17 years versus 18–20 years	OP attendances IP admissions ED visits	Median per person per year	—	Increase in ED visits; decrease in IP admissions
Wijlaars et al.^55^ (18)	Blood/cancer	United Kingdom	England—routine records	X-section	Emergency admissions across transition	Unknown (for blood/ cancer disorders)	10–15 years versus 19–24 years	Emergency admissions	Mean per person per year	IRR Mean difference	Increase for females, decrease for males

Studies are ordered by condition and then publication date and numerical IDs provided in parentheses for each study are used consistently in tables and figures in this review (the included systematic review is not allocated an ID as it does not appear in other figures or tables).

*OP* outpatient, *IP* inpatient, *ED* Emergency Department, *IRR* incident rate ratio, *OR* odds ratio.

### Study characteristics

#### Settings

Studies took place in six countries: Australia, Canada, France, Germany, United Kingdom, and United States (Table 1), with 12 from North America. Settings ranged from single clinics and hospitals to regional or national analyses. Data sources were mostly routinely collected medical records but included one multiple wave longitudinal cohort study^52^ and one survey.^53^

#### Conditions studied

Six specific conditions were studied: renal conditions (end-stage kidney disease and kidney transplant recipients), HIV, sickle cell disease, cystic fibrosis, cerebral palsy, and spina bifida. Two studies^54,55^ looked at multiple conditions.

#### Study designs

Ten studies had longitudinal cohort designs (the same individuals compared post- and pre-transition) and eight were cross-sectional (different individuals compared post- and pre-transition). One of the latter^56^ used matching and one was a survey.^53^

#### Study quality

**Fig. 2:**
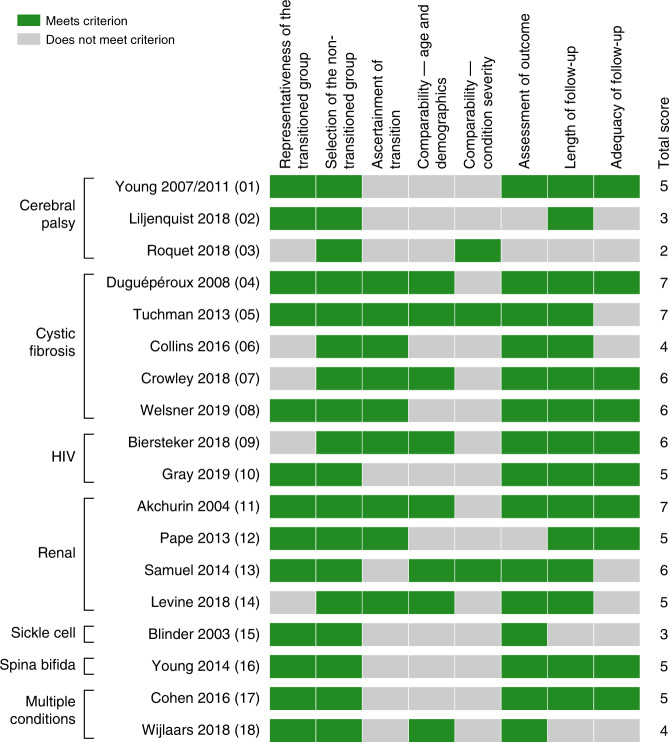
Quality scores on modified Newcastle–Ottawa scale (see supplement for detailed scoring criteria). Green indicates that a point was scored on each criterion, grey indicates that it was not. Studies, with numerical IDs in parentheses, and conditions studied are indicated to the left and overall scores to the right.

The observational studies varied in quality, scoring from 2 to 7 of a maximum of 8 on the customised Newcastle–Ottawa scale (Fig. 2). The scale assessed quality of studies for answering the review question—a low score does not necessarily mean that the study was of low quality for its own research question.

#### Representativeness of study population

Five studies^53,57–60^ used pre-transition groups that were likely not representative of the larger population with the condition (e.g. they used data from a single clinic). The populations included in these studies were likely to reflect the demographics (for example, ethnic group, deprivation level) of individuals geographically close to the clinic and—where care was provided under private insurance—in possession of appropriate health insurance, rather than the population in the country with the condition. Studies scoring 1 on this criterion used population data (e.g. routine medical records at the nation or state level) or described how participants were representative. All studies drew the post-transition group from the same population as the pre-transition group and so satisfied the second criterion of a representative (of the membership of the pre-transition group) post-transition group.

#### Ascertainment of transition

The majority of studies used age to allocate individuals to paediatric and adult groups. Nine^49,50,52–55,61–63^ did not provide justification (e.g. that transition invariably happened at that age), so misclassification bias was likely. Seven studies identified transition using clinical records and allowed individuals to transition at different ages.

#### Controlling for differences between groups

Eight studies^55–57,59,60,64–66^ controlled for age or, where relevant, other differences between groups. Only three^53,56,66^ controlled for severity of condition.

#### Ascertainment of outcomes and adequacy of follow-up

Most studies used medical records to measure outcomes; the three that did not^52,53,67^ relied on recollection by the young person or service provider. Follow-up was of sufficient length (at least 1 year both post- and pre-transition) in all but two studies,^53,55^ in which minimum follow-up was not stated. Follow-up was inadequate (>10% loss without analysis of potential bias) in 8 studies.^52,53,55,56,58,60,61,66^

### Outcomes

Reported outcomes were: outpatient attendances, inpatient admissions, Emergency Department visits, inpatient bed days, intravenous antibiotic courses, physiotherapy, HIV care, General Practice contact, and healthcare costs (Supplemental Table 1).

### Data synthesis

A meta-analysis was not conducted as the studies were too heterogeneous in designs (cross-section versus cohort), study populations (North America versus Europe; widely different age ranges in the groups compared) and outcome measures (mean differences, median differences, incidence rate ratios) to provide enough comparable studies for any outcome.

#### Outpatient attendances

**Fig. 3:**
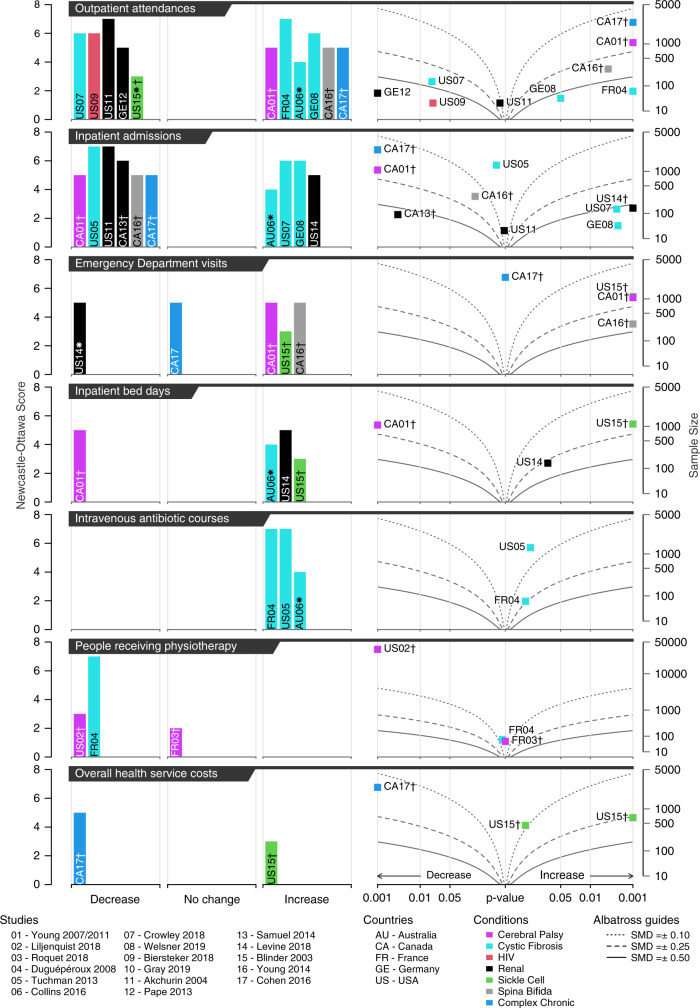
Harvest plots (left) and albatross plots (right) for the indicated outcomes. Labels, e.g. CA-01, indicate country and numerical study ID. For albatross plots, *p* values < 0.001 are plotted at 0.001. Dagger (†) in the harvest and albatross plots indicates studies that did not provide justification for assignment to post- and pre-transition groups. In the harvest plots, asterisk (✱) on a bar indicates a study not included on the corresponding albatross plot (as the *p* value could not be determined). Curved albatross guidelines are illustrative of the standardised mean difference (SMD) that would give rise to a given *p* value for a given sample size equally split between post- and pre-transition observation.

Eleven studies reported outpatient attendance, five showed a lower number post- compared to pre-transition and six showed a higher number (Fig. 3). Quality of studies varied, with Newcastle–Ottawa scores (NOSs) of 3–7. *p* values were derivable for ten studies. There was strong evidence for both a reduction in at least one study (Fisher’s *p* < 0.001) and an increase (Fisher’s *p* < 0.001). Canadian studies all showed an increase while studies from the United States all showed a decrease. Studies that did not justify assignment to post- and pre-transition groups showed larger increases in outpatient attendance than most studies that did justify assignment.

#### Inpatient admissions

Ten studies looked at inpatient admissions. Six showed a decrease post-transition; four showed an increase (Fig. 3). Studies with results in both directions were of moderate-to-high quality (NOS = 4–7). *p* values were provided or derivable for nine. There was strong evidence for both a reduction (Fisher’s *p* < 0.001) and an increase (Fisher’s *p* < 0.001). Canadian studies all showed a decrease; United States studies showed increases and decreases, but only strong evidence for an increase (Fisher’s *p* values: increase <0.001; decrease 0.87). Most studies showing a decrease post- versus pre-transition did not justify assignment to the post- and pre-transition groups.

#### Emergency Department visits

Five studies looked at changes in Emergency Department visits. Three showed an increase, one a decrease^60^ and one no change^54^ (Fig. 3). Studies were of moderate quality (NOS = 3–5). *p* values were derivable for four studies. There was strong evidence for an increase in Emergency Department visits (Fisher’s *p* < 0.001). Two of the three studies showing an increase did not justify assignment to the post- and pre-transition groups.

#### Inpatient bed days

Four studies looked at inpatient bed days. Three showed an increase and one a decrease (Fig. 3). Studies were of moderate quality (NOS = 3–5). *p* values were derivable for three studies and there was evidence for a decrease (Fisher’s *p* = 0.018) and an increase (Fisher’s *p* = 0.002).

#### Intravenous antibiotic courses

Three studies reported numbers of intravenous antibiotic courses, all showing an increase post-transition (Fig. 3). The studies were of moderate to high quality (NOS = 4–7). *p* values were derivable in two studies; there was no strong evidence for the observed increases (Fisher’s *p* = 0.103).

#### Physiotherapy

Three studies reported percentages of individuals in receipt of physiotherapy, with two showing a decrease and one showing no change (Fig. 3). Study quality ranged from low to high (NOS = 2–7). *p* values were derivable for all studies and there was evidence of a decrease in provision of physiotherapy (Fisher’s *p* = 0.002).

#### Costs

Only two studies reported differences in total healthcare costs, one showing lower and one higher costs post-transition (Fig. 3). Both studies were of moderate quality (NOS = 3–5). *p* values were derivable for both. A single study^61^ that splits effects by receipt of iron chelation therapy is shown as two points in the albatross plot but as a single bar in the harvest plot. There was strong evidence of lower (Fisher’s *p* = 0.016) and higher costs (Fisher’s *p* = 0.005).

#### Other outcomes

One study^55^ reported emergency admissions. This showed a significant increase for females post-transition and a significant decrease for males (Supplemental Table 1). This study was not included in the inpatient admission albatross plot due to a lack of information on sample size and omitted from the harvest plot because it could not be categorised as showing an increase or decrease (having subgroups showing both).

Another study^62^ reported percentages of individuals in receipt of any HIV care and showed a decrease post-transition (Supplemental Table 1).

General Practitioner visits were reported in one study,^53^ which showed that 72% of individuals visited post-transition and 68% pre-transition (odds ratio 1.2, 95% CI 0.3–4.5, Supplemental Table 1).

## Discussion

### Summary of evidence

The included studies provide conflicting evidence on outpatient attendances, inpatient admissions, inpatient bed days and health service costs (for these outcomes, there was evidence for both increases and decreases post- versus pre-transition). There was greater consistency within countries than between them, where sufficient studies were available to gauge this. There was no evidence for changes in the numbers of intravenous antibiotic courses; there was evidence for both an increase in Emergency Department visits and a reduction in physiotherapy post- versus pre-transition.

### Comparisons with other chronic conditions and implications for quality of care

Beyond LLC, for individuals with chronic conditions, there is evidence of disengagement and decreased outpatient attendance after transition.^15,32,68^ This is particularly true for people with diabetes, where an increase in inpatient admissions is also observed,^69,70^ although this varies with continuity of care.^71^ The present review, however, reports a more mixed picture, with evidence emerging for both increased and decreased outpatient attendance. Disengagement may be driven by a perception that further care is unnecessary^72^ and people with milder pre-transition sickle cell disease have lower transition success.^73^ Disengagement may be less likely for people with a LLC due to the severity of their condition necessitating regular contact and due to many young adults with a LLC lacking capacity to decide whether they will or will not attend an appointment, this decision resting instead—as during childhood—with the carer. Implications for care may vary—changes in attendance may reflect different organisation of services post- compared to pre-transition, e.g. paediatrician visits where several different symptoms such as epilepsy and reflux were managed at once may be replaced with multiple outpatient visits to different specialists in adulthood. If either self-care (if appropriate) or primary care partly replace outpatient attendance after transition, the numbers of attendances may decrease. The studies did not report on the levels of attendance at outpatient appointments, which have been shown to be associated with use of emergency hospital care.^74^

The post-transition decrease in inpatient admissions for some included studies was unexpected—it is often anticipated that condition severity (and need for inpatient care) increases with age, independent of transition. However, planned admissions decrease after transition for a range of chronic conditions,^75^ which may explain observed reductions in overall inpatient admissions in some included studies—most reported only overall admissions, not differentiating between emergency and planned. Increasing numbers of adults with chronic conditions are treated in children’s hospitals,^76^ which may suggest that suitable care is less available in adult inpatient settings or that transition is delayed for those most in need of inpatient care. An increase in emergency inpatient admissions post-transition may be indicative of worse condition management, but only one study looked at this and reported an increase for women but a decrease for men.^55^

For those with chronic conditions, Emergency Department visits are known to increase at transition ages,^77,78^ and the limited evidence in this review is consistent with this. This may be linked to the transition, e.g. young people seeking hospital rather than primary care post-transition.^11,79^ Alternatively, it may be due to natural progression of condition severity^80^ or increased risk-taking behaviours at the age of transition^78^ (those with long-term conditions are as likely as the those without long-term conditions to increase risk-taking behaviours at this age.^81^). Increases in Emergency Department visits are important as adverse care experiences may discourage future adult service engagement.^79^

Inpatient bed days depend on the combination of inpatient admissions and length of stay. Influences on the numbers of admissions discussed above are relevant—there are reasons to both expect more inpatient bed days (due to condition progression and potentially worse outpatient care) and fewer (due to fewer planned admissions^75^ and potentially shorter stays in adult settings.^82^)

Reduced adherence to medications is observed in late adolescence and with the transition to adult services for many conditions.^83–85^ Antibiotic use is particularly associated with cystic fibrosis to manage infection and is not necessarily indicative of worse outcomes.^86,87^ However, the numbers of intravenous courses of antibiotics increased in the included studies post-transition and one study reported a fall in oral antibiotic use,^65^ which may indicate worse condition management—oral antibiotics are preferred in the first instance.^88^ Two of the included studies showed increases in home administration of antibiotics,^56,65^ which may be appropriate but may indicate a lack of clinical oversight.

Lapses in physiotherapy after transition have long been identified as an issue for children with chronic conditions,^14,89^ so support for this within the included studies is unsurprising. Increases in physiotherapy at home, either alone or with parents, as reported in one study^65^ may be appropriate if sufficient training is given^90^ but may also reflect a lack of service access.

Overall costs may reflect both differences in provider costs and differences in healthcare use. It has been shown that costs for adults admitted to children’s hospitals are higher than for those treated in adult hospitals.^82^ Increases may reflect more expensive emergency/hospital care rather than cheaper preventative care in the community or an outpatient setting.^91^ Decreases in costs may indicate more efficient service delivery or reduced access to services. Reflecting this, the evidence on healthcare costs in the included studies was conflicting.

### Variations between countries

Most countries (excepting the United States and Canada) had no more than two studies reporting the same outcome, so no conclusions could be drawn on differences between those countries. Studies from the United States and Canada showed opposite evidence on outpatient attendances (United States: decrease post- versus pre-transition; Canada: increase) and inpatient admissions (United States: increase; Canada: decrease).

Increased outpatient attendances may reflect organisational changes at transition, with multiple adult specialists replacing one paediatric specialist, particularly in Canada, where there are few multi-specialist clinics.^92,93^ It may also reflect differences in funding and access, with Canada having a single-payer system while United States funding is a mix of public and private health insurance.^92^ Insurance type has been associated with different healthcare use for children with a LLC^60^ and adolescents with special healthcare needs are at higher risk of losing public insurance cover on reaching adulthood^94^ (although insurance type has not been linked with transition success.^73^).

Differences in inpatient admissions may be due to differences in disease progression—survival times for cystic fibrosis are greater in Canada than in the United States.^95^ They may also reflect differing demographics—it has been shown that some groups in the US have lower utilisation of outpatient services but higher inpatient use, irrespective of access to healthcare funding.^80^

The few studies looking at inpatient bed days aligned with those for inpatient admissions, with the Canadian study showing a reduction post-transition (despite reporting increased length of stay)^49,50^ and the United States studies showing an increase.^60,61^

### Variations between methods of ascertainment of transition

Results varied for some outcomes between studies that justified assignment of individuals to the post- or pre-transition groups and studies that did not. Misclassification may explain these differences but would be expected to result in smaller observed changes post- versus pre-transition (due to mixing of the groups) rather than the larger differences or apparent reversal of effect direction observed. Many of the studies not justifying assignment were from Canada and the observed differences largely reflect those between Canada and the United States. Country differences therefore seem more plausible than misclassification bias in explaining the observed variation. One Canadian study^54^ justified assignment by stating that, apart from primary care, transition invariably happened by age 18 years, so other Canadian studies may have assigned post- and pre-transition groups correctly based on this age cut-off, despite not justifying this.

### Variations between conditions

Comparisons between conditions were limited as few conditions had more than two studies reporting the same outcome. Cystic fibrosis studies more commonly reported increases in healthcare use post-transition than decreases—cystic fibrosis has well-established transition programmes aimed at maintaining engagement.^87^ The reverse was true for renal conditions, for which studies were largely concerned with paediatric transplant recipients, where many problems have been identified with transition and ongoing engagement in care.^96^

### Contribution of this review

The existing systematic review on healthcare use^51^ had a narrower focus than the present review, including only studies on cystic fibrosis. It found no significant differences in the number of antibiotic therapies and hospital attendance, but an increase in outpatient appointments. The included papers reporting these outcomes were also included in the present study.

The present review is the first to assess evidence across a range of LLCs. It reveals conflicting evidence, some of it patterned by country, suggestive of different healthcare regimes delivering different changes at transition. It finds that assessments of changes in healthcare use at the transition have been inconsistent, with many different outcome measures, designs and methods of ascertaining transition. It highlights the need for more high-quality research in this area.

### Limitations

#### Included studies

As demonstrated by the NOSs, quality of the included studies varied. Half failed to determine whether individuals were in paediatric or adult care, instead using a simple age cut-off. This was appropriate to the research questions of some studies, but a limitation for the review due to potential misclassification bias.

Most studies failed to separate possible effects of transition, age and condition severity. Only one study^56^ propensity-score matched post and pre-transition groups and two^53,66^ controlled for condition severity. The majority did not control for age, so may have measured effects associated with age and condition progression, rather than transition. There is a need for research that separates associations with age from associations with receipt of either paediatric or adult care.

Differences in age ranges of compared groups limited comparability between studies and were one reason a meta-analysis was not conducted. This reflected differences in the age at which transition takes place, both between and within countries.^15,18,51^ Many studies used means and standard deviations to summarise event counts, even though these were likely to be highly skewed, following a Poisson or negative-binomial distribution. Outcomes measured varied greatly, as observed in reviews of transition for chronic conditions,^18,51,97^ limiting comparability.

Finally, the included studies were concentrated on only a small number of the more common LLCs. In particular, neurological conditions, affecting 12% of children with a LLC^1^ and one of the largest groups referred for paediatric palliative care,^98,99^ were under-represented. The generalisability of findings across LLCs as a whole is therefore questionable.

#### Review limitations

The study limitations prevented a meta-analysis or other effect-size pooling. The synthesis used was appropriate^29^ but explored directions of effect and strength of evidence; no conclusions could be drawn on effect size.

There is no definitive list of LLCs and whether a condition is included can depend on severity (e.g. cerebral palsy). Study inclusion in this review depended on the authors’ judgement, based on previous work developing lists of LLCs.^1^ Other reviewers might come to different decisions on some studies. Presentation of the results categorised by condition does, however, aid the reader in gauging what effect excluding, for example, cerebral palsy or sickle cell disease would have.

Transition is not always clearly defined, even at the individual level, with some adults continuing to receive some care in paediatric settings. However, the transition is experienced as a very real phenomenon by young people and their families.^6,9,10,13^ Definitions used in many of the studies were based on well-defined cut-offs, such as transfer from a paediatric to an adult clinic.

The search strategy for this review extended previously developed search strategies and was designed to maximise sensitivity. Although initial title and abstract screening was done by one reviewer (with 20% checked by a second reviewer), full-text review was carried out by two reviewers independently. There is a risk of missed relevant studies among the 80% of titles and abstracts screened by only one reviewer,^100,101^ but in the 20% of titles and abstracts that were screened by a second reviewer, no studies rejected by the first reviewer were ultimately included in the review.

Eighty-four conference abstracts were excluded due to the full presentation or poster being unavailable. One study (a doctoral thesis^102^) was excluded as the full text was embargoed. It is possible that inclusion of these, had full-text been available, would have altered conclusions.

### Future research

There has been widespread recognition of the need for improvement of the transition and suggestions of how his might be done.^18,19,91,103^ However, more high-quality research is needed that analyses healthcare use across the full range of LLCs to better target interventions with potential to be cost-effective. In particular, the present research in this area is lacking in the following areas:

- possible misclassification bias due to poor ascertainment of transition;
- inconsistent and sometimes inappropriate outcome measurements, e.g. the use of mean values to describe highly skewed distributions;
- a lack of control for potential confounders, particularly increasing severity of condition with increasing age, irrespective of transition status.

Research is needed that more directly links transition and healthcare use, through analysis of the time periods immediately before and after transition and the use of methods enabling causal inference.

## Conclusions

The evidence on changes in healthcare use post- versus pre-transition from paediatric to adult healthcare is mixed and conflicting. There is some evidence of an increase in Emergency Department visits and a reduction in access to physiotherapy but different patterns were identified for outpatient attendances and inpatient admissions between the United States and Canada. This may be linked to differences in organisation and funding of healthcare services, and in at least some populations, significant changes in healthcare use were observed at the transition.

More high-quality research is needed to provide a stronger evidence base for the extent to which widely reported concerns about the transition are reflected in changes in healthcare use and resource utilisation.

## Supplementary Information

Supplementary Material

PRISMA 2009 Checklist

Synthesis Without Meta-analysis (SWiM) reporting item
