# Supplementary material for: Transition of children with life-limiting conditions to adult care and healthcare use: a systematic review
Source: Pediatr Res. 2021 Mar 2;90(6):1120–31. doi: 10.1038/s41390-021-01396-8 (PMC8671088; doi:10.1038/s41390-021-01396-8)
Supplement: Supplementary file 1 — Supplementary Material [file 41390_2021_1396_MOESM1_ESM.pdf]

Healthcare use when people with life-limiting conditions transition to adult care - a systematic review

Supplemental Material

Contents

Supplemental results 2

Search strategy 6

Concepts 6

Basis of search strategies 6

LLC 6

Child/young adult 6

Transition 6

References 6

MEDLINE (Ovid) 7

Embase (Ovid) 23

Psychinfo (Ovid) 38

CINAHL (EBSCOHost) 50

Social Sciences Citation Index (Web of Science) 72

Data extraction form 87

Modified Newcastle-Ottawa Scale 89

Supplemental results

Supplemental table 1: Studies by outcome (with numerical study ID in parentheses), with direction of effect and effect size. ↓ = decrease ↑= increase ↔=no difference

Study	Condition	Direction of effect	Measure	Sample size	Effect size (95% CI) [per person per year, unless stated]	P-value
Outpatient attendances						
Young 2007/2011 (01)	Cerebral Palsy	↑	Means of groups	1064	0.89 (0.36-1.42)	<0.001
Duguépéroux 2008 (04)	Cystic Fibrosis	↑	Means of groups	68	1.9 (0.8-3.0)	<0.001
Collins 2016 (06)	Cystic Fibrosis	↑	Mean difference	44	2.92	-
Crowley 2018 (07)	Cystic Fibrosis	↓	Means of groups	133	0.4 (0.1-0.7)	0.02
Welsner 2019 (08)	Cystic Fibrosis	↑	Means of groups	39	0.74 (0.01-1.48)	<0.05
Biersteker 2018 (09)	HIV	↓	Medians of groups	25	3.0	0.02
Akchurin 2004 (11)	Renal	↓	Medians of groups	25	0.8	0.75
Pape 2013 (12)	Renal	↓	Means of groups	59	9.0 (3.7-14.3)	<0.001
Blinder 2003 (15)	Sickle Cell	↓	Means of groups	663	0.41 (0.21-0.62)	<0.001
Young 2014 (16)	Spina Bifida	↑	Means of groups	284	0.49 (0.16-0.82)	0.004
Cohen 2016 (17)	Complex chronic conditions	↑	Medians of groups	2520	1.0	<0.001
Inpatient admissions						

Young 2007/2011 (01)	Cerebral Palsy	↓	Means of groups	1064	0.8 (0.3-1.3)	<0.001
Tuchman 2013 (05)	Cystic Fibrosis	↓	Mean difference	1322	0.02 (-0.06-0.10)	0.62
Collins 2016 (06)	Cystic Fibrosis	↑	Mean difference	44	1.71	-
Crowley 2018 (07)	Cystic Fibrosis	↑	Means of groups	133	0.30 (0.11-0.49)	0.002
Welsner 2019 (08)	Cystic Fribrosis	↑	Means of groups	39	1.0 (0.4-1.6)	0.002
Akchurin2004 (11)	Renal	↓	Medians of groups	25	0.25	0.95
Samuel 2014 (13)	Renal	↓	Incidence rate ratio	92	0.71 (0.57-0.90) [IRR]	0.003
Levine 2018 (14)	Renal	↑	Incidence rate ratio	142	3.7 (1.7-8.0) [IRR]	<0.001
Young 2014 (16)	Spina Bifida	↓	Means of groups	284	0.04 (-0.02-0.10)	0.196
Cohen 2016 (17)	Complex chronic conditions	↓	Medians of groups	2520	0	<0.001 (Medians both zero but statistically different)
Emergency inpatient admissions						
Wijlaars 2018 (18)	Blood/cancer – male	↓	Incidence rate ratio	Not known	1.20 (1.17-1.23) [IRR]	<0.001
	Blood/cancer - female	↑	Incidence rate ratio	Not known	0.92 (0.89-0.96)[IRR]	<0.001
Emergency Department visits						
Young 2007/2011 (01)	Cerebral Palsy	↑	Means of groups	1064	0.55 (0.22-0.88)	<0.001
Levine 2018 (14)	Renal	↓	Incidence rate ratio	142	0.35 [IRR]	-

Blinder 2003 (15)	Sickle Cell	↑	Means of groups	663	1.44 (0.59-2.29)	<0.001
Young 2014 (16)	Spina Bifida	↑	Means of groups	284	0.07 (0.03-0.11)	<0.001
Cohen 2016 (17)	Complex chronic conditions	↔	Medians of groups	2520	0	0.14
Inpatient bed days						
Young 2007/2011 (01)	Cerebral Palsy	↓	Means of groups	1064	0.37 (0.15-0.59)	<0.001
Collins 2016 (06)	Cystic Fibrosis	↑	Mean difference	44	1.71	-
Levine 2018 (14)	Renal	↑	Incidence rate ratio	142	4.14 (0.76-22.23) [IRR]	≤0.1
Blinder 2003 (15)	Sickle Cell	↑	Means of groups	663	20.48 8.33-32.63)	<0.001
Intravenous antibiotic courses						
Duguépéroux 2008 (04)	Cystic Fibrosis	↑	Means of groups	68	0.3 (-7.9-9.7)	0.333
Tuchman 2013 (05)	Cystic Fibrosis	↑	Mean difference	1322	0.04 (-0.03-0.11)	0.256
Collins 2016 (06)	Cystic Fibrosis	↑	Mean difference	44	1.96	-
Physiotherapy						
Liljenquist 2018 (02)	Cerebral Palsy	↓	% of persons receiving therapy	35290	0.35 (0.34-0.36) [OR]	<0.001
Roquet 2018 (03)	Cerebral Palsy	↔	% of persons receiving therapy	54	1 [OR]	-
Duguépéroux 2008 (04)	Cystic Fibrosis	↓	% of persons	68	0.88 (0.40-1.91) [OR]	0.855

			receiving therapy			
HIV care						
Gray 2019 (10)	HIV	↓	% of persons receiving care	3111	0.61 (0.51-0.72) [OR]	<0.001
General practitioner visits						
Roquet 2018 (03)	Cerebral Palsy	↑	% of persons having a visit	54	1.2 (0.3-4.5) [OR]	0.77
Overall healthcare costs						
Blinder 2003 (15)	Sickle Cell (patients receiving iron chelation therapy)	↑	Means of groups	450	1916 (-1938-5770) [$US]	0.335
	Sickle Cell (patients not receiving iron chelation therapy)	↑	Means of groups	663	7749 (3151-12345) [$US]	<0.001
Cohen 2016 (17)	Complex chronic conditions	↓	Medians of groups	2520	893 [$CAN]	<0.001

Search strategy

Concepts

LLC AND Child/young adult AND Transition

Fourth concept, health and social care, not searched

Basis of search strategies

LLC

Based on two searches developed by YHEC (Arber 2014).

Child/young adult

Developed from McPheeters, Davis et al. (2014), Rachas, Tuppin et al. (2018), Prior, McManus et al. (2014), Heery, Sheehan et al. (2015), Leclercq, Leeflang et al. (2013), National Institute for Health and Care Excellence (2016) and Association pour l'avancement des sciences et des techniques de la documentation (2018).

Transition

Developed from McPheeters, Davis et al. (2014), Rachas, Tuppin et al. (2018), Prior, McManus et al. (2014), Heery, Sheehan et al. (2015) and National Institute for Health and Care Excellence (2016).

References

Arber, M. (2014). Literature Searches for Rapid Review of Care and Support Needs of Children with a Life-Limiting Condition and Their Families - Search Report. York, UK, York Health Economics Consortium.

Association pour l'avancement des sciences et des techniques de la documentation. (2018). "Adolescents et jeunes adultes." Retrieved 10 July 2019, from http://extranet.santecom.qc.ca/wiki/!biblio3s/doku.php?id=Accueil.

Heery, E., A. M. Sheehan, A. E. While and I. Coyne (2015). "Experiences and Outcomes of Transition from Pediatric to Adult Health Care Services for Young People with Congenital Heart Disease: A Systematic Review." Congenital Heart Disease **10**(5): 413-427.

Leclercq, E., M. M. G. Leeflang, E. C. van Dalen and L. C. M. Kremer (2013). "Validation of Search Filters for Identifying Pediatric Studies in PubMed." The Journal of Pediatrics **162**(3): 629-634.e622.

McPheeters, M., A. M. Davis, J. L. Taylor, R. F. Brown, S. A. Potter and J. R. Epstein (2014). Transition care for children with special health needs. Effective Halthcare Program Technical Brief Number 15, Agency for Healthcare Research and Quality.

National Institute for Health and Care Excellence (2016). Transition from children's to adults' services for young people using health or social care services.

Prior, M., M. McManus, P. White and L. Davidson (2014). "Measuring the "Triple Aim" in Transition Care: A Systematic Review." Pediatrics.

Rachas, A., P. Tuppin, L. Meyer, B. Falissard, A. Faye, N. Mahlaoui, E. de La Rochebrochard, M. Frank, P. Durieux and J. Warszawski (2018). "Excess mortality and hospitalizations in transitional-age youths with a long-term disease: A national population-based cohort study." PLOS ONE **13**(3): e0193729.

MEDLINE (Ovid)

Concepts:

1. LLC: lines 1-583
2. Child/young adult: lines 584-595
3. Transition: lines 596-607

1	Creutzfeldt-Jakob Syndrome/
2	(creutzfeldt-jakob$ or jakob-creutzfeldt$ or cjd or spongiform encephalopath$).ti,ab,kf.
4	(subacute sclerosing panencephalit$ or sub-acute sclerosing panencephalit$ or sspe or subacute sclerosing leukoencephalit$ or sub-acute sclerosing leukoencephalit$ or van bogaert$ leukoencephalit$ or measles inclusion body encephalit$ or mibe).ti,ab,kf.
5	beta-Thalassemia/
6	(beta adj (thalass?emi$ or thalas?emi$)).ti,ab,kf.
7	((thalass?emi$ or thalas?emi$) adj major).ti,ab,kf.
8	exp Anemia, Aplastic/
9	((hypoplastic or aplastic) adj an?emi$).ti,ab,kf.
10	(medullary adj3 hypoplas$).ti,ab,kf.
11	exp Neutropenia/
12	((severe or chronic$) adj3 neutropeni$).ti,ab,kf.
13	immunologic deficiency syndromes/ or acquired immunodeficiency syndrome/
14	(immun$ deficiency adj (syndrome$ or disease$ or disorder$)).ti,ab,kf.
15	(immunodeficiency adj (syndrome$ or disease$ or disorder$)).ti,ab,kf.
16	DiGeorge Syndrome/
17	(digeorge$ or di george$ or sedlackova$ or opitz g-bbb or velocardiofacial or velo-cardiofacial or velo-cardio-facial or shprintzen$ or ctaf).ti,ab,kf.
18	((deletion or vcf or pharyngeal pouch or thymic aplasia or anomaly face) adj (syndrome$ or disease$ or disorder$)).ti,ab,kf.
19	Common Variable Immunodeficiency/
20	((common variable or late onset) adj3 (immunodeficienc$ or immune deficienc$ or immunoglobulin deficienc$ or hypogammaglobulin$)).ti,ab,kf.
21	acquired hypogammaglobulin$.ti,ab,kf.
22	Cryoglobulinemia/
23	cryoglobulin?em$.ti,ab,kf.
24	Polyendocrinopathies, Autoimmune/
25	((autoimmune or failure$) adj3 (polyglandular$ or polyendocrin$)).ti,ab,kf.
26	Progeria/
27	(progeria or hutchinson-gilford$).ti,ab,kf.
28	Tyrosinemias/
29	tyrosin?em$.ti,ab,kf.
30	Maple Syrup Urine Disease/
31	(maple syrup urine or msud).ti,ab,kf.
32	branched chain.ti,ab,kf.
33	(bckd adj5 (deficienc$ or ketoacid$ or keto-acid$)).ti,ab,kf.
34	hyperleucine-isoleucin$.ti,ab,kf.

35	Methylmalonic Acid/
36	(methylmalonic acid?emi$ or methylmalonic aciduri$ or methyl malonic acid?emi$ or methyl malonic aciduri$).ti,ab,kf.
37	Propionic Acidemia/
38	(propionic acid?em$ or propionic acidur$ or propionyl-CoA carboxylase deficienc$ or ketotic glycin?em$).ti,ab,kf.
39	Adrenoleukodystrophy/
40	(adrenoleukodystroph$ or x-ald or schilder-addison$ or addison-schilder$ or adrenomyeloneuropath$).ti,ab,kf.
41	Carnitine O-Palmitoyltransferase/
42	((carnitine palmityltransferase or carnitine palmitoyltransferase or carnitine o-palmityltransferase or carnitine o-palmitoyltransferase) adj3 deficienc$).ti,ab,kf.
43	Fanconi Syndrome/
44	(fanconi$ adj (syndrome$ or disease$ or disorder$)).ti,ab,kf.
45	(ocular adj3 (renal or kidney)).ti,ab,kf.
46	Cystinosis/
47	(cystinos$ or cystine storage or cystine diathes$ or cystine disease$).ti,ab,kf.
48	Oculocerebrorenal Syndrome/
49	((lowe or lowes or oculocerebrorenal or cerebrooculorenal or cerebro-oculo-renal) adj3 (syndrome$ or disease$ or disorder$)).ti,ab,kf.
50	Metalloproteins/df
51	Molybdenum/df
52	(molybdenum cofactor deficien$ or molybdenum co-factor deficien$).ti,ab,kf.
53	Oxidoreductases Acting on Sulfur Group Donors/df
54	Sulfite Oxidase/df
55	((sulphite$ or sulfite$) adj3 oxidase deficien$).ti,ab,kf.
56	Argininosuccinic Acid/
57	(argininosuccinic acidur$ or argininosuccinic acid?emi$).ti,ab,kf.
58	Citrullinemia/
59	(citrullin?emi$ or citrullinuri$).ti,ab,kf.
60	Amino Acid Metabolism, Inborn Errors/
61	(glutaric acid?emi$ or glutaric aciduri$).ti,ab,kf.
62	Hyperglycinemia, Nonketotic/
63	(glycine encephalopath$ or non-ketotic hyperglycin?emi$ or nonketotic hyperglycin?emi$).ti,ab,kf.
64	Hyperargininemia/
65	(arginin?emi$ or arginase deficien$ or hyperarginin?emi$).ti,ab,kf.
66	Renal Aminoacidurias/
67	(aminoaciduri$ or aminoacid?emi$).ti,ab,kf.
68	exp glycogen storage disease/
69	(glycogen storage adj (disease$ or syndrome$ or disorder$)).ti,ab,kf.
70	(pompe$ adj (disease$ or syndrome$ or disorder$)).ti,ab,kf.
71	Galactosemias/
72	galactos?emi$.ti,ab,kf.
73	Pyruvate Dehydrogenase Complex Deficiency Disease/
74	(pyruvate dehydrogenase adj3 deficien$).ti,ab,kf.
75	(oxalosis and (renal or kidney$)).ti,ab,kf.
76	exp Gangliosidoses/
77	gangliosidos$.ti,ab,kf.
78	(sandhoff$ adj (disease$ or syndrome$ or disorder$)).ti,ab,kf.

79	tay sach$.ti,ab,kf.
80	Mucolipidoses/
81	mucolipidos$.ti,ab,kf.
82	Canavan Disease/
83	(canavan$ leucodystroph$ or aspartoacylase deficien$ or aminoacylase 2 deficien$).ti,ab,kf.
84	((canavan$ or canavan-van bogaert-bertrand$) adj (disease$ or syndrome$ or disorder$)).ti,ab,kf.
85	Gaucher Disease/
86	(gaucher$ adj (disease$ or syndrome$ or disorder$)).ti,ab,kf.
87	(glucocerebrosidase deficien$ or glucosylceramidase deficien$).ti,ab,kf.
88	Leukodystrophy, Metachromatic/
89	(metachromatic leukodystroph$ or arylsulfatase A deficien$ or metachromic leukodystroph$).ti,ab,kf.
90	exp Niemann-Pick Diseases/
91	(niemann-pick$ or sphingomyelinase deficien$).ti,ab,kf.
92	Sphingolipidoses/
93	sphingolipidos$.ti,ab,kf.
94	Fabry Disease/
95	(fabry$ adj (disease$ or syndrome$ or disorder$)).ti,ab,kf.
96	(angiokeratoma corporis diffusum or alpha-galactosidase A deficien$).ti,ab,kf.
97	Leukodystrophy, Globoid Cell/
98	(krabbe$ adj (disease$ or syndrome$ or disorder$)).ti,ab,kf.
99	(globoid cell leukodystroph$ or galactosylceramide lipidos$ or galactosylcerebrosidase deficien$ or galactosylceramidase deficien$).ti,ab,kf.
100	Farber Lipogranulomatosis/
101	(farber$ adj (disease$ or syndrome$ or disorder$)).ti,ab,kf.
102	(farber$ lipogranulomatos$ or ceramidase deficien$ or fibrocytic dysmucopolysaccharidos$).ti,ab,kf.
103	Pelizaeus-Merzbacher Disease/
104	pelizaeus-merzbacher$.ti,ab,kf.
105	Sulfatases/df
106	Multiple Sulfatase Deficiency Disease/
107	(sulfatase deficien$ or sulphatase deficien$ or mucosulfatidos$).ti,ab,kf.
108	(austin$ adj (disease$ or syndrome$ or disorder$)).ti,ab,kf.
109	sulfatidosis/
110	sulfatidos$.ti,ab,kf.
111	Sea-Blue Histiocyte Syndrome/
112	sea-blue histiocyt$.ti,ab,kf.
113	Neuronal Ceroid-Lipofuscinoses/
114	(batten$ adj (disease$ or syndrome$ or disorder$)).ti,ab,kf.
115	(neuronal ceroid lipofuscinos$ or santavuori-haltia$ or jansky-bielschowsky$ or bielschowsky-jansky$).ti,ab,kf.
116	(kuf$ adj (disease$ or syndrome$ or disorder$)).ti,ab,kf.
117	spielmeyer vogt$.ti,ab,kf.
118	Xanthomatosis, Cerebrotendinous/
119	((cerebrotendineous or cerebrotendinous or cerebrotendious or cerebral) adj3 (xanthomatos$ or cholesteros$)).ti,ab,kf.
120	bogaert-scherer-epstein$.ti,ab,kf.
121	Wolman Disease/

122	(wolman$ adj (disease$ or syndrome$ or disorder$)).ti,ab,kf.
123	lysosomal acid lipase deficien$.ti,ab,kf.
124	exp Mucopolysaccharidoses/
125	mucopolysaccharidos$.ti,ab,kf.
126	(hurler$ adj2 (syndrome$ or disease$ or disorder$)).ti,ab,kf.
127	(hunter$ adj2 (syndrome$ or disease$ or disorder$)).ti,ab,kf.
128	(MPS1 or MPS2 or MPS3 or MPS4 or MPS5 or MPS6 or MPS7 or MPS-1 or MPS-2 or MPS-3 or MPS-4 or MPS-5 or MPS-6 or MPS-7 or MPSI or MPSII or MPSIII or MPSIV or MPSV or MPSVI or MPSVII or MPS-I or MPS-II or MPS-III or MPS-IV or MPS-V or MPS-VI or MPS-VII).ti,ab,kf.
129	(beta glucuronidase deficien$ or sly syndrome$ or sly disorder$ or sly disease$).ti,ab,kf.
130	(maroteaux-lamy$ or marotaeux-lamy$ or polydystrophic dwarfism).ti,ab,kf.
131	(morquio$ or moriquio$ or beta galactosidase deficien$).ti,ab,kf.
132	(sanfilippo$ or sanfillipo$).ti,ab,kf.
133	Mucolipidoses/
134	(mucolipidos$ or pseudo-hurler$ or pseudohurler$).ti,ab,kf.
135	((inclusion-cell or i-cell) adj (disease$ or syndrome$ or disorder$)).ti,ab,kf.
136	Fucosidosis/
137	(fucosidos$ or fucidos$).ti,ab,kf.
138	"Congenital Disorders of Glycosylation"/
139	((cdg or ctg) adj (disease$ or disorder$ or syndrome$)).ti,ab,kf.
140	(carbohydrate-deficient glycoprotein adj (disease$ or disorder$ or syndrome$)).ti,ab,kf.
141	(congenital disorder$ adj3 glycosylation).ti,ab,kf.
142	Lesch-Nyhan Syndrome/
143	juvenile gout.ti,ab,kf.
144	Menkes Kinky Hair Syndrome/
145	menkes$.ti,ab,kf.
146	((copper transport or steely hair or kinky hair) adj (disease$ or syndrome$ or disorder$)).ti,ab,kf.
147	alpha 1-Antitrypsin Deficiency/
148	(antitrypsin deficien$ or A1AD).ti,ab,kf.
149	(AAT deficien$ or alpha-1 protease deficien$).ti,ab,kf.
150	bisalbumin?emi$.ti,ab,kf.
151	Lipodystrophy, Congenital Generalized/
152	(congenital generali?ed lipodystroph$ or berardinelli$ or bernardnelli$).ti,ab,kf.
153	Landau-Kleffner Syndrome/
154	(landau-kleffner$ or infantile acquired aphasia$ or acquired epileptic aphasia$).ti,ab,kf.
155	(aphasia$ adj5 convulsive).ti,ab,kf.
156	Rett Syndrome/
157	(rett$ adj (syndrome$ or disease$ or disorder$)).ti,ab,kf.
158	cerebroatrophic hyperammon?emi$.ti,ab,kf.
159	Huntington Disease/
160	huntington$.ti,ab,kf.
161	exp Spinocerebellar Ataxias/
162	((nyhan$ or kelley-seegmiller$) adj (syndrome$ or disorder$ or disease$)).ti,ab,kf.

163	(spinocerebellar ataxia$ or ataxia$ telangiectasia$ or louis-bar$ syndrome$ or louis-bar$ disease$ or louis-bar$ disorder$ or machado-joseph$ or joseph$ disease$ or joseph$ disorder$ or joseph$ syndrome$).ti,ab,kf.
164	Friedreich Ataxia/
165	((friedreich$ or friedrich$) adj3 ataxia$).ti,ab,kf.
166	spinocerebellar degenerat$.ti,ab,kf.
167	"Spinal Muscular Atrophies of Childhood"/
168	(spinal muscular atroph$ or werdnig hoffman$).ti,ab,kf.
169	(dubowitz$ or kugelberg-welander$).ti,ab,kf.
170	Bulbar Palsy, Progressive/
171	(fazio-londe$ or faziolonde$ or progressive bulbar pals$).ti,ab,kf.
172	parkinson disease/ or parkinson disease, secondary/
173	(parkinson$ or hypokinetic rigid syndrome$ or hypokinetic rigid disease$ or hypokinetic rigid disorder$ or paralysis agitan$ or shaking pals$).ti,ab,kf.
174	Pantothenate Kinase-Associated Neurodegeneration/
175	(pantothenate kinase-associated neurodegenerat$ or PKAN or hallervorden-spatz$).ti,ab,kf.
176	((neurodegeneration adj3 brain iron accumulation) or NBIA$1).ti,ab,kf.
177	Olivopontocerebellar Atrophies/
178	(olivopontocerebellar atroph$ or OPCA or olivopontocerebellar degenerat$).ti,ab,kf.
179	(multiple system atrophy adj5 cerebellar).ti,ab,kf.
180	"Diffuse Cerebral Sclerosis of Schilder"/
181	(alper$ adj (disease$ or syndrome$ or disorder$)).ti,ab,kf.
182	(progressive sclerosing poliodystroph$ or progressive infantile poliodystroph$).ti,ab,kf.
183	(diffuse cerebral sclerosis adj5 schilder$).ti,ab,kf.
184	Leigh Disease/
185	(leigh$ adj (syndrome$ or disease$ or disorder$)).ti,ab,kf.
186	(subacute necrotizing encephalomyelopath$ or subacute necrotising encephalomyelopath$ or sub-acute necrotizing encephalomyelopath$ or sub-acute necrotising encephalomyelopath$ or SNEM).ti,ab,kf.
187	(aicardi-gouti?res or aicardia-gouti?res).ti,ab,kf.
188	(worster-drought$ or congenital suprabulbar pares$).ti,ab,kf.
189	multiple sclerosis/ or multiple sclerosis, chronic progressive/ or multiple sclerosis, relapsing-remitting/
190	(multiple sclerosis or disseminated sclerosis or encephalomyelitis disseminata$).ti,ab,kf.
191	(demyelinating adj (disease$ or syndrome$ or disorder$)).ti,ab,kf.
192	exp Epilepsies, Myoclonic/
193	myoclonic epileps$.ti,ab,kf.
194	((lafora$ or merrf$ or unverricht-lundborg$ or janz$) adj (disease$ or syndrome$ or disorder$)).ti,ab,kf.
195	lennox-gastaut$.ti,ab,kf.
196	(lennox$ adj (syndrome$ or disease$ or disorder$)).ti,ab,kf.
197	Spasms, Infantile/
198	(west$ adj (syndrome$ or disease$ or disorder$)).ti,ab,kf.
199	Epilepsia Partialis Continua/
200	(epilepsia partialis continua or kojevnikov$ or epilepsia partialis continuoa or kozhevnikof$).ti,ab,kf.

201	Charcot-Marie-Tooth Disease/
202	(charcot-marie-tooth$ or peroneal muscular atroph$).ti,ab,kf.
203	(progressive neuropathic muscular atroph$ or hereditary peroneal nerve dysfunction$ or peroneal neuropath$).ti,ab,kf.
204	"Hereditary Sensory and Motor Neuropathy"/
205	(hereditary sensory adj3 motor neuropath$).ti,ab,kf.
206	(hereditary motor adj3 sensory neuropath$).ti,ab,kf.
207	Refsum Disease, Infantile/
208	Peroxisomal Disorders/
209	(infantile refsum or infantile phytanic acid storage).ti,ab,kf.
210	Myasthenic Syndromes, Congenital/
211	congenital myasth?eni$.ti,ab,kf.
212	Muscular Dystrophy, Duchenne/
213	(duchenne muscular dystroph$ or dmd).ti,ab,kf.
214	exp Muscular Dystrophies, Limb-Girdle/
215	(limb-girdle or erb$ muscular dystroph$).ti,ab,kf.
216	(sarcoglycanopath$ or sarcoglycaopath$).ti,ab,kf.
217	Osteochondrodysplasias/
218	(osteochondrodysplas$ or schwartz-jampel or chondrodystrophi$ myotoni$ or myotoni$ chondrodystrophi$).ti,ab,kf.
219	Myotonia Congenita/
220	(congenita$ myotoni$ or myotoni$ congenita$).ti,ab,kf.
221	(thomsen$ adj (disease$ or disorder$ or syndrome$)).ti,ab,kf.
222	((recessive adj3 myotoni$) or becker$ myotoni$).ti,ab,kf.
223	Isaacs Syndrome/
224	(isaac$ adj (syndrome$ or disease$ or disorder$)).ti,ab,kf.
225	neuromyotoni$.ti,ab,kf.
226	Myotonic Disorders/
227	(paramyotoni$ congenita$ or congenita$ paramyotoni$).ti,ab,kf.
228	(eulenburg$ adj (disease$ or syndrome$ or disorder$)).ti,ab,kf.
229	(myotoni$ adj (disease$ or disorder$ or syndrome$)).ti,ab,kf.
230	pseudomyotoni$.ti,ab,kf.
231	exp Myopathies, Structural, Congenital/
232	(congenital adj3 myopath$).ti,ab,kf.
233	myopathycongenital.ti,ab,kf.
234	((nemaline or rod) adj3 myopath$).ti,ab,kf.
235	((central core or mini-core or minicore or multicore or multi-core) adj (disease$ or disorder$ or syndrome$ or myopath$)).ti,ab,kf.
236	fiber type disproportion.ti,ab,kf.
237	fibre type disproportion.ti,ab,kf.
238	Muscular Dystrophies/cn
239	(congenital$ adj5 muscular dystroph$).ti,ab,kf.
240	((centronuclear or myotubular) adj myopath$).ti,ab,kf.
241	exp Mitochondrial Myopathies/
242	(mitochondrial myopath$ or mitochondrial encephalomyopath$ or chronic progressive external ophthalmopleg$).ti,ab,kf.
243	((melas or kearns-sayre$) adj (syndrome$ or disease$ or disorder$)).ti,ab,kf.
244	Quadriplegia/ and spastic$.ti,ab,kf.
245	(spastic quadriplegi$ or spastic tetraplegi$).ti,ab,kf.
246	Reye Syndrome/

247	(reye$ adj (syndrome$ or disease$ or disorder$)).ti,ab,kf.
248	multiple pterygium.ti,ab,kf.
249	Hypertension, Pulmonary/ and primary$.ti,ab,kf.
250	((primary pulmonary or precapillary pulmonary or idiopathic pulmonary) adj (hypertension or ht or arterial hypertension)).ti,ab,kf.
251	((primary bronchopulmonary or precapillary bronchopulmonary or idiopathic bronchopulmonary) adj (hypertension or ht or arterial hypertension)).ti,ab,kf.
252	((primary lung or precapillary lung or idiopathic lung) adj (hypertension or ht or arterial hypertension)).ti,ab,kf.
253	ipah.ti,ab,kf.
254	Cardiomyopathy, Dilated/
255	((congestive or dilated) adj cardiomyopath$).ti,ab,kf.
256	exp Cardiomyopathy, Hypertrophic/
257	(hypertrophic adj cardiomyopath$).ti,ab,kf.
258	Cardiomyopathies/cn
259	(congenital adj3 cardiomyopath$).ti,ab,kf.
260	Cardiomyopathy, Restrictive/
261	(restrictive cardiomyopath$ or obliterative cardiomyopath$ or constrictive cardiomyopath$).ti,ab,kf.
262	exp Pulmonary Fibrosis/
263	(pulmonary fibros$ or lung fibros$ or bronchopulmonary fibros$ or fibrosing alveolit$ or interstitial pneumonit$).ti,ab,kf.
264	Respiratory Insufficiency/
265	(respiratory adj (failure$ or insufficienc$)).ti,ab,kf.
266	"Cystic Adenomatoid Malformation of Lung, Congenital"/
267	((cystic lung or cystic pulmonary or cystic bronchopulmonary) adj (disease$ or disorder or syndrome$)).ti,ab,kf.
268	(bronchogenic cyst$ or bronchopulmonary foregut malformation$).ti,ab,kf.
269	cystic adenomatoid malformation$.ti,ab,kf.
270	lobar emphysem$.ti,ab,kf.
271	(pulmonary sequestration$ or bronchopulmonary sequestration$ or lung sequestration$ or extralobar sequestration$ or extra-lobar sequestration$ or intralobar sequestration$ or intra-lobar sequestration$).ti,ab,kf.
272	pulmolithias$.ti,ab,kf.
273	exp Liver Failure/
274	((liver$1 or hepatic) adj3 fail$).ti,ab,kf.
275	exp Liver Cirrhosis/
276	(cirrhosis adj3 liver$1).ti,ab,kf.
277	Hepatic Veno-Occlusive Disease/
278	((veno-occlusive or venous occlusive) adj (disease$ or syndrome$ or disorder$)).ti,ab,kf.
279	Exocrine Pancreatic Insufficiency/
280	(swachman-diamond or shwachman-bodian or schwachmann-diamond or shwachmann-bodian).ti,ab,kf.
281	Wegener Granulomatosis/
282	wegener$ granulomatos$.ti,ab,kf.
283	(granulomatos$ adj3 polyangiit$).ti,ab,kf.
284	Osteolysis, Essential/
285	essential osteolys$.ti,ab,kf.

286	((gorham$ or gorham-stout$ or vanishing bone or phantom bone) adj (disease$ or syndrome$ or disorder)).ti,ab,kf.
287	((arc or arthrogryposis renal dysfunction cholestasis) adj (disease$ or syndrome$ or disorder)).ti,ab,kf.
288	Cerebral Hemorrhage/cn
289	Cerebral Hemorrhage, Traumatic/
290	Cerebral Hemorrhage/ and Birth Injuries/
291	(cerebral h?emorrhage$ and (birth$ adj3 injur$)).ti,ab,kf.
292	Asphyxia Neonatorum/
293	asphyxia neonatorum.ti,ab,kf.
294	((perinatal$ or neonatal$ or birth$) adj3 asphyxia$).ti,ab,kf.
295	Rubella Syndrome, Congenital/
296	congenital rubella.ti,ab,kf.
297	exp Cytomegalovirus Infections/cn
298	(congenital adj (cytomegalovirus$ or cmv)).ti,ab,kf.
299	Chickenpox/cn
300	exp Herpes Zoster/cn
301	Herpesvirus 3, Human/ and congenital$.ti,ab,kf.
302	((congenital or fetal or foetal) adj3 (varicella$ or chicken pox$ or VZV)).ti,ab,kf.
303	Toxoplasmosis, Congenital/
304	congenital toxoplasmos$.ti,ab,kf.
305	exp Hypoxia, Brain/
306	((brain$ or cerebral) adj3 hypoxi$).ti,ab,kf.
307	Renal Insufficiency/cn
308	Acute Kidney Injury/cn
309	Renal Insufficiency, Chronic/cn
310	Kidney Failure, Chronic/cn
311	(congenital$ adj3 (kidney failure$ or renal failure$ or kidney insufficienc$ or renal insufficienc$)).ti,ab,kf.
312	(congenital$ adj3 (kidney disease$ or renal disease$)).ti,ab,kf.
313	Anencephaly/
314	(anencephal$ or meroanencephal$ or craniorachischis$).ti,ab,kf.
315	(aprosencephal$ adj3 open cranium).ti,ab,kf.
316	Encephalocele/
317	(encephalocele$ or cranium bifidum).ti,ab,kf.
318	Dandy-Walker Syndrome/
319	dandy-walker$.ti,ab,kf.
320	Acrocallosal Syndrome/
321	(acrocallosal or acro-callosal or acrocolossal or acro colossal).ti,ab,kf.
322	Aicardi Syndrome/
323	(aicardi$ adj (syndrome$ or disease$ or disorder$)).ti,ab,kf.
324	Holoprosencephaly/
325	(holoprosencephal$ or arhinencephal$ or holosprosencephal$).ti,ab,kf.
326	Hydranencephaly/
327	(hydranencephal$ or hydrancephal$ or hydroanencephal$).ti,ab,kf.
328	exp Lissencephaly/
329	Microcephaly/
330	(lissencephal$ or walker-warburg$ or miller-dieker$ or norman-robert$ or microlissencephal$).ti,ab,kf.

331	((fukuyama$ or muscle-eye-brain) adj (syndrome$ or disease$ or disorder$)).ti,ab,kf.
332	"Malformations of Cortical Development"/
333	(microgyria$ or microgyrus or micro-gyria$ or micro-gyrus).ti,ab,kf.
334	(pachygyria$ or pachgyria$).ti,ab,kf.
335	agyria$.ti,ab,kf.
336	Septo-Optic Dysplasia/
337	((septo-optic or septooptic) adj dysplas$).ti,ab,kf.
338	de morsier$.ti,ab,kf.
339	(schizencephal$ or schizzencephal$).ti,ab,kf.
340	Arnold-Chiari Malformation/
341	chiari$ malformation$.ti,ab,kf.
342	Truncus Arteriosus, Persistent/
343	(truncus or common arterial trunk$).ti,ab,kf.
344	"Transposition of Great Vessels"/
345	((transposition$ or dextrotransposition$ or dtransposition$ or levotransposition$ or ltransposition$) adj3 (great arter$ or main arter$ or aorta$ or pulmonary arter$ or great vessel$ or main vessel$)).ti,ab,kf.
346	(dextro-tga or d-tga or levo-tga or l-tga).ti,ab,kf.
347	(double inlet adj3 ventricle$).ti,ab,kf.
348	DILV.ti,ab,kf.
349	single ventricle$.ti,ab,kf.
350	Heart Defects, Congenital/ and Atrial Appendage/
351	(isomerism adj3 atrial appendage$).ti,ab,kf.
352	(aspleni$ or polyspleni$ or poly-spleni$).ti,ab,kf.
353	"Tetralogy of Fallot"/
354	(tetralogy adj3 fallot$).ti,ab,kf.
355	Eisenmenger Complex/
356	(eisenmenger$ or tardive cyanos$ or eisenmeyer$).ti,ab,kf.
357	(pentalogy adj3 fallot$).ti,ab,kf.
358	Pulmonary Atresia/
359	((pulmonary or bronchopulmonary or lung$) adj3 atresia$).ti,ab,kf.
360	Tricuspid Atresia/
361	((tricuspid or tri) adj3 atresia$).ti,ab,kf.
362	Ebstein Anomaly/
363	(ebstein$ adj (anomal$ or malformation$)).ti,ab,kf.
364	Hypoplastic Left Heart Syndrome/
365	(hypoplastic left heart adj (syndrome$ or disease$ or disorder$)).ti,ab,kf.
366	((aortic or aorta$) adj3 atresia$).ti,ab,kf.
367	(mitral adj3 atresia$).ti,ab,kf.
368	((absence$ or absent$) adj3 (aorta$ or aortic)).ti,ab,kf.
369	(aplas$ adj3 (aorta$ or aortic)).ti,ab,kf.
370	exp Aortic Aneurysm/cn
371	(((aorta$ or aortic) adj3 aneurys$) and congenital$).ti,ab,kf.
372	(hypoplas$ adj3 (aorta$ or aortic)).ti,ab,kf.
373	(convulsion$ adj3 (aorta$ or aortic)).ti,ab,kf.
374	(persistent right adj3 (aorta$ or aortic)).ti,ab,kf.
375	((anomalous pulmonary venous or anamolous pulmonary venous) adj (connection or drainage or return)).ti,ab,kf.
376	((absence$ or absent$) adj3 vena$ cava$).ti,ab,kf.

377	(persistent left adj3 cardinal vein$).ti,ab,kf.
378	Scimitar Syndrome/
379	((scimitar$ or pulmonary venolobar) adj (syndrome$ or disease$ or disorder$)).ti,ab,kf.
380	(arteriovenous malformations/ or intracranial arteriovenous malformations/) and bilateral.ti,ab,kf.
381	((bilateral AV or bilateral arteriovenous or bilateral arterio-venous) adj3 malform$).ti,ab,kf.
382	((trachea$ or windpipe$ or wind-pipe$) adj3 atresia$).ti,ab,kf.
383	Tracheal Stenosis/
384	((trachea$ or laryngotrachea$ or glottic or subglottic or sub-glottic) adj3 stenosis).ti,ab,kf.
385	Bronchopulmonary Dysplasia/
386	((lung$ or pulmonary or bronchopulmonary) adj3 (hypoplas$ or dysplas$)).ti,ab,kf.
387	((absence$ or absent$) adj3 (esophag$ or oesophag$ or foodpipe or food-pipe$ or gullet$)).ti,ab,kf.
388	Intestinal Atresia/
389	(duoden$ adj3 atresia$).ti,ab,kf.
390	((absence$ or absent$) adj3 (intestin$ or gastrointestin$)).ti,ab,kf.
391	((intestin$ or gastrointestin$) adj3 atresia$).ti,ab,kf.
392	((intestin$ or gastrointestin$) adj3 stenos$).ti,ab,kf.
393	(cloaca$ adj3 (abnor$ or malform$ or anomal$)).ti,ab,kf.
394	(cloaca$ adj3 exopthalmo$).ti,ab,kf.
395	Biliary Atresia/
396	(biliary adj3 atresia$).ti,ab,kf.
397	(extrahepatic ductopen$ or extra-hepatic ductopen$ or progressive obliterative cholangiopath$).ti,ab,kf.
398	(biliary adj3 hypoplas$).ti,ab,kf.
399	(alagille$ adj3 atresia$).ti,ab,kf.
400	((absence$ or absent$) adj3 kidney$).ti,ab,kf.
401	(potter$ adj (sequence$ or syndrome$ or disease$ or disorder$)).ti,ab,kf.
402	Oligohydramnios/
403	oligohydramn$.ti,ab,kf.
404	Multicystic Dysplastic Kidney/
405	((kidney$ or renal) adj3 dysplas$).ti,ab,kf.
406	((meckel$ or meckelgruber$ or gruber$) adj (syndrome$ or disease$ or disorder$)).ti,ab,kf.
407	dysencephalia splanchnocystica$.ti,ab,kf.
408	(pena-shokeir$ or penn-shokeir$).ti,ab,kf.
409	(larsen$ adj (syndrome$ or disease$ or disorder$)).ti,ab,kf.
410	Acrocephalosyndactylia/
411	acrocephalosyndactyl$.ti,ab,kf.
412	(pfeiffer$ adj (syndrome$ or disease$ or syndrome$)).ti,ab,kf.
413	Short Rib-Polydactyly Syndrome/
414	short rib$1.ti,ab,kf.
415	(saldino-noonan$ or majewski$ or verma-naumoff$ or beemer-langer$).ti,ab,kf.
416	(jeune$ adj (syndrome$ or disease$ or disorder$)).ti,ab,kf.
417	asphyxiating thoracic dysplas$.ti,ab,kf.
418	exp Chondrodysplasia Punctata/

419	chondrodysplasia punctata$.ti,ab,kf.
420	((conradi$ or h?nermann$ or happle$) adj3 (syndrome$ or disease$ or disorder$)).ti,ab,kf.
421	Osteogenesis Imperfecta/
422	osteogenesis imperfecta.ti,ab,kf.
423	((brittle bone or lobstein$) adj (disease$ or disorder$ or syndrome$)).ti,ab,kf.
424	Osteochondrodysplasias/
425	(spondyloepimetaphyseal or spondyloepiphyseal or spendylo metaphyseal).ti,ab,kf.
426	Hernia, Umbilical/
427	(omphalocele$ or omphalocoele$ or exomphalos).ti,ab,kf.
428	(hernia$ adj3 umbilic$).ti,ab,kf.
429	Gastroschisis/
430	gastroschis$.ti,ab,kf.
431	Ichthyosis, Lamellar/
432	(lamellar$ adj3 ichthyos$).ti,ab,kf.
433	((harlequin$ or harloquin$) adj3 (ichthyos$ or baby or babies or f?etus$)).ti,ab,kf.
434	(ichthyosis congenita$ or ichthyosis fetalis or keratosis diffusa fetalis).ti,ab,kf.
435	exp Epidermolysis Bullosa/
436	epidermolysis bullosa$.ti,ab,kf.
437	(johanson-blizzard$ or johanna-blizzard$).ti,ab,kf.
438	Xeroderma Pigmentosum/
439	xeroderma pigmentosum.ti,ab,kf.
440	Ectodermal Dysplasia/
441	lacrimo-auriculo-dento-digital.ti,ab,kf.
442	ectodermal dysplas$.ti,ab,kf.
443	((ladd or eec) adj (syndrome$ or disease$ or disorder$)).ti,ab,kf.
444	Sturge-Weber Syndrome/
445	(sturge-weber or encephalotrigeminal angiomatos$).ti,ab,kf.
446	Fetal Alcohol Spectrum Disorders/
447	f?etal alcohol.ti,ab,kf.
448	Pierre Robin Syndrome/
449	pierre robin$.ti,ab,kf.
450	Acrocephalosyndactylia/
451	(acrocephalosyndact$ or acrocephalopolysyndact$).ti,ab,kf.
452	((apert$ or crouzon$ or saethre-chotzen$ or noack$ or carpenter$ or sakati-nyhan-tisdale$ or goodman$) adj (syndrome$ or disorder$ or disease$)).ti,ab,kf.
453	Fraser Syndrome/
454	(fraser$ adj (syndrome$ or disease$ or disorder$)).ti,ab,kf.
455	cryptophthalmos.ti,ab,kf.
456	(cyclopia$1 or cyclocephal$ or synophthalmi$).ti,ab,kf.
457	Goldenhar Syndrome/
458	(goldenhar$ or oculo-auriculo-vertebral).ti,ab,kf.
459	Mobius Syndrome/
460	((m?bius$ or moebius$) adj (syndrome$ or disease$ or disorder$)).ti,ab,kf.
461	Orofaciodigital Syndromes/
462	(orofaciodigital or oro-facial-digital or oral-facial-digital or papillon-league$ or psaume$).ti,ab,kf.
463	(robin$ adj (syndrome$ or disorder$ or disease$)).ti,ab,kf.

464	(freeman-sheldon$ or distal arthrogrypos$ or craniocarpotarsal dysplas$ or craniocarpotarsal dystroph$ or canio-carpo-tarsal or windmill-vane-hand$ or whistling-face).ti,ab,kf.
465	De Lange Syndrome/
466	((de lange$ or bushy$) adj (syndrome$ or disorder$ or disease$)).ti,ab,kf.
467	amsterdam dwarfism.ti,ab,kf.
468	(aarskog or faciodigitogenital or facio-digito-genital or facial digital genital or shawl scrotum or faciogenital or facio-genital).ti,ab,kf.
469	Cockayne Syndrome/
470	(cockayne$ or neill-dingwall$).ti,ab,kf.
471	(cerebro-oculo-facio-skeletal or cerebro-oculo-facial-skeletal).ti,ab,kf.
472	(dubowitz$ adj (syndrome$ or disease$ or disorder$)).ti,ab,kf.
473	(robinow$ or robinhow$).ti,ab,kf.
474	(f?etal face or f?etal facies or f?etal faces or acral dysostos$ or mesomelic dwarfism or covesdem$).ti,ab,kf.
475	Silver-Russell Syndrome/
476	(silver-russell$ or russell-silver$).ti,ab,kf.
477	(silver$ adj (syndrome$ or disease$ or disorder$)).ti,ab,kf.
478	((seckel$ or harper$) adj (syndrome$ or disease$ or disorder$)).ti,ab,kf.
479	(microcephalic primordial dwarfism or bird-headed dwarf$ or virchow-seckel dwarfism).ti,ab,kf.
480	Smith-Lemli-Opitz Syndrome/
481	(smith-lemli-opitz$ or dehydrocholesterol reductase deficien$).ti,ab,kf.
482	Prader-Willi Syndrome/
483	(prader-willi$ or pradar-willi$).ti,ab,kf.
484	Rubinstein-Taybi Syndrome/
485	(rubinstein-taybi$ or rubenstein-tabyii$ or broad thumb-hallux).ti,ab,kf.
486	((rubinstein$ or rubenstein$) adj2 (syndrome$ or disease$ or disorder$)).ti,ab,kf.
487	Nephritis, Hereditary/
488	(alport$ adj (syndrome$ or disease$ or disorder$)).ti,ab,kf.
489	(hereditary nephritis or h?emorrhagic familial nephritis).ti,ab,kf.
490	(hereditary deafness adj3 nephropath$).ti,ab,kf.
491	(h?ematuria adj3 nephropath$ adj3 deafness).ti,ab,kf.
492	Laurence-Moon Syndrome/
493	laurence-moon$.ti,ab,kf.
494	Bardet-Biedl Syndrome/
495	(bardet-biedl$ or biedl-bardet$).ti,ab,kf.
496	Zellweger Syndrome/
497	zellweger$.ti,ab,kf.
498	((cerebrohepatorenal or cerebro-hepato-renal) adj (syndrome$ or disease$ or disorder$)).ti,ab,kf.
499	(edward$ adj (syndrome$ or disease$ or disorder$)).ti,ab,kf.
500	"trisomy 18".ti,ab,kf.
501	(patau$ adj (syndrome$ or disease$ or disorder$)).ti,ab,kf.
502	("trisomy 13" or "trisomy D").ti,ab,kf.
503	"trisomy 22".ti,ab,kf.
504	"trisomy 9".ti,ab,kf.
505	"trisomy 10".ti,ab,kf.
506	duplication syndrome$.ti,ab,kf.
507	(("chromosome 8" or "chr 8") adj5 duplicat$).ti,ab,kf.

508	Chromosome Duplication/
509	exp X Chromosome/ab
510	exp X Chromosome/ and duplicat$.ti,ab,kf.
511	(("chromosome x" or "chr x") and duplicat$).ti,ab,kf.
512	(chromosom$ abnormality adj5 duplicat$).ti,ab,kf.
513	"tetrasomy 5p".ti,ab,kf.
514	(tetrasomy adj3 mosaic$).ti,ab,kf.
515	Chromosomes, Human, Pair 5/ and Mosaicism/
516	Tetrasomy/
517	Trisomy/ and (chromosomes, human, pair 9/ or chromosomes, human, pair 10/ or chromosomes, human, pair 13/ or Chromosomes, Human, Pair 18/ or chromosomes, human, pair 22/)
518	Chromosome Deletion/ and Chromosomes, Human, Pair 4/
519	(delet$ adj5 short arm adj5 "chrom$ 4").ti,ab,kf.
520	Wolf-Hirschhorn Syndrome/
521	((wolf-hirschhorn$ or wolff hirschorn$ or chromosome deletion dillan$ or pitt-rogers-dank$ or pitt$) adj3 (syndrome$ or disease$ or disorder$)).ti,ab,kf.
522	Cri-du-Chat Syndrome/
523	((cri du chat$ or crying cat$ or 5p or lejeune$) adj3 (syndrome$ or disease$ or disorder$)).ti,ab,kf.
524	Jacobsen Distal 11q Deletion Syndrome/
525	((jacobsen$ or 11q deletion) adj5 (syndrome$ or disease$ or disorder$)).ti,ab,kf.
526	exp Monosomy/ and Chromosomes, Human, Pair 9/
527	(9p minus or 9p deletion).ti,ab,kf.
528	(alfi$ adj (syndrome$ or disease$ or disorder$)).ti,ab,kf.
529	(degouchy$ or de gouchy$ or degrouchy$ or de grouchy$).ti,ab,kf.
530	distal 18q.ti,ab,kf.
531	Hypoventilation/cn
532	(ondine$ curse or congenital central hypoventilation or primary alveolar hypoventilation).ti,ab,kf.
533	Graft vs Host Disease/ and (Chronic Disease/ or chronic$.ti,ab,kf.)
534	(((graft vs host or graft versus host) adj (disease$ or syndrome$ or disorder)) and chronic$).ti,ab,kf.
535	or/1-534
536	Terminally Ill/
537	Terminal Care/
538	Palliative Care/
539	Hospices/ or Hospice Care/
540	(life adj2 limit$).ti,ab,kf.
541	(life adj2 threaten$).ti,ab,kf.
542	end of life.ti,ab,kf.
543	eol.ti,ab,kf.
544	(terminal$ adj2 (ill or illness$ or condition$1 or disease$1 or syndrome$ or disorder$)).ti,ab,kf.
545	(terminal adj2 (care$ or caring)).ti,ab,kf.
546	palliat$.ti,ab,kf.
547	(care adj2 dying).ti,ab,kf.
548	(technology adj2 dependent).ti,ab,kf.
549	hospice$.ti,ab,kf.
550	Rare Diseases/

551	Metabolic Diseases/
552	(severe adj2 (need or needs or illness$ or disease$1 or disabilit$ or impairment$1 or impediment$1 or condition$1 or disadvant$ or problem$1 or syndrome$1 or disorder$1)).ti,ab,kf.
553	(complex adj2 (need or needs or illness$ or disease$1 or disabilit$ or impairment$1 or impediment$1 or condition$1 or disadvant$ or problem$1 or syndrome$1 or disorder$1)).ti,ab,kf.
554	(rare adj2 (illness$ or disease$ or disabilit$ or impairment$ or impediment$ or condition$1 or syndrome$1 or disorder$1)).ti,ab,kf.
555	(multiple adj2 (need or needs or illness$ or disease$1 or disabilit$ or impairment$1 or impediment$ or condition$1 or disadvant$ or health or syndrome$1 or disorder$1)).ti,ab,kf.
556	(profound adj2 (need or needs or illness$ or disease$ or disabilit$ or impairment$ or impediment$ or condition$1 or syndrome$1 or disorder$1)).ti,ab,kf.
557	(intense adj2 (need or needs or illness$ or disease$ or disabilit$ or impairment$ or impediment$ or condition$1 or syndrome$1 or disorder$1)).ti,ab,kf.
558	(serious adj2 (disabilit$ or impairment$ or impediment$ or condition$1 or disadvant$)).ti,ab,kf.
559	or/536-558
560	exp HIV/
561	exp HIV Infections/
562	(HIV or human immunodeficiency virus$).ti,ab,kf.
563	(htlv or human t-lymphotropic virus$ or human t cell lymphotropic virus$).ti,ab,kf.
564	(acquired immune deficiency syndrome$ or acquired immunodeficiency syndrome$).ti,ab,kf.
565	(AIDS adj3 (virus$ or infection$)).ti,ab,kf.
566	(AIDS adj (related or associated)).ti,ab,kf.
567	exp Neoplasms/
568	(cancer$ or carcin$ or tumor$ or tumour$ or neoplas$ or adenocarcin$ or oncol$ or malignan$).ti,ab,kf.
569	Cystic Fibrosis/
570	(cystic fibrosis or fibrocystic or fibro-cystic or mucoviscidosis or cf).ti,ab,kf.
571	Cerebral Palsy/
572	(cerebr$ adj3 pals$).ti,ab,kf.
573	Muscle Spasticity/
574	spasticit$.ti,ab,kf.
575	Quadriplegia/
576	(spastic$ and (quadripleg$ or tetrapleg$)).ti,ab,kf.
577	exp Renal Insufficiency/
578	((kidney$ or renal) adj3 (failure$ or insufficienc$)).ti,ab,kf.
579	(end stage adj3 (kidney or renal)).ti,ab,kf.
580	(("stage 5" or "stage V") adj3 (kidney or renal)).ti,ab,kf.
581	(ESRD or ESKD or ESRF or ESKF or CRF or CKF).ti,ab,kf.
582	or/560-581
583	535 or 559 or 582
584	((Young adj1 people$) or Youth$ or Care leaver$ or residential child$ or Adolescen$ or Young adult$ or Young person$ or Young men$ or Young women$ or Teen$ or juvenile$ or Younger people or Youngster$ or Looked after or Child

	welfare or paediatric$ or pediatric$ or peadiatric$ or Young male$ or Young female$ or juvenile or children$ or child or childhood or (young adj1 patient$) or young carer$ or minors or puber$ or pubescen$ or ((secondary or high*) adj2 (school* or education))).ti,ab.
585	exp infant/
586	Child/
587	586 not 585
588	Disabled children/
589	exp Young Adult/
590	Adolescent, Hospitalized/
591	Adolescent, Institutionalized/
592	Child, Institutionalized/
593	Child, Hospitalized/
594	exp Adolescent/
595	or/584,587-594
596	((transition$ or transfer$ or handoff or handover or hand over) and (Service$ or care or clinic$ or healthcare or hospital$ or center$ or centre$ or facility or facilities or unit$ or department$ or institution$ or agency or agencies or hospice$ or provider$ or program$ or Coordinat$ or Framework$ or Managing or Managed or preparedness or Planning or Preparing or Preparation$ or Plan$ or Protocol$ or planned or Support or Supporting or Trajectory or Trajectories or Pathway$ or Process or Processes or Readiness or Partnership$ or programme$ or program$ or training or strateg$ or Failure$ or Barrier$ or system?)).ti.
597	((transition$ or transfer$ or handoff or handover or hand over) adj3 (Service$ or care or clinic$ or healthcare or hospital$ or center$ or centre$ or facility or facilities or unit$ or department$ or institution$ or agency or agencies or hospice$ or provider$ or program$ or Coordinat$ or Framework$ or Managing or Managed or preparedness or Planning or Preparing or Preparation$ or Plan$ or Protocol$ or planned or Support or Supporting or Trajectory or Trajectories or Pathway$ or Process or Processes or Readiness or Partnership$ or programme$ or program$ or training or strateg$ or Failure$ or Barrier$ or system?)).ab.
598	(continu$ and (care or healthcare or Support or Supporting or Failure$ or Barrier$)).ti.
599	(continu$ adj3 (care or healthcare or Support or Supporting or Failure$ or Barrier$)).ab.
600	transition to adult care/
601	continuity of patient care/
602	patient handoff/
603	Patient Care Planning/
604	Patient transfer/
605	(transition$ or transfer$ or handoff or handover or hand$ over).ti,ab.
606	(601 or 603) and 605
607	or/596-600,602,604,606
608	583 and 595 and 607
609	(letter or editorial or comment or news).pt.
610	exp animals/ not humans/
611	608 not (609 or 610)
612	limit 611 to (english language and yr="1990 -Current")

Embase (Ovid)

Concepts:

4. LLC: lines 1-584
5. Child/young adult: lines 585-596
6. Transition: lines 597-608

1	Creutzfeldt-Jakob disease/
2	(creutzfeldt-jakob$ or jakob-creutzfeldt$ or cjd or spongiform encephalopath$).ti,ab,kf.
3	Subacute Sclerosing Panencephalitis/
4	(subacute sclerosing panencephalit$ or sub-acute sclerosing panencephalit$ or sspe or subacute sclerosing leukoencephalit$ or sub-acute sclerosing leukoencephalit$ or van bogaert$ leukoencephalit$ or measles inclusion body encephalit$ or mibe).ti,ab,kf.
5	beta Thalassemia/
6	(beta adj (thalass?emi$ or thalas?emi$)).ti,ab,kf.
7	((thalass?emi$ or thalas?emi$) adj major).ti,ab,kf.
8	exp Aplastic Anemia/
9	((hypoplastic or aplastic) adj an?emi$).ti,ab,kf.
10	(medullary adj3 hypoplas$).ti,ab,kf.
11	exp Neutropenia/
12	((severe or chronic$) adj3 neutropeni$).ti,ab,kf.
13	immune deficiency/ or acquired immune deficiency syndrome/
14	(immun$ deficiency adj (syndrome$ or disease$ or disorder$)).ti,ab,kf.
15	(immunodeficiency adj (syndrome$ or disease$ or disorder$)).ti,ab,kf.
16	DiGeorge Syndrome/
17	(digeorge$ or di george$ or sedlackova$ or opitz g-bbb or velocardiofacial or velo-cardiofacial or velo-cardio-facial or shprintzen$ or ctaf).ti,ab,kf.
18	((deletion or vcf or pharyngeal pouch or thymic aplasia or anomaly face) adj (syndrome$ or disease$ or disorder$)).ti,ab,kf.
19	Common Variable Immunodeficiency/
20	((common variable or late onset) adj3 (immunodeficienc$ or immune deficienc$ or immunoglobulin deficienc$ or hypogammaglobulin$)).ti,ab,kf.
21	acquired hypogammaglobulin$.ti,ab,kf.
22	Cryoglobulinemia/
23	cryoglobulin?em$.ti,ab,kf.
24	Polyendocrinopathy/
25	((autoimmune or failure$) adj3 (polyglandular$ or polyendocrin$)).ti,ab,kf.
26	Progeria/
27	(progeria or hutchinson-gilford$).ti,ab,kf.
28	Tyrosinemias/
29	tyrosin?em$.ti,ab,kf.
30	Maple Syrup Urine Disease/
31	(maple syrup urine or msud).ti,ab,kf.
32	branched chain.ti,ab,kf.
33	(bckd adj5 (deficienc$ or ketoacid$ or keto-acid$)).ti,ab,kf.
34	hyperleucine-isoleucin$.ti,ab,kf.
35	Methylmalonic Acid/

36	(methylmalonic acid?emi$ or methylmalonic aciduri$ or methyl malonic acid?emi$ or methyl malonic aciduri$).ti,ab,kf.
37	Propionic Acidemia/
38	(propionic acid?em$ or propionic acidur$ or propionyl-CoA carboxylase deficienc$ or ketotic glycin?em$).ti,ab,kf.
39	Adrenoleukodystrophy/
40	(adrenoleukodystroph$ or x-ald or schilder-addison$ or addison-schilder$ or adrenomyeloneuropath$).ti,ab,kf.
41	Carnitine Palmitoyltransferase/
42	((carnitine palmityltransferase or carnitine palmitoyltransferase or carnitine o-palmityltransferase or carnitine o-palmitoyltransferase) adj3 deficienc$).ti,ab,kf.
43	Fanconi renotubular Syndrome/
44	(fanconi$ adj (syndrome$ or disease$ or disorder$)).ti,ab,kf.
45	(ocular adj3 (renal or kidney)).ti,ab,kf.
46	Cystinosis/
47	(cystinos$ or cystine storage or cystine diathes$ or cystine disease$).ti,ab,kf.
48	Lowe Syndrome/
49	((lowe or lowes or oculocerebrorenal or cerebrooculorenal or cerebro-oculo-renal) adj3 (syndrome$ or disease$ or disorder$)).ti,ab,kf.
50	Metalloprotein/ and deficien$.mp.
51	Molybdenum/ and deficien$.mp.
52	(molybdenum cofactor deficien$ or molybdenum co-factor deficien$).ti,ab,kf.
53	Oxidoreductases Acting on Sulfur Group Donors/ and Deficien$.mp.
54	Sulfite Oxidase/ and deficien$.mp.
55	((sulphite$ or sulfite$) adj3 oxidase deficien$).ti,ab,kf.
56	Argininosuccinic Acid/
57	(argininosuccinic acidur$ or argininosuccinic acid?emi$).ti,ab,kf.
58	Citrullinemia/
59	(citrullin?emi$ or citrullinuri$).ti,ab,kf.
60	"disorders of amino acid and protein metabolism"/
61	(glutaric acid?emi$ or glutaric aciduri$).ti,ab,kf.
62	Hyperglycinemia/
63	(glycine encephalopath$ or non-ketotic hyperglycin?emi$ or nonketotic hyperglycin?emi$).ti,ab,kf.
64	Hyperargininemia/
65	(arginin?emi$ or arginase deficien$ or hyperarginin?emi$).ti,ab,kf.
66	Aminoaciduria/
67	(aminoaciduri$ or aminoacid?emi$).ti,ab,kf.
68	exp glycogen storage disease/
69	(glycogen storage adj (disease$ or syndrome$ or disorder$)).ti,ab,kf.
70	(pompe$ adj (disease$ or syndrome$ or disorder$)).ti,ab,kf.
71	Galactosemia/
72	galactos?emi$.ti,ab,kf.
73	Pyruvate Dehydrogenase Complex Deficiency/
74	(pyruvate dehydrogenase adj3 deficien$).ti,ab,kf.
75	(oxalosis and (renal or kidney$)).ti,ab,kf.
76	exp Gangliosidosis/
77	gangliosidos$.ti,ab,kf.
78	(sandhoff$ adj (disease$ or syndrome$ or disorder$)).ti,ab,kf.
79	tay sach$.ti,ab,kf.

80	Mucolipidosis/
81	mucolipidos$.ti,ab,kf.
82	Canavan Disease/
83	(canavan$ leucodystroph$ or aspartoacylase deficien$ or aminoacylase 2 deficien$).ti,ab,kf.
84	((canavan$ or canavan-van bogaert-bertrand$) adj (disease$ or syndrome$ or disorder$)).ti,ab,kf.
85	Gaucher Disease/
86	(gaucher$ adj (disease$ or syndrome$ or disorder$)).ti,ab,kf.
87	(glucocerebrosidase deficien$ or glucosylceramidase deficien$).ti,ab,kf.
88	Metachromatic Leukodystrophy/
89	(metachromatic leukodystroph$ or arylsulfatase A deficien$ or metachromic leukodystroph$).ti,ab,kf.
90	exp Niemann Pick Disease/
91	(niemann-pick$ or sphingomyelinase deficien$).ti,ab,kf.
92	Sphingolipidosis/
93	sphingolipidos$.ti,ab,kf.
94	Fabry Disease/
95	(fabry$ adj (disease$ or syndrome$ or disorder$)).ti,ab,kf.
96	(angiokeratoma corporis diffusum or alpha-galactosidase A deficien$).ti,ab,kf.
97	Globoid Cell Leukodystrophy/
98	(krabbe$ adj (disease$ or syndrome$ or disorder$)).ti,ab,kf.
99	(globoid cell leukodystroph$ or galactosylceramide lipidos$ or galactosylcerebrosidase deficien$ or galactosylceramidase deficien$).ti,ab,kf.
100	Farber disease/
101	(farber$ adj (disease$ or syndrome$ or disorder$)).ti,ab,kf.
102	(farber$ lipogranulomatos$ or ceramidase deficien$ or fibrocytic dysmucopolysaccharidos$).ti,ab,kf.
103	Pelizaeus Merzbacher Disease/
104	pelizaeus-merzbacher$.ti,ab,kf.
105	Sulfatase/ and deficien$.mp.
106	Multiple Sulfatase Deficiency/
107	(sulfatase deficien$ or sulphatase deficien$ or mucosulfatidos$).ti,ab,kf.
108	(austin$ adj (disease$ or syndrome$ or disorder$)).ti,ab,kf.
109	Metachromatic Leukodystrophy/
110	sulfatidos$.ti,ab,kf.
111	histiocytosis/
112	sea-blue histiocyt$.ti,ab,kf.
113	Neuronal Ceroid-Lipofuscinosis/
114	(batten$ adj (disease$ or syndrome$ or disorder$)).ti,ab,kf.
115	(neuronal ceroid lipofuscinos$ or santavuori-haltia$ or jansky-bielschowsky$ or bielschowsky-jansky$).ti,ab,kf.
116	(kuf$ adj (disease$ or syndrome$ or disorder$)).ti,ab,kf.
117	spielmeyer vogt$.ti,ab,kf.
118	Cerebrotendinous Xanthomatosis/
119	((cerebrotendineous or cerebrotendinous or cerebrotendious or cerebral) adj3 (xanthomatos$ or cholesteros$)).ti,ab,kf.
120	bogaert-scherer-epstein$.ti,ab,kf.
121	Wolman Disease/
122	(wolman$ adj (disease$ or syndrome$ or disorder$)).ti,ab,kf.

123	lysosomal acid lipase deficien$.ti,ab,kf.
124	exp Mucopolysaccharidosis/
125	mucopolysaccharidos$.ti,ab,kf.
126	(hurler$ adj2 (syndrome$ or disease$ or disorder$)).ti,ab,kf.
127	(hunter$ adj2 (syndrome$ or disease$ or disorder$)).ti,ab,kf.
128	(MPS1 or MPS2 or MPS3 or MPS4 or MPS5 or MPS6 or MPS7 or MPS-1 or MPS-2 or MPS-3 or MPS-4 or MPS-5 or MPS-6 or MPS-7 or MPSI or MPSII or MPSIII or MPSIV or MPSV or MPSVI or MPSVII or MPS-I or MPS-II or MPS-III or MPS-IV or MPS-V or MPS-VI or MPS-VII).ti,ab,kf.
129	(beta glucuronidase deficien$ or sly syndrome$ or sly disorder$ or sly disease$).ti,ab,kf.
130	(maroteaux-lamy$ or marotaeux-lamy$ or polydystrophic dwarfism).ti,ab,kf.
131	(morquio$ or moriquio$ or beta galactosidase deficien$).ti,ab,kf.
132	(sanfilippo$ or sanfillipo$).ti,ab,kf.
133	Mucolipidosis/
134	(mucolipidos$ or pseudo-hurler$ or pseudohurler$).ti,ab,kf.
135	((inclusion-cell or i-cell) adj (disease$ or syndrome$ or disorder$)).ti,ab,kf.
136	Fucosidosis/
137	(fucosidos$ or fucidos$).ti,ab,kf.
138	Congenital Disorder of Glycosylation/
139	((cdg or ctg) adj (disease$ or disorder$ or syndrome$)).ti,ab,kf.
140	(carbohydrate-deficient glycoprotein adj (disease$ or disorder$ or syndrome$)).ti,ab,kf.
141	(congenital disorder$ adj3 glycosylation).ti,ab,kf.
142	Lesch Nyhan Syndrome/
143	((nyhan$ or kelley-seegmiller$) adj (syndrome$ or disorder$ or disease$)).ti,ab,kf.
144	juvenile gout.ti,ab,kf.
145	Menkes Syndrome/
146	menkes$.ti,ab,kf.
147	((copper transport or steely hair or kinky hair) adj (disease$ or syndrome$ or disorder$)).ti,ab,kf.
148	alpha 1 Antitrypsin Deficiency/
149	(antitrypsin deficien$ or A1AD).ti,ab,kf.
150	(AAT deficien$ or alpha-1 protease deficien$).ti,ab,kf.
151	bisalbumin?emi$.ti,ab,kf.
152	Congenital Generalized Lipodystrophy/
153	(congenital generali?ed lipodystroph$ or berardinelli$ or bernardnelli$).ti,ab,kf.
154	Landau Kleffner Syndrome/
155	(landau-kleffner$ or infantile acquired aphasia$ or acquired epileptic aphasia$).ti,ab,kf.
156	(aphasia$ adj5 convulsive).ti,ab,kf.
157	Rett Syndrome/
158	(rett$ adj (syndrome$ or disease$ or disorder$)).ti,ab,kf.
159	cerebroatrophic hyperammon?emi$.ti,ab,kf.
160	Huntington chorea/
161	huntington$.ti,ab,kf.
162	exp spinocerebellar degeneration/
163	((nyhan$ or kelley-seegmiller$) adj (syndrome$ or disorder$ or disease$)).ti,ab,kf.
164	(spinocerebellar ataxia$ or ataxia$ telangiectasia$ or louis-bar$ syndrome$ or louis-bar$ disease$ or louis-bar$ disorder$ or machado-joseph$ or joseph$ disease$ or joseph$ disorder$ or joseph$ syndrome$).ti,ab,kf.
165	Friedreich Ataxia/
166	((friedreich$ or friedrich$) adj3 ataxia$).ti,ab,kf.

167	spinocerebellar degenerat$.ti,ab,kf.
168	hereditary spinal muscular atrophy/
169	(spinal muscular atroph$ or werdnig hoffman$).ti,ab,kf.
170	(dubowitz$ or kugelberg-welander$).ti,ab,kf.
171	Bulbar paralysis/
172	(fazio-londe$ or faziolonde$ or progressive bulbar pals$).ti,ab,kf.
173	parkinson disease/
174	(parkinson$ or hypokinetic rigid syndrome$ or hypokinetic rigid disease$ or hypokinetic rigid disorder$ or paralysis agitan$ or shaking pals$).ti,ab,kf.
175	neurodegeneration with brain iron accumulation/
176	(pantothenate kinase-associated neurodegenerat$ or PKAN or hallervorden-spatz$).ti,ab,kf.
177	((neurodegeneration adj3 brain iron accumulation) or NBIA$1).ti,ab,kf.
178	olivopontocerebellar atrophy/
179	(olivopontocerebellar atroph$ or OPCA or olivopontocerebellar degenerat$).ti,ab,kf.
180	(multiple system atrophy adj5 cerebellar).ti,ab,kf.
181	Schilder disease/
182	(alper$ adj (disease$ or syndrome$ or disorder$)).ti,ab,kf.
183	(progressive sclerosing poliodystroph$ or progressive infantile poliodystroph$).ti,ab,kf.
184	(diffuse cerebral sclerosis adj5 schilder$).ti,ab,kf.
185	Leigh Disease/
186	(leigh$ adj (syndrome$ or disease$ or disorder$)).ti,ab,kf.
187	(subacute necrotizing encephalomyelopath$ or subacute necrotising encephalomyelopath$ or sub-acute necrotizing encephalomyelopath$ or sub-acute necrotising encephalomyelopath$ or SNEM).ti,ab,kf.
188	(aicardi-gouti?res or aicardia-gouti?res).ti,ab,kf.
189	(worster-drought$ or congenital suprabulbar pares$).ti,ab,kf.
190	multiple sclerosis/
191	(multiple sclerosis or disseminated sclerosis or encephalomyelitis disseminata$).ti,ab,kf.
192	(demyelinating adj (disease$ or syndrome$ or disorder$)).ti,ab,kf.
193	exp myoclonus epilepsy/
194	myoclonic epileps$.ti,ab,kf.
195	((lafora$ or merrf$ or unverricht-lundborg$ or janz$) adj (disease$ or syndrome$ or disorder$)).ti,ab,kf.
196	lennox-gastaut$.ti,ab,kf.
197	(lennox$ adj (syndrome$ or disease$ or disorder$)).ti,ab,kf.
198	infantile spasm/
199	(west$ adj (syndrome$ or disease$ or disorder$)).ti,ab,kf.
200	epileptic state/
201	(epilepsia partialis continua or kojevnikov$ or epilepsia partialis continuoa or kozhevnikof$).ti,ab,kf.
202	hereditary motor sensory neuropathy/
203	(charcot-marie-tooth$ or peroneal muscular atroph$).ti,ab,kf.
204	(progressive neuropathic muscular atroph$ or hereditary peroneal nerve dysfunction$ or peroneal neuropath$).ti,ab,kf.
205	hereditary motor sensory neuropathy/
206	(hereditary sensory adj3 motor neuropath$).ti,ab,kf.
207	(hereditary motor adj3 sensory neuropath$).ti,ab,kf.
208	infantile Refsum disease/
209	disorders of peroxisomal functions/

210	(infantile refsum or infantile phytanic acid storage).ti,ab,kf.
211	congenital myasthenic syndrome/
212	congenital myasth?eni$.ti,ab,kf.
213	Duchenne muscular dystrophy/
214	(duchenne muscular dystroph$ or dmd).ti,ab,kf.
215	exp limb girdle muscular dystrophy/
216	(limb-girdle or erb$ muscular dystroph$).ti,ab,kf.
217	(sarcoglycanopath$ or sarcoglycaopath$).ti,ab,kf.
218	chondrodysplasia/
219	(osteochondrodysplas$ or schwartz-jampel or chondrodystrophi$ myotoni$ or myotoni$ chondrodystrophi$).ti,ab,kf.
220	Thomsen disease/
221	(congenita$ myotoni$ or myotoni$ congenita$).ti,ab,kf.
222	(thomsen$ adj (disease$ or disorder$ or syndrome$)).ti,ab,kf.
223	((recessive adj3 myotoni$) or becker$ myotoni$).ti,ab,kf.
224	myokymia/
225	(isaac$ adj (syndrome$ or disease$ or disorder$)).ti,ab,kf.
226	neuromyotoni$.ti,ab,kf.
227	myotonia/
228	(paramyotoni$ congenita$ or congenita$ paramyotoni$).ti,ab,kf.
229	(eulenburg$ adj (disease$ or syndrome$ or disorder$)).ti,ab,kf.
230	(myotoni$ adj (disease$ or disorder$ or syndrome$)).ti,ab,kf.
231	pseudomyotoni$.ti,ab,kf.
232	exp myopathy/ and congen$.mp.
233	(congenital adj3 myopath$).ti,ab,kf.
234	myopathycongenital.ti,ab,kf.
235	((nemaline or rod) adj3 myopath$).ti,ab,kf.
236	((central core or mini-core or minicore or multicore or multi-core) adj (disease$ or disorder$ or syndrome$ or myopath$)).ti,ab,kf.
237	fiber type disproportion.ti,ab,kf.
238	fibre type disproportion.ti,ab,kf.
239	Muscular Dystrophies/cn [Congenital]
240	(congenital$ adj5 muscular dystroph$).ti,ab,kf.
241	((centronuclear or myotubular) adj myopath$).ti,ab,kf.
242	exp mitochondrial myopathy/
243	(mitochondrial myopath$ or mitochondrial encephalomyopath$ or chronic progressive external ophthalmopleg$).ti,ab,kf.
244	((melas or kearns-sayre$) adj (syndrome$ or disease$ or disorder$)).ti,ab,kf.
245	Quadriplegia/ and spastic$.ti,ab,kf.
246	(spastic quadriplegi$ or spastic tetraplegi$).ti,ab,kf.
247	Reye Syndrome/
248	(reye$ adj (syndrome$ or disease$ or disorder$)).ti,ab,kf.
249	multiple pterygium.ti,ab,kf.
250	pulmonary hypertension/ and primary$.ti,ab,kf.
251	((primary pulmonary or precapillary pulmonary or idiopathic pulmonary) adj (hypertension or ht or arterial hypertension)).ti,ab,kf.
252	((primary bronchopulmonary or precapillary bronchopulmonary or idiopathic bronchopulmonary) adj (hypertension or ht or arterial hypertension)).ti,ab,kf.
253	((primary lung or precapillary lung or idiopathic lung) adj (hypertension or ht or arterial hypertension)).ti,ab,kf.

254	ipah.ti,ab,kf.
255	congestive cardiomyopathy/
256	((congestive or dilated) adj cardiomyopath$).ti,ab,kf.
257	exp hypertrophic cardiomyopathy/
258	(hypertrophic adj cardiomyopath$).ti,ab,kf.
259	Cardiomyopathies/cn [Congenital]
260	(congenital adj3 cardiomyopath$).ti,ab,kf.
261	restrictive cardiomyopathy/
262	(restrictive cardiomyopath$ or obliterative cardiomyopath$ or constrictive cardiomyopath$).ti,ab,kf.
263	exp lung fibrosis/
264	(pulmonary fibros$ or lung fibros$ or bronchopulmonary fibros$ or fibrosing alveolit$ or interstitial pneumonit$).ti,ab,kf.
265	respiratory failure/
266	(respiratory adj (failure$ or insufficienc$)).ti,ab,kf.
267	cystic adenomatoid malformation/
268	((cystic lung or cystic pulmonary or cystic bronchopulmonary) adj (disease$ or disorder or syndrome$)).ti,ab,kf.
269	(bronchogenic cyst$ or bronchopulmonary foregut malformation$).ti,ab,kf.
270	cystic adenomatoid malformation$.ti,ab,kf.
271	lobar emphysem$.ti,ab,kf.
272	(pulmonary sequestration$ or bronchopulmonary sequestration$ or lung sequestration$ or extralobar sequestration$ or extra-lobar sequestration$ or intralobar sequestration$ or intra-lobar sequestration$).ti,ab,kf.
273	pulmolithias$.ti,ab,kf.
274	exp Liver Failure/
275	((liver$1 or hepatic) adj3 fail$).ti,ab,kf.
276	exp Liver Cirrhosis/
277	(cirrhosis adj3 liver$1).ti,ab,kf.
278	liver vein obstruction/
279	((veno-occlusive or venous occlusive) adj (disease$ or syndrome$ or disorder$)).ti,ab,kf.
280	Exocrine Pancreatic Insufficiency/
281	(swachman-diamond or shwachman-bodian or schwachmann-diamond or shwachmann-bodian).ti,ab,kf.
282	Wegener Granulomatosis/
283	wegener$ granulomatos$.ti,ab,kf.
284	(granulomatos$ adj3 polyangiit$).ti,ab,kf.
285	Osteolysis/ and essential.mp.
286	essential osteolys$.ti,ab,kf.
287	((gorham$ or gorham-stout$ or vanishing bone or phantom bone) adj (disease$ or syndrome$ or disorder)).ti,ab,kf.
288	((arc or arthrogryposis renal dysfunction cholestasis) adj (disease$ or syndrome$ or disorder)).ti,ab,kf.
289	brain hemorrhage/ and Congen$.mp.
290	brain hemorrhage/ and Traum$.mp.
291	brain hemorrhage/ and Birth Injury/
292	(cerebral h?emorrhage$ and (birth$ adj3 injur$)).ti,ab,kf.
293	newborn hypoxia/
294	asphyxia neonatorum.ti,ab,kf.
295	((perinatal$ or neonatal$ or birth$) adj3 asphyxia$).ti,ab,kf.

296	congenital rubella syndrome/
297	congenital rubella.ti,ab,kf.
298	exp Cytomegalovirus Infection/ and congen$.mp.
299	(congenital adj (cytomegalovirus$ or cmv)).ti,ab,kf.
300	(Chickenpox and Congen$).mp. [mp=title, abstract, heading word, drug trade name, original title, device manufacturer, drug manufacturer, device trade name, keyword, floating subheading word, candidate term word]
301	exp Herpes Zoster/ and Congen$.mp.
302	Herpesvirus 3, Human/ and congenital$.ti,ab,kf.
303	((congenital or fetal or foetal) adj3 (varicella$ or chicken pox$ or VZV)).ti,ab,kf.
304	congenital toxoplasmosis/
305	congenital toxoplasmos$.ti,ab,kf.
306	exp brain hypoxia/
307	((brain$ or cerebral) adj3 hypoxi$).ti,ab,kf.
308	kidney failure/ and Congen$.mp.
309	acute kidney failure/ and Congen$.mp.
310	chronic kidney failure/ and Congen$.mp.
311	chronic kidney failure/ and Congen$.mp.
312	(congenital$ adj3 (kidney failure$ or renal failure$ or kidney insufficienc$ or renal insufficienc$)).ti,ab,kf.
313	(congenital$ adj3 (kidney disease$ or renal disease$)).ti,ab,kf.
314	anencephalus/
315	(anencephal$ or meroanencephal$ or craniorachischis$).ti,ab,kf.
316	(aprosencephal$ adj3 open cranium).ti,ab,kf.
317	Encephalocele/
318	(encephalocele$ or cranium bifidum).ti,ab,kf.
319	Dandy Walker Syndrome/
320	dandy-walker$.ti,ab,kf.
321	Acrocallosal Syndrome/
322	(acrocallosal or acro-callosal or acrocolossal or acro colossal).ti,ab,kf.
323	Aicardi Syndrome/
324	(aicardi$ adj (syndrome$ or disease$ or disorder$)).ti,ab,kf.
325	Holoprosencephaly/
326	(holoprosencephal$ or arhinencephal$ or holosprosencephal$).ti,ab,kf.
327	Hydranencephaly/
328	(hydranencephal$ or hydrancephal$ or hydroanencephal$).ti,ab,kf.
329	exp agyria/
330	Microcephaly/
331	(lissencephal$ or walker-warburg$ or miller-dieker$ or norman-robert$ or microlissencephal$).ti,ab,kf.
332	((fukuyama$ or muscle-eye-brain) adj (syndrome$ or disease$ or disorder$)).ti,ab,kf.
333	cortical dysplasia/
334	(microgyria$ or microgyrus or micro-gyria$ or micro-gyrus).ti,ab,kf.
335	(pachygyria$ or pachgyria$).ti,ab,kf.
336	agyria$.ti,ab,kf.
337	Septooptic Dysplasia/
338	((septo-optic or septooptic) adj dysplas$).ti,ab,kf.
339	de morsier$.ti,ab,kf.
340	(schizencephal$ or schizzencephal$).ti,ab,kf.
341	Arnold Chiari Malformation/

342	chiari$ malformation$.ti,ab,kf.
343	Persistent Truncus Arteriosus/
344	(truncus or common arterial trunk$).ti,ab,kf.
345	great vessels transposition/
346	((transposition$ or dextrotransposition$ or dtransposition$ or levotransposition$ or ltransposition$) adj3 (great arter$ or main arter$ or aorta$ or pulmonary arter$ or great vessel$ or main vessel$)).ti,ab,kf.
347	(dextro-tga or d-tga or levo-tga or l-tga).ti,ab,kf.
348	(double inlet adj3 ventricle$).ti,ab,kf.
349	DILV.ti,ab,kf.
350	single ventricle$.ti,ab,kf.
351	congenital heart malformation/ and heart atrium appendage/
352	(isomerism adj3 atrial appendage$).ti,ab,kf.
353	(aspleni$ or polyspleni$ or poly-spleni$).ti,ab,kf.
354	Fallot tetralogy/
355	(tetralogy adj3 fallot$).ti,ab,kf.
356	Eisenmenger Complex/
357	(eisenmenger$ or tardive cyanos$ or eisenmeyer$).ti,ab,kf.
358	(pentalogy adj3 fallot$).ti,ab,kf.
359	pulmonary valve atresia/
360	((pulmonary or bronchopulmonary or lung$) adj3 atresia$).ti,ab,kf.
361	tricuspid valve atresia/
362	((tricuspid or tri) adj3 atresia$).ti,ab,kf.
363	Ebstein Anomaly/
364	(ebstein$ adj (anomal$ or malformation$)).ti,ab,kf.
365	Hypoplastic Left Heart Syndrome/
366	(hypoplastic left heart adj (syndrome$ or disease$ or disorder$)).ti,ab,kf.
367	((aortic or aorta$) adj3 atresia$).ti,ab,kf.
368	(mitral adj3 atresia$).ti,ab,kf.
369	((absence$ or absent$) adj3 (aorta$ or aortic)).ti,ab,kf.
370	(aplas$ adj3 (aorta$ or aortic)).ti,ab,kf.
371	exp Aortic Aneurysm/cn [Congenital]
372	(((aorta$ or aortic) adj3 aneurys$) and congenital$).ti,ab,kf.
373	(hypoplas$ adj3 (aorta$ or aortic)).ti,ab,kf.
374	(convulsion$ adj3 (aorta$ or aortic)).ti,ab,kf.
375	(persistent right adj3 (aorta$ or aortic)).ti,ab,kf.
376	((anomalous pulmonary venous or anamolous pulmonary venous) adj (connection or drainage or return)).ti,ab,kf.
377	((absence$ or absent$) adj3 vena$ cava$).ti,ab,kf.
378	(persistent left adj3 cardinal vein$).ti,ab,kf.
379	Scimitar Syndrome/
380	((scimitar$ or pulmonary venolobar) adj (syndrome$ or disease$ or disorder$)).ti,ab,kf.
381	(arteriovenous malformations/ or intracranial arteriovenous malformations/) and bilateral.ti,ab,kf.
382	((bilateral AV or bilateral arteriovenous or bilateral arterio-venous) adj3 malform$).ti,ab,kf.
383	((trachea$ or windpipe$ or wind-pipe$) adj3 atresia$).ti,ab,kf.
384	Tracheal Stenosis/
385	((trachea$ or laryngotrachea$ or glottic or subglottic or sub-glottic) adj3 stenosis).ti,ab,kf.

386	lung dysplasia/
387	((lung$ or pulmonary or bronchopulmonary) adj3 (hypoplas$ or dysplas$)).ti,ab,kf.
388	((absence$ or absent$) adj3 (esophag$ or oesophag$ or foodpipe or food-pipe$ or gullet$)).ti,ab,kf.
389	intestine atresia/
390	(duoden$ adj3 atresia$).ti,ab,kf.
391	((absence$ or absent$) adj3 (intestin$ or gastrointestin$)).ti,ab,kf.
392	((intestin$ or gastrointestin$) adj3 atresia$).ti,ab,kf.
393	((intestin$ or gastrointestin$) adj3 stenos$).ti,ab,kf.
394	(cloaca$ adj3 (abnor$ or malform$ or anomal$)).ti,ab,kf.
395	(cloaca$ adj3 exopthalmo$).ti,ab,kf.
396	bile duct atresia/
397	(biliary adj3 atresia$).ti,ab,kf.
398	(extrahepatic ductopen$ or extra-hepatic ductopen$ or progressive obliterative cholangiopath$).ti,ab,kf.
399	(biliary adj3 hypoplas$).ti,ab,kf.
400	(alagille$ adj3 atresia$).ti,ab,kf.
401	((absence$ or absent$) adj3 kidney$).ti,ab,kf.
402	(potter$ adj (sequence$ or syndrome$ or disease$ or disorder$)).ti,ab,kf.
403	Oligohydramnios/
404	oligohydramn$.ti,ab,kf.
405	Multicystic Dysplastic Kidney/
406	((kidney$ or renal) adj3 dysplas$).ti,ab,kf.
407	((meckel$ or meckelgruber$ or gruber$) adj (syndrome$ or disease$ or disorder$)).ti,ab,kf.
408	dysencephalia splanchnocystica$.ti,ab,kf.
409	(pena-shokeir$ or penn-shokeir$).ti,ab,kf.
410	(larsen$ adj (syndrome$ or disease$ or disorder$)).ti,ab,kf.
411	acrocephalosyndactyly/
412	acrocephalosyndactyl$.ti,ab,kf.
413	(pfeiffer$ adj (syndrome$ or disease$ or syndrome$)).ti,ab,kf.
414	chondrodysplasia/
415	short rib$1.ti,ab,kf.
416	(saldino-noonan$ or majewski$ or verma-naumoff$ or beemer-langer$).ti,ab,kf.
417	(jeune$ adj (syndrome$ or disease$ or disorder$)).ti,ab,kf.
418	asphyxiating thoracic dysplas$.ti,ab,kf.
419	exp chondrodysplasia punctata/
420	chondrodysplasia punctata$.ti,ab,kf.
421	((conradi$ or h?nermann$ or happle$) adj3 (syndrome$ or disease$ or disorder$)).ti,ab,kf.
422	Osteogenesis Imperfecta/
423	osteogenesis imperfecta.ti,ab,kf.
424	((brittle bone or lobstein$) adj (disease$ or disorder$ or syndrome$)).ti,ab,kf.
425	chondrodysplasia/
426	(spondyloepimetaphyseal or spondyloepiphyseal or spendylo metaphyseal).ti,ab,kf.
427	umbilical hernia/
428	(omphalocele$ or omphalocoele$ or exomphalos).ti,ab,kf.
429	(hernia$ adj3 umbilic$).ti,ab,kf.
430	Gastroschisis/
431	gastroschis$.ti,ab,kf.

432	lamellar ichthyosis/
433	(lamellar$ adj3 ichthyos$).ti,ab,kf.
434	((harlequin$ or harloquin$) adj3 (ichthyos$ or baby or babies or f?etus$)).ti,ab,kf.
435	(ichthyosis congenita$ or ichthyosis fetalis or keratosis diffusa fetalis).ti,ab,kf.
436	exp Epidermolysis Bullosa/
437	epidermolysis bullosa$.ti,ab,kf.
438	(johanson-blizzard$ or johanna-blizzard$).ti,ab,kf.
439	Xeroderma Pigmentosum/
440	xeroderma pigmentosum.ti,ab,kf.
441	Ectodermal Dysplasia/
442	lacrimo-auriculo-dento-digital.ti,ab,kf.
443	ectodermal dysplas$.ti,ab,kf.
444	((ladd or eec) adj (syndrome$ or disease$ or disorder$)).ti,ab,kf.
445	Sturge Weber Syndrome/
446	(sturge-weber or encephalotrigeminal angiomatos$).ti,ab,kf.
447	fetal alcohol syndrome/
448	f?etal alcohol.ti,ab,kf.
449	Pierre Robin Syndrome/
450	pierre robin$.ti,ab,kf.
451	acrocephalosyndactyly/
452	(acrocephalosyndact$ or acrocephalopolysyndact$).ti,ab,kf.
453	((apert$ or crouzon$ or saethre-chotzen$ or noack$ or carpenter$ or sakati-nyhan-tisdale$ or goodman$) adj (syndrome$ or disorder$ or disease$)).ti,ab,kf.
454	Fraser Syndrome/
455	(fraser$ adj (syndrome$ or disease$ or disorder$)).ti,ab,kf.
456	cryptophthalmos.ti,ab,kf.
457	(cyclopia$1 or cyclocephal$ or synophthalmi$).ti,ab,kf.
458	Goldenhar Syndrome/
459	(goldenhar$ or oculo-auriculo-vertebral).ti,ab,kf.
460	Mobius Syndrome/
461	((m?bius$ or moebius$) adj (syndrome$ or disease$ or disorder$)).ti,ab,kf.
462	dysostosis/
463	(orofaciodigital or oro-facial-digital or oral-facial-digital or papillon-league$ or psaume$).ti,ab,kf.
464	(robin$ adj (syndrome$ or disorder$ or disease$)).ti,ab,kf.
465	(freeman-sheldon$ or distal arthrogrypos$ or craniocarpotarsal dysplas$ or craniocarpotarsal dystroph$ or canio-carpo-tarsal or windmill-vane-hand$ or whistling-face).ti,ab,kf.
466	De Lange Syndrome/
467	((de lange$ or bushy$) adj (syndrome$ or disorder$ or disease$)).ti,ab,kf.
468	amsterdam dwarfism.ti,ab,kf.
469	(aarskog or faciodigitogenital or facio-digito-genital or facial digital genital or shawl scrotum or faciogenital or facio-genital).ti,ab,kf.
470	Cockayne Syndrome/
471	(cockayne$ or neill-dingwall$).ti,ab,kf.
472	(cerebro-oculo-facio-skeletal or cerebro-oculo-facial-skeletal).ti,ab,kf.
473	(dubowitz$ adj (syndrome$ or disease$ or disorder$)).ti,ab,kf.
474	(robinow$ or robinhow$).ti,ab,kf.
475	(f?etal face or f?etal facies or f?etal faces or acral dysostos$ or mesomelic dwarfism or covesdem$).ti,ab,kf.

476	Silver Russell Syndrome/
477	(silver-russell$ or russell-silver$).ti,ab,kf.
478	(silver$ adj (syndrome$ or disease$ or disorder$)).ti,ab,kf.
479	((seckel$ or harper$) adj (syndrome$ or disease$ or disorder$)).ti,ab,kf.
480	(microcephalic primordial dwarfism or bird-headed dwarf$ or virchow-seckel dwarfism).ti,ab,kf.
481	Smith Lemli Opitz Syndrome/
482	(smith-lemli-opitz$ or dehydrocholesterol reductase deficien$).ti,ab,kf.
483	Prader Willi Syndrome/
484	(prader-willi$ or pradar-willi$).ti,ab,kf.
485	Rubinstein syndrome/
486	(rubinstein-taybi$ or rubenstein-tabyii$ or broad thumb-hallux).ti,ab,kf.
487	((rubinstein$ or rubenstein$) adj2 (syndrome$ or disease$ or disorder$)).ti,ab,kf.
488	nephritis/ and (hered$ or inherit$).mp.
489	(alport$ adj (syndrome$ or disease$ or disorder$)).ti,ab,kf.
490	(hereditary nephritis or h?emorrhagic familial nephritis).ti,ab,kf.
491	(hereditary deafness adj3 nephropath$).ti,ab,kf.
492	(h?ematuria adj3 nephropath$ adj3 deafness).ti,ab,kf.
493	Laurence Moon Syndrome/
494	laurence-moon$.ti,ab,kf.
495	Bardet Biedl Syndrome/
496	(bardet-biedl$ or biedl-bardet$).ti,ab,kf.
497	Zellweger Syndrome/
498	zellweger$.ti,ab,kf.
499	((cerebrohepatorenal or cerebro-hepato-renal) adj (syndrome$ or disease$ or disorder$)).ti,ab,kf.
500	(edward$ adj (syndrome$ or disease$ or disorder$)).ti,ab,kf.
501	"trisomy 18".ti,ab,kf.
502	(patau$ adj (syndrome$ or disease$ or disorder$)).ti,ab,kf.
503	("trisomy 13" or "trisomy D").ti,ab,kf.
504	"trisomy 22".ti,ab,kf.
505	"trisomy 9".ti,ab,kf.
506	"trisomy 10".ti,ab,kf.
507	duplication syndrome$.ti,ab,kf.
508	(("chromosome 8" or "chr 8") adj5 duplicat$).ti,ab,kf.
509	Chromosome Duplication/
510	exp X Chromosome/ and abnorm$.mp.
511	exp X Chromosome/ and duplicat$.ti,ab,kf.
512	(("chromosome x" or "chr x") and duplicat$).ti,ab,kf.
513	(chromosom$ abnormality adj5 duplicat$).ti,ab,kf.
514	"tetrasomy 5p".ti,ab,kf.
515	(tetrasomy adj3 mosaic$).ti,ab,kf.
516	Chromosome 5/ and Mosaicism/
517	Tetrasomy/
518	Trisomy/ and (chromosome 9/ or chromosome 10/ or chromosome 13/ or Chromosome 18/ or chromosome 22/)
519	Chromosome Deletion/ and Chromosome 4/
520	(delet$ adj5 short arm adj5 "chrom$ 4").ti,ab,kf.
521	Wolf Hirschhorn Syndrome/

522	((wolf-hirschhorn$ or wolff hirschorn$ or chromosome deletion dillan$ or pitt-rogers-dank$ or pitt$) adj3 (syndrome$ or disease$ or disorder$)).ti,ab,kf.
523	cat cry Syndrome/
524	((cri du chat$ or crying cat$ or 5p or lejeune$) adj3 (syndrome$ or disease$ or disorder$)).ti,ab,kf.
525	Jacobsen syndrome/
526	((jacobsen$ or 11q deletion) adj5 (syndrome$ or disease$ or disorder$)).ti,ab,kf.
527	exp Monosomy/ and Chromosome 9/
528	(9p minus or 9p deletion).ti,ab,kf.
529	(alfi$ adj (syndrome$ or disease$ or disorder$)).ti,ab,kf.
530	(degouchy$ or de gouchy$ or degrouchy$ or de grouchy$).ti,ab,kf.
531	distal 18q.ti,ab,kf.
532	congenital central hypoventilation syndrome/
533	(ondine$ curse or congenital central hypoventilation or primary alveolar hypoventilation).ti,ab,kf.
534	Graft versus host reaction/ and (Chronic Disease/ or chronic$.ti,ab,kf.)
535	(((graft vs host or graft versus host) adj (disease$ or syndrome$ or disorder)) and chronic$).ti,ab,kf.
536	or/1-534
537	terminally ill patient/
538	Terminal Care/
539	palliative therapy/
540	Hospice/ or Hospice Care/
541	(life adj2 limit$).ti,ab,kf.
542	(life adj2 threaten$).ti,ab,kf.
543	end of life.ti,ab,kf.
544	eol.ti,ab,kf.
545	(terminal$ adj2 (ill or illness$ or condition$1 or disease$1 or syndrome$ or disorder$)).ti,ab,kf.
546	(terminal adj2 (care$ or caring)).ti,ab,kf.
547	palliat$.ti,ab,kf.
548	(care adj2 dying).ti,ab,kf.
549	(technology adj2 dependent).ti,ab,kf.
550	hospice$.ti,ab,kf.
551	Rare Disease/
552	metabolic disorder/
553	(severe adj2 (need or needs or illness$ or disease$1 or disabilit$ or impairment$1 or impediment$1 or condition$1 or disadvant$ or problem$1 or syndrome$1 or disorder$1)).ti,ab,kf.
554	(complex adj2 (need or needs or illness$ or disease$1 or disabilit$ or impairment$1 or impediment$1 or condition$1 or disadvant$ or problem$1 or syndrome$1 or disorder$1)).ti,ab,kf.
555	(rare adj2 (illness$ or disease$ or disabilit$ or impairment$ or impediment$ or condition$1 or syndrome$1 or disorder$1)).ti,ab,kf.
556	(multiple adj2 (need or needs or illness$ or disease$1 or disabilit$ or impairment$1 or impediment$ or condition$1 or disadvant$ or health or syndrome$1 or disorder$1)).ti,ab,kf.
557	(profound adj2 (need or needs or illness$ or disease$ or disabilit$ or impairment$ or impediment$ or condition$1 or syndrome$1 or disorder$1)).ti,ab,kf.

558	(intense adj2 (need or needs or illness$ or disease$ or disabilit$ or impairment$ or impediment$ or condition$1 or syndrome$1 or disorder$1)).ti,ab,kf.
559	(serious adj2 (disabilit$ or impairment$ or impediment$ or condition$1 or disadvant$)).ti,ab,kf.
560	or/537-559
561	exp Human immunodeficiency virus/
562	exp Human immunodeficiency virus infection/
563	(HIV or human immunodeficiency virus$).ti,ab,kf.
564	(htlv or human t-lymphotropic virus$ or human t cell lymphotropic virus$).ti,ab,kf.
565	(acquired immune deficiency syndrome$ or acquired immunodeficiency syndrome$).ti,ab,kf.
566	(AIDS adj3 (virus$ or infection$)).ti,ab,kf.
567	(AIDS adj (related or associated)).ti,ab,kf.
568	exp Neoplasm/
569	(cancer$ or carcin$ or tumor$ or tumour$ or neoplas$ or adenocarcin$ or oncol$ or malignan$).ti,ab,kf.
570	Cystic Fibrosis/
571	(cystic fibrosis or fibrocystic or fibro-cystic or mucoviscidosis or cf).ti,ab,kf.
572	Cerebral Palsy/
573	(cerebr$ adj3 pals$).ti,ab,kf.
574	spasticity/
575	spasticit$.ti,ab,kf.
576	Quadriplegia/
577	(spastic$ and (quadripleg$ or tetrapleg$)).ti,ab,kf.
578	exp kidney failure/
579	((kidney$ or renal) adj3 (failure$ or insufficienc$)).ti,ab,kf.
580	(end stage adj3 (kidney or renal)).ti,ab,kf.
581	(("stage 5" or "stage V") adj3 (kidney or renal)).ti,ab,kf.
582	(ESRD or ESKD or ESRF or ESKF or CRF or CKF).ti,ab,kf.
583	or/561-582
584	536 or 560 or 583
585	((Young adj1 people$) or Youth$ or Care leaver$ or residential child$ or Adolescen$ or Young adult$ or Young person$ or Young men$ or Young women$ or Teen$ or juvenile$ or Younger people or Youngster$ or Looked after or Child welfare or paediatric$ or pediatric$ or peadiatric$ or Young male$ or Young female$ or juvenile or children$ or child or childhood or (young adj1 patient$) or young carer$ or minors or puber$ or pubescen$ or ((secondary or high*) adj2 (school* or education))).ti,ab.
586	exp infant/
587	Child/
588	587 not 586
589	handicapped child/
590	exp Young Adult/
591	hospitalized adolescent/
592	institutionalized adolescent/
593	institutionalized child/
594	hospitalized child/
595	exp Adolescent/
596	or/585,588-595
597	((transition$ or transfer$ or handoff or handover or hand over) and (Service$ or care or clinic$ or healthcare or hospital$ or center$ or centre$ or facility or facilities or unit$ or

	department$ or institution$ or agency or agencies or hospice$ or provider$ or program$ or Coordinat$ or Framework$ or Managing or Managed or preparedness or Planning or Preparing or Preparation$ or Plan$ or Protocol$ or planned or Support or Supporting or Trajectory or Trajectories or Pathway$ or Process or Processes or Readiness or Partnership$ or programme$ or program$ or training or strateg$ or Failure$ or Barrier$ or system?)).ti.
598	((transition$ or transfer$ or handoff or handover or hand over) adj3 (Service$ or care or clinic$ or healthcare or hospital$ or center$ or centre$ or facility or facilities or unit$ or department$ or institution$ or agency or agencies or hospice$ or provider$ or program$ or Coordinat$ or Framework$ or Managing or Managed or preparedness or Planning or Preparing or Preparation$ or Plan$ or Protocol$ or planned or Support or Supporting or Trajectory or Trajectories or Pathway$ or Process or Processes or Readiness or Partnership$ or programme$ or program$ or training or strateg$ or Failure$ or Barrier$ or system?)).ab.
599	(continu$ and (care or healthcare or Support or Supporting or Failure$ or Barrier$)).ti.
600	(continu$ adj3 (care or healthcare or Support or Supporting or Failure$ or Barrier$)).ab.
601	transition to adult care/
602	patient care/
603	clinical handover/
604	Patient Care Planning/
605	patient transport/
606	(transition$ or transfer$ or handoff or handover or hand$ over).ti,ab.
607	(602 or 604) and 606
608	or/597-601,605,607
609	584 and 596 and 608
610	(letter or editorial or comment or news).pt.
611	exp animal/ not human/
612	609 not (610 or 611)
613	limit 612 to (english language and yr="1990 -Current")

Psychinfo (Ovid)

Concepts:

1. LLC: lines 1-401
2. Child/young adult: lines 402-404
3. Transition: lines 405-415

1	Creutzfeldt Jakob Syndrome/
2	(creutzfeldt-jakob$ or jakob-creutzfeldt$ or cjd or spongiform encephalopath$).ti,ab,kf.
3	(subacute sclerosing panencephalit$ or sub-acute sclerosing panencephalit$ or sspe or subacute sclerosing leukoencephalit$ or sub-acute sclerosing leukoencephalit$ or van bogaert$ leukoencephalit$ or measles inclusion body encephalit$ or mibe).ti,ab,kf.
4	(beta adj (thalass?emi$ or thalas?emi$)).ti,ab,kf.
5	((thalass?emi$ or thalas?emi$) adj major).ti,ab,kf.
6	((hypoplastic or aplastic) adj an?emi$).ti,ab,kf.
7	(medullary adj3 hypoplas$).ti,ab,kf.
8	((severe or chronic$) adj3 neutropeni$).ti,ab,kf.
9	immunologic deficiency syndromes/ or acquired immunodeficiency syndrome/
10	(immun$ deficiency adj (syndrome$ or disease$ or disorder$)).ti,ab,kf.
11	(immunodeficiency adj (syndrome$ or disease$ or disorder$)).ti,ab,kf.
12	(digeorge$ or di george$ or sedlackova$ or opitz g-bbb or velocardiofacial or velo-cardiofacial or velo-cardio-facial or shprintzen$ or ctaf).ti,ab,kf.
13	((deletion or vcf or pharyngeal pouch or thymic aplasia or anomaly face) adj (syndrome$ or disease$ or disorder$)).ti,ab,kf.
14	((common variable or late onset) adj3 (immunodeficienc$ or immune deficienc$ or immunoglobulin deficienc$ or hypogammaglobulin$)).ti,ab,kf.
15	acquired hypogammaglobulin$.ti,ab,kf.
16	cryoglobulin?em$.ti,ab,kf.
17	((autoimmune or failure$) adj3 (polyglandular$ or polyendocrin$)).ti,ab,kf.
18	(progeria or hutchinson-gilford$).ti,ab,kf.
19	tyrosin?em$.ti,ab,kf.
20	(maple syrup urine or msud).ti,ab,kf.
21	branched chain.ti,ab,kf.
22	(bckd adj5 (deficienc$ or ketoacid$ or keto-acid$)).ti,ab,kf.
23	hyperleucine-isoleucin$.ti,ab,kf.
24	(methylmalonic acid?emi$ or methylmalonic aciduri$ or methyl malonic acid?emi$ or methyl malonic aciduri$).ti,ab,kf.
25	(propionic acid?em$ or propionic acidur$ or propionyl-CoA carboxylase deficienc$ or ketotic glycin?em$).ti,ab,kf.
26	(adrenoleukodystroph$ or x-ald or schilder-addison$ or addison-schilder$ or adrenomyeloneuropath$).ti,ab,kf.
27	((carnitine palmityltransferase or carnitine palmitoyltransferase or carnitine o-palmityltransferase or carnitine o-palmitoyltransferase) adj3 deficienc$).ti,ab,kf.

28	(fanconi$ adj (syndrome$ or disease$ or disorder$)).ti,ab,kf.
29	(ocular adj3 (renal or kidney)).ti,ab,kf.
30	(cystinos$ or cystine storage or cystine diathes$ or cystine disease$).ti,ab,kf.
31	((lowe or lowes or oculocerebrorenal or cerebrooculorenal or cerebro-oculo-renal) adj3 (syndrome$ or disease$ or disorder$)).ti,ab,kf.
32	(molybdenum cofactor deficien$ or molybdenum co-factor deficien$).ti,ab,kf.
33	((sulphite$ or sulfite$) adj3 oxidase deficien$).ti,ab,kf.
34	(argininosuccinic acidur$ or argininosuccinic acid?emi$).ti,ab,kf.
35	(citrullin?emi$ or citrullinuri$).ti,ab,kf.
36	(glutaric acid?emi$ or glutaric aciduri$).ti,ab,kf.
37	(glycine encephalopath$ or non-ketotic hyperglycin?emi$ or nonketotic hyperglycin?emi$).ti,ab,kf.
38	(arginin?emi$ or arginase deficien$ or hyperarginin?emi$).ti,ab,kf.
39	(aminoaciduri$ or aminoacid?emi$).ti,ab,kf.
40	(glycogen storage adj (disease$ or syndrome$ or disorder$)).ti,ab,kf.
41	(pompe$ adj (disease$ or syndrome$ or disorder$)).ti,ab,kf.
42	galactos?emi$.ti,ab,kf.
43	(pyruvate dehydrogenase adj3 deficien$).ti,ab,kf.
44	(oxalosis and (renal or kidney$)).ti,ab,kf.
45	gangliosidos$.ti,ab,kf.
46	(sandhoff$ adj (disease$ or syndrome$ or disorder$)).ti,ab,kf.
47	tay sach$.ti,ab,kf.
48	mucolipidos$.ti,ab,kf.
49	(canavan$ leucodystroph$ or aspartoacylase deficien$ or aminoacylase 2 deficien$).ti,ab,kf.
50	((canavan$ or canavan-van bogaert-bertrand$) adj (disease$ or syndrome$ or disorder$)).ti,ab,kf.
51	(gaucher$ adj (disease$ or syndrome$ or disorder$)).ti,ab,kf.
52	(glucocerebrosidase deficien$ or glucosylceramidase deficien$).ti,ab,kf.
53	(metachromatic leukodystroph$ or arylsulfatase A deficien$ or metachromic leukodystroph$).ti,ab,kf.
54	(niemann-pick$ or sphingomyelinase deficien$).ti,ab,kf.
55	sphingolipidos$.ti,ab,kf.
56	(fabry$ adj (disease$ or syndrome$ or disorder$)).ti,ab,kf.
57	(angiokeratoma corporis diffusum or alpha-galactosidase A deficien$).ti,ab,kf.
58	(krabbe$ adj (disease$ or syndrome$ or disorder$)).ti,ab,kf.
59	(globoid cell leukodystroph$ or galactosylceramide lipidos$ or galactosylcerebrosidase deficien$ or galactosylceramidase deficien$).ti,ab,kf.
60	(farber$ adj (disease$ or syndrome$ or disorder$)).ti,ab,kf.
61	(farber$ lipogranulomatos$ or ceramidase deficien$ or fibrocytic dysmucopolysaccharidos$).ti,ab,kf.
62	pelizaeus-merzbacher$.ti,ab,kf.
63	(sulfatase deficien$ or sulphatase deficien$ or mucosulfatidos$).ti,ab,kf.
64	(austin$ adj (disease$ or syndrome$ or disorder$)).ti,ab,kf.
65	sulfatidos$.ti,ab,kf.
66	sea-blue histiocyt$.ti,ab,kf.

67	(batten$ adj (disease$ or syndrome$ or disorder$)).ti,ab,kf.
68	(neuronal ceroid lipofuscinos$ or santavuori-haltia$ or jansky-bielschowsky$ or bielschowsky-jansky$).ti,ab,kf.
69	(kuf$ adj (disease$ or syndrome$ or disorder$)).ti,ab,kf.
70	spielmeyer vogt$.ti,ab,kf.
71	((cerebrotendineous or cerebrotendinous or cerebrotendious or cerebral) adj3 (xanthomatos$ or cholesteros$)).ti,ab,kf.
72	bogaert-scherer-epstein$.ti,ab,kf.
73	(wolman$ adj (disease$ or syndrome$ or disorder$)).ti,ab,kf.
74	lysosomal acid lipase deficien$.ti,ab,kf.
75	mucopolysaccharidos$.ti,ab,kf.
76	(hurler$ adj2 (syndrome$ or disease$ or disorder$)).ti,ab,kf.
77	(hunter$ adj2 (syndrome$ or disease$ or disorder$)).ti,ab,kf.
78	(MPS1 or MPS2 or MPS3 or MPS4 or MPS5 or MPS6 or MPS7 or MPS-1 or MPS-2 or MPS-3 or MPS-4 or MPS-5 or MPS-6 or MPS-7 or MPSI or MPSII or MPSIII or MPSIV or MPSV or MPSVI or MPSVII or MPS-I or MPS-II or MPS-III or MPS-IV or MPS-V or MPS-VI or MPS-VII).ti,ab,kf.
79	(beta glucuronidase deficien$ or sly syndrome$ or sly disorder$ or sly disease$).ti,ab,kf.
80	(maroteaux-lamy$ or marotaeux-lamy$ or polydystrophic dwarfism).ti,ab,kf.
81	(morquio$ or moriquio$ or beta galactosidase deficien$).ti,ab,kf.
82	(sanfilippo$ or sanfillipo$).ti,ab,kf.
83	(mucolipidos$ or pseudo-hurler$ or pseudohurler$).ti,ab,kf.
84	((inclusion-cell or i-cell) adj (disease$ or syndrome$ or disorder$)).ti,ab,kf.
85	(fucosidos$ or fucidos$).ti,ab,kf.
86	((cdg or ctg) adj (disease$ or disorder$ or syndrome$)).ti,ab,kf.
87	(carbohydrate-deficient glycoprotein adj (disease$ or disorder$ or syndrome$)).ti,ab,kf.
88	(congenital disorder$ adj3 glycosylation).ti,ab,kf.
89	juvenile gout.ti,ab,kf.
90	menkes$.ti,ab,kf.
91	((copper transport or steely hair or kinky hair) adj (disease$ or syndrome$ or disorder$)).ti,ab,kf.
92	(antitrypsin deficien$ or A1AD).ti,ab,kf.
93	(AAT deficien$ or alpha-1 protease deficien$).ti,ab,kf.
94	bisalbumin?emi$.ti,ab,kf.
95	(congenital generali?ed lipodystroph$ or berardinelli$ or bernardnelli$).ti,ab,kf.
96	(landau-kleffner$ or infantile acquired aphasia$ or acquired epileptic aphasia$).ti,ab,kf.
97	(aphasia$ adj5 convulsive).ti,ab,kf.
98	Rett Syndrome/
99	(rett$ adj (syndrome$ or disease$ or disorder$)).ti,ab,kf.
100	cerebroatrophic hyperammon?emi$.ti,ab,kf.
101	Huntingtons Disease/
102	huntington$.ti,ab,kf.
103	((nyhan$ or kelley-seegmiller$) adj (syndrome$ or disorder$ or disease$)).ti,ab,kf.

104	(spinocerebellar ataxia$ or ataxia$ telangiectasia$ or louis-bar$ syndrome$ or louis-bar$ disease$ or louis-bar$ disorder$ or machado-joseph$ or joseph$ disease$ or joseph$ disorder$ or joseph$ syndrome$).ti,ab,kf.
105	((friedreich$ or friedrich$) adj3 ataxia$).ti,ab,kf.
106	spinocerebellar degenerat$.ti,ab,kf.
107	(spinal muscular atroph$ or werdnig hoffman$).ti,ab,kf.
108	(dubowitz$ or kugelberg-welander$).ti,ab,kf.
109	(fazio-londe$ or faziolonde$ or progressive bulbar pals$).ti,ab,kf.
110	parkinsons disease/
111	(parkinson$ or hypokinetic rigid syndrome$ or hypokinetic rigid disease$ or hypokinetic rigid disorder$ or paralysis agitan$ or shaking pals$).ti,ab,kf.
112	(pantothenate kinase-associated neurodegenerat$ or PKAN or hallervorden-spatz$).ti,ab,kf.
113	((neurodegeneration adj3 brain iron accumulation) or NBIA$1).ti,ab,kf.
114	(olivopontocerebellar atroph$ or OPCA or olivopontocerebellar degenerat$).ti,ab,kf.
115	(multiple system atrophy adj5 cerebellar).ti,ab,kf.
116	(alper$ adj (disease$ or syndrome$ or disorder$)).ti,ab,kf.
117	(progressive sclerosing poliodystroph$ or progressive infantile poliodystroph$).ti,ab,kf.
118	(diffuse cerebral sclerosis adj5 schilder$).ti,ab,kf.
119	(leigh$ adj (syndrome$ or disease$ or disorder$)).ti,ab,kf.
120	(subacute necrotizing encephalomyelopath$ or subacute necrotising encephalomyelopath$ or sub-acute necrotizing encephalomyelopath$ or sub-acute necrotising encephalomyelopath$ or SNEM).ti,ab,kf.
121	(aicardi-gouti?res or aicardia-gouti?res).ti,ab,kf.
122	(worster-drought$ or congenital suprabulbar pares$).ti,ab,kf.
123	multiple sclerosis/
124	(multiple sclerosis or disseminated sclerosis or encephalomyelitis disseminata$).ti,ab,kf.
125	(demyelinating adj (disease$ or syndrome$ or disorder$)).ti,ab,kf.
126	myoclonic epileps$.ti,ab,kf.
127	((lafora$ or merrf$ or unverricht-lundborg$ or janz$) adj (disease$ or syndrome$ or disorder$)).ti,ab,kf.
128	lennox-gastaut$.ti,ab,kf.
129	(lennox$ adj (syndrome$ or disease$ or disorder$)).ti,ab,kf.
130	(west$ adj (syndrome$ or disease$ or disorder$)).ti,ab,kf.
131	(epilepsia partialis continua or kojevnikov$ or epilepsia partialis continuoa or kozhevnikof$).ti,ab,kf.
132	Charcot-Marie-Tooth Disease/
133	(charcot-marie-tooth$ or peroneal muscular atroph$).ti,ab,kf.
134	(progressive neuropathic muscular atroph$ or hereditary peroneal nerve dysfunction$ or peroneal neuropath$).ti,ab,kf.
135	(hereditary sensory adj3 motor neuropath$).ti,ab,kf.
136	(hereditary motor adj3 sensory neuropath$).ti,ab,kf.
137	(infantile refsum or infantile phytanic acid storage).ti,ab,kf.
138	congenital myasth?eni$.ti,ab,kf.
139	(duchenne muscular dystroph$ or dmd).ti,ab,kf.

140	(limb-girdle or erb$ muscular dystroph$).ti,ab,kf.
141	(sarcoglycanopath$ or sarcoglycaopath$).ti,ab,kf.
142	(osteochondrodysplas$ or schwartz-jampel or chondrodystrophi$ myotoni$ or myotoni$ chondrodystrophi$).ti,ab,kf.
143	(congenita$ myotoni$ or myotoni$ congenita$).ti,ab,kf.
144	(thomsen$ adj (disease$ or disorder$ or syndrome$)).ti,ab,kf.
145	((recessive adj3 myotoni$) or becker$ myotoni$).ti,ab,kf.
146	(isaac$ adj (syndrome$ or disease$ or disorder$)).ti,ab,kf.
147	neuromyotoni$.ti,ab,kf.
148	(paramyotoni$ congenita$ or congenita$ paramyotoni$).ti,ab,kf.
149	(eulenburg$ adj (disease$ or syndrome$ or disorder$)).ti,ab,kf.
150	(myotoni$ adj (disease$ or disorder$ or syndrome$)).ti,ab,kf.
151	pseudomyotoni$.ti,ab,kf.
152	(congenital adj3 myopath$).ti,ab,kf.
153	myopathycongenital.ti,ab,kf.
154	((nemaline or rod) adj3 myopath$).ti,ab,kf.
155	((central core or mini-core or minicore or multicore or multi-core) adj (disease$ or disorder$ or syndrome$ or myopath$)).ti,ab,kf.
156	fiber type disproportion.ti,ab,kf.
157	fibre type disproportion.ti,ab,kf.
158	(congenital$ adj5 muscular dystroph$).ti,ab,kf.
159	((centronuclear or myotubular) adj myopath$).ti,ab,kf.
160	(mitochondrial myopath$ or mitochondrial encephalomyopath$ or chronic progressive external ophthalmopleg$).ti,ab,kf.
161	((melas or kearns-sayre$) adj (syndrome$ or disease$ or disorder$)).ti,ab,kf.
162	Quadriplegia/ and spastic$.ti,ab,kf.
163	(spastic quadriplegi$ or spastic tetraplegi$).ti,ab,kf.
164	(reye$ adj (syndrome$ or disease$ or disorder$)).ti,ab,kf.
165	multiple pterygium.ti,ab,kf.
166	((primary pulmonary or precapillary pulmonary or idiopathic pulmonary) adj (hypertension or ht or arterial hypertension)).ti,ab,kf.
167	((primary bronchopulmonary or precapillary bronchopulmonary or idiopathic bronchopulmonary) adj (hypertension or ht or arterial hypertension)).ti,ab,kf.
168	((primary lung or precapillary lung or idiopathic lung) adj (hypertension or ht or arterial hypertension)).ti,ab,kf.
169	ipah.ti,ab,kf.
170	((congestive or dilated) adj cardiomyopath$).ti,ab,kf.
171	(hypertrophic adj cardiomyopath$).ti,ab,kf.
172	(congenital adj3 cardiomyopath$).ti,ab,kf.
173	(restrictive cardiomyopath$ or obliterative cardiomyopath$ or constrictive cardiomyopath$).ti,ab,kf.
174	(pulmonary fibros$ or lung fibros$ or bronchopulmonary fibros$ or fibrosing alveolit$ or interstitial pneumonit$).ti,ab,kf.
175	(respiratory adj (failure$ or insufficienc$)).ti,ab,kf.
176	((cystic lung or cystic pulmonary or cystic bronchopulmonary) adj (disease$ or disorder or syndrome$)).ti,ab,kf.
177	(bronchogenic cyst$ or bronchopulmonary foregut malformation$).ti,ab,kf.

178	cystic adenomatoid malformation$.ti,ab,kf.
179	lobar emphysem$.ti,ab,kf.
180	(pulmonary sequestration$ or bronchopulmonary sequestration$ or lung sequestration$ or extralobar sequestration$ or extra-lobar sequestration$ or intralobar sequestration$ or intra-lobar sequestration$).ti,ab,kf.
181	pulmolithias$.ti,ab,kf.
182	((liver$1 or hepatic) adj3 fail$).ti,ab,kf.
183	exp "Cirrhosis (Liver)"/
184	(cirrhosis adj3 liver$1).ti,ab,kf.
185	((veno-occlusive or venous occlusive) adj (disease$ or syndrome$ or disorder$)).ti,ab,kf.
186	(swachman-diamond or shwachman-bodian or schwachmann-diamond or shwachmann-bodian).ti,ab,kf.
187	wegener$ granulomatos$.ti,ab,kf.
188	(granulomatos$ adj3 polyangiit$).ti,ab,kf.
189	essential osteolys$.ti,ab,kf.
190	((gorham$ or gorham-stout$ or vanishing bone or phantom bone) adj (disease$ or syndrome$ or disorder)).ti,ab,kf.
191	((arc or arthrogryposis renal dysfunction cholestasis) adj (disease$ or syndrome$ or disorder)).ti,ab,kf.
192	Cerebral Hemorrhage/ and congen$.mp.
193	Cerebral Hemorrhage/ and Birth Injuries/
194	(cerebral h?emorrhage$ and (birth$ adj3 injur$)).ti,ab,kf.
195	asphyxia neonatorum.ti,ab,kf.
196	((perinatal$ or neonatal$ or birth$) adj3 asphyxia$).ti,ab,kf.
197	congenital rubella.ti,ab,kf.
198	(congenital adj (cytomegalovirus$ or cmv)).ti,ab,kf.
199	Herpesvirus 3, Human/ and congenital$.ti,ab,kf.
200	((congenital or fetal or foetal) adj3 (varicella$ or chicken pox$ or VZV)).ti,ab,kf.
201	congenital toxoplasmos$.ti,ab,kf.
202	((brain$ or cerebral) adj3 hypoxi$).ti,ab,kf.
203	(congenital$ adj3 (kidney failure$ or renal failure$ or kidney insufficienc$ or renal insufficienc$)).ti,ab,kf.
204	(congenital$ adj3 (kidney disease$ or renal disease$)).ti,ab,kf.
205	Anencephaly/
206	(anencephal$ or meroanencephal$ or craniorachischis$).ti,ab,kf.
207	(aprosencephal$ adj3 open cranium).ti,ab,kf.
208	(encephalocele$ or cranium bifidum).ti,ab,kf.
209	dandy-walker$.ti,ab,kf.
210	(acrocallosal or acro-callosal or acrocolossal or acro colossal).ti,ab,kf.
211	(aicardi$ adj (syndrome$ or disease$ or disorder$)).ti,ab,kf.
212	(holoprosencephal$ or arhinencephal$ or holosprosencephal$).ti,ab,kf.
213	(hydranencephal$ or hydrancephal$ or hydroanencephal$).ti,ab,kf.
214	Microcephaly/
215	(lissencephal$ or walker-warburg$ or miller-dieker$ or norman-robert$ or microlissencephal$).ti,ab,kf.

216	((fukuyama$ or muscle-eye-brain) adj (syndrome$ or disease$ or disorder$)).ti,ab,kf.
217	(microgyria$ or microgyrus or micro-gyria$ or micro-gyrus).ti,ab,kf.
218	(pachygyria$ or pachgyria$).ti,ab,kf.
219	agyria$.ti,ab,kf.
220	((septo-optic or septooptic) adj dysplas$).ti,ab,kf.
221	de morsier$.ti,ab,kf.
222	(schizencephal$ or schizzencephal$).ti,ab,kf.
223	chiari$ malformation$.ti,ab,kf.
224	(truncus or common arterial trunk$).ti,ab,kf.
225	((transposition$ or dextrotransposition$ or dtransposition$ or levotransposition$ or ltransposition$) adj3 (great arter$ or main arter$ or aorta$ or pulmonary arter$ or great vessel$ or main vessel$)).ti,ab,kf.
226	(dextro-tga or d-tga or levo-tga or l-tga).ti,ab,kf.
227	(double inlet adj3 ventricle$).ti,ab,kf.
228	DILV.ti,ab,kf.
229	single ventricle$.ti,ab,kf.
230	(isomerism adj3 atrial appendage$).ti,ab,kf.
231	(aspleni$ or polyspleni$ or poly-spleni$).ti,ab,kf.
232	(tetralogy adj3 fallot$).ti,ab,kf.
233	(eisenmenger$ or tardive cyanos$ or eisenmeyer$).ti,ab,kf.
234	(pentalogy adj3 fallot$).ti,ab,kf.
235	((pulmonary or bronchopulmonary or lung$) adj3 atresia$).ti,ab,kf.
236	((tricuspid or tri) adj3 atresia$).ti,ab,kf.
237	(ebstein$ adj (anomal$ or malformation$)).ti,ab,kf.
238	(hypoplastic left heart adj (syndrome$ or disease$ or disorder$)).ti,ab,kf.
239	((aortic or aorta$) adj3 atresia$).ti,ab,kf.
240	(mitral adj3 atresia$).ti,ab,kf.
241	((absence$ or absent$) adj3 (aorta$ or aortic)).ti,ab,kf.
242	(aplas$ adj3 (aorta$ or aortic)).ti,ab,kf.
243	(((aorta$ or aortic) adj3 aneurys$) and congenital$).ti,ab,kf.
244	(hypoplas$ adj3 (aorta$ or aortic)).ti,ab,kf.
245	(convulsion$ adj3 (aorta$ or aortic)).ti,ab,kf.
246	(persistent right adj3 (aorta$ or aortic)).ti,ab,kf.
247	((anomalous pulmonary venous or anamolous pulmonary venous) adj (connection or drainage or return)).ti,ab,kf.
248	((absence$ or absent$) adj3 vena$ cava$).ti,ab,kf.
249	(persistent left adj3 cardinal vein$).ti,ab,kf.
250	((scimitar$ or pulmonary venolobar) adj (syndrome$ or disease$ or disorder$)).ti,ab,kf.
251	(arteriovenous malformations/ or intracranial arteriovenous malformations/) and bilateral.ti,ab,kf.
252	((bilateral AV or bilateral arteriovenous or bilateral arterio-venous) adj3 malform$).ti,ab,kf.
253	((trachea$ or windpipe$ or wind-pipe$) adj3 atresia$).ti,ab,kf.
254	((trachea$ or laryngotrachea$ or glottic or subglottic or sub-glottic) adj3 stenosis).ti,ab,kf.

255	((lung$ or pulmonary or bronchopulmonary) adj3 (hypoplas$ or dysplas$)).ti,ab,kf.
256	((absence$ or absent$) adj3 (esophag$ or oesophag$ or foodpipe or food-pipe$ or gullet$)).ti,ab,kf.
257	(duoden$ adj3 atresia$).ti,ab,kf.
258	((absence$ or absent$) adj3 (intestin$ or gastrointestin$)).ti,ab,kf.
259	((intestin$ or gastrointestin$) adj3 atresia$).ti,ab,kf.
260	((intestin$ or gastrointestin$) adj3 stenos$).ti,ab,kf.
261	(cloaca$ adj3 (abnor$ or malform$ or anomal$)).ti,ab,kf.
262	(cloaca$ adj3 exopthalmo$).ti,ab,kf.
263	(biliary adj3 atresia$).ti,ab,kf.
264	(extrahepatic ductopen$ or extra-hepatic ductopen$ or progressive obliterative cholangiopath$).ti,ab,kf.
265	(biliary adj3 hypoplas$).ti,ab,kf.
266	(alagille$ adj3 atresia$).ti,ab,kf.
267	((absence$ or absent$) adj3 kidney$).ti,ab,kf.
268	(potter$ adj (sequence$ or syndrome$ or disease$ or disorder$)).ti,ab,kf.
269	oligohydramn$.ti,ab,kf.
270	((kidney$ or renal) adj3 dysplas$).ti,ab,kf.
271	((meckel$ or meckelgruber$ or gruber$) adj (syndrome$ or disease$ or disorder$)).ti,ab,kf.
272	dysencephalia splanchnocystica$.ti,ab,kf.
273	(pena-shokeir$ or penn-shokeir$).ti,ab,kf.
274	(larsen$ adj (syndrome$ or disease$ or disorder$)).ti,ab,kf.
275	acrocephalosyndactyl$.ti,ab,kf.
276	(pfeiffer$ adj (syndrome$ or disease$ or syndrome$)).ti,ab,kf.
277	short rib$1.ti,ab,kf.
278	(saldino-noonan$ or majewski$ or verma-naumoff$ or beemer-langer$).ti,ab,kf.
279	(jeune$ adj (syndrome$ or disease$ or disorder$)).ti,ab,kf.
280	asphyxiating thoracic dysplas$.ti,ab,kf.
281	chondrodysplasia punctata$.ti,ab,kf.
282	((conradi$ or h?nermann$ or happle$) adj3 (syndrome$ or disease$ or disorder$)).ti,ab,kf.
283	osteogenesis imperfecta.ti,ab,kf.
284	((brittle bone or lobstein$) adj (disease$ or disorder$ or syndrome$)).ti,ab,kf.
285	(spondyloepimetaphyseal or spondyloepiphyseal or spendylo metaphyseal).ti,ab,kf.
286	(omphalocele$ or omphalocoele$ or exomphalos).ti,ab,kf.
287	(hernia$ adj3 umbilic$).ti,ab,kf.
288	gastroschis$.ti,ab,kf.
289	(lamellar$ adj3 ichthyos$).ti,ab,kf.
290	((harlequin$ or harloquin$) adj3 (ichthyos$ or baby or babies or f?etus$)).ti,ab,kf.
291	(ichthyosis congenita$ or ichthyosis fetalis or keratosis diffusa fetalis).ti,ab,kf.
292	epidermolysis bullosa$.ti,ab,kf.
293	(johanson-blizzard$ or johanna-blizzard$).ti,ab,kf.
294	xeroderma pigmentosum.ti,ab,kf.
295	lacrimo-auriculo-dento-digital.ti,ab,kf.

296	ectodermal dysplas$.ti,ab,kf.
297	((ladd or eec) adj (syndrome$ or disease$ or disorder$)).ti,ab,kf.
298	(sturge-weber or encephalotrigeminal angiomatos$).ti,ab,kf.
299	Fetal Alcohol Syndrome/
300	f?etal alcohol.ti,ab,kf.
301	pierre robin$.ti,ab,kf.
302	(acrocephalosyndact$ or acrocephalopolysyndact$).ti,ab,kf.
303	((apert$ or crouzon$ or saethre-chotzen$ or noack$ or carpenter$ or sakati-nyhan-tisdale$ or goodman$) adj (syndrome$ or disorder$ or disease$)).ti,ab,kf.
304	(fraser$ adj (syndrome$ or disease$ or disorder$)).ti,ab,kf.
305	cryptophthalmos.ti,ab,kf.
306	(cyclopia$1 or cyclocephal$ or synophthalmi$).ti,ab,kf.
307	(goldenhar$ or oculo-auriculo-vertebral).ti,ab,kf.
308	((m?bius$ or moebius$) adj (syndrome$ or disease$ or disorder$)).ti,ab,kf.
309	(orofaciodigital or oro-facial-digital or oral-facial-digital or papillon-league$ or psaume$).ti,ab,kf.
310	(robin$ adj (syndrome$ or disorder$ or disease$)).ti,ab,kf.
311	(freeman-sheldon$ or distal arthrogrypos$ or craniocarpotarsal dysplas$ or craniocarpotarsal dystroph$ or canio-carpo-tarsal or windmill-vane-hand$ or whistling-face).ti,ab,kf.
312	Cornelia De Lange Syndrome/
313	((de lange$ or bushy$) adj (syndrome$ or disorder$ or disease$)).ti,ab,kf.
314	amsterdam dwarfism.ti,ab,kf.
315	(aarskog or faciodigitogenital or facio-digito-genital or facial digital genital or shawl scrotum or faciogenital or facio-genital).ti,ab,kf.
316	(cockayne$ or neill-dingwall$).ti,ab,kf.
317	(cerebro-oculo-facio-skeletal or cerebro-oculo-facial-skeletal).ti,ab,kf.
318	(dubowitz$ adj (syndrome$ or disease$ or disorder$)).ti,ab,kf.
319	(robinow$ or robinhow$).ti,ab,kf.
320	(f?etal face or f?etal facies or f?etal faces or acral dysostos$ or mesomelic dwarfism or covesdem$).ti,ab,kf.
321	(silver-russell$ or russell-silver$).ti,ab,kf.
322	(silver$ adj (syndrome$ or disease$ or disorder$)).ti,ab,kf.
323	((seckel$ or harper$) adj (syndrome$ or disease$ or disorder$)).ti,ab,kf.
324	(microcephalic primordial dwarfism or bird-headed dwarf$ or virchow-seckel dwarfism).ti,ab,kf.
325	(smith-lemli-opitz$ or dehydrocholesterol reductase deficien$).ti,ab,kf.
326	Prader Willi Syndrome/
327	(prader-willi$ or pradar-willi$).ti,ab,kf.
328	(rubinstein-taybi$ or rubenstein-tabyii$ or broad thumb-hallux).ti,ab,kf.
329	((rubinstein$ or rubenstein$) adj2 (syndrome$ or disease$ or disorder$)).ti,ab,kf.
330	(alport$ adj (syndrome$ or disease$ or disorder$)).ti,ab,kf.
331	(hereditary nephritis or h?emorrhagic familial nephritis).ti,ab,kf.
332	(hereditary deafness adj3 nephropath$).ti,ab,kf.
333	(h?ematuria adj3 nephropath$ adj3 deafness).ti,ab,kf.
334	laurence-moon$.ti,ab,kf.

335	(bardet-biedl$ or biedl-bardet$).ti,ab,kf.
336	zellweger$.ti,ab,kf.
337	((cerebrohepatorenal or cerebro-hepato-renal) adj (syndrome$ or disease$ or disorder$)).ti,ab,kf.
338	(edward$ adj (syndrome$ or disease$ or disorder$)).ti,ab,kf.
339	"trisomy 18".ti,ab,kf.
340	(patau$ adj (syndrome$ or disease$ or disorder$)).ti,ab,kf.
341	("trisomy 13" or "trisomy D").ti,ab,kf.
342	"trisomy 22".ti,ab,kf.
343	"trisomy 9".ti,ab,kf.
344	"trisomy 10".ti,ab,kf.
345	duplication syndrome$.ti,ab,kf.
346	(("chromosome 8" or "chr 8") adj5 duplicat$).ti,ab,kf.
347	(("chromosome x" or "chr x") and duplicat$).ti,ab,kf.
348	(chromosom$ abnormality adj5 duplicat$).ti,ab,kf.
349	"tetrasomy 5p".ti,ab,kf.
350	(tetrasomy adj3 mosaic$).ti,ab,kf.
351	(delet$ adj5 short arm adj5 "chrom$ 4").ti,ab,kf.
352	((wolf-hirschhorn$ or wolff hirschorn$ or chromosome deletion dillan$ or pitt-rogers-dank$ or pitt$) adj3 (syndrome$ or disease$ or disorder$)).ti,ab,kf.
353	Crying Cat Syndrome/
354	((cri du chat$ or crying cat$ or 5p or lejeune$) adj3 (syndrome$ or disease$ or disorder$)).ti,ab,kf.
355	((jacobsen$ or 11q deletion) adj5 (syndrome$ or disease$ or disorder$)).ti,ab,kf.
356	(9p minus or 9p deletion).ti,ab,kf.
357	(alfi$ adj (syndrome$ or disease$ or disorder$)).ti,ab,kf.
358	(degouchy$ or de gouchy$ or degrouchy$ or de grouchy$).ti,ab,kf.
359	distal 18q.ti,ab,kf.
360	(ondine$ curse or congenital central hypoventilation or primary alveolar hypoventilation).ti,ab,kf.
361	(((graft vs host or graft versus host) adj (disease$ or syndrome$ or disorder)) and chronic$).ti,ab,kf.
362	Terminally Ill Patients/
363	Palliative Care/
364	Hospice/
365	(life adj2 limit$).ti,ab,kf.
366	(life adj2 threaten$).ti,ab,kf.
367	end of life.ti,ab,kf.
368	eol.ti,ab,kf.
369	(terminal$ adj2 (ill or illness$ or condition$1 or disease$1 or syndrome$ or disorder$)).ti,ab,kf.
370	(terminal adj2 (care$ or caring)).ti,ab,kf.
371	palliat$.ti,ab,kf.
372	(care adj2 dying).ti,ab,kf.
373	(technology adj2 dependent).ti,ab,kf.
374	hospice$.ti,ab,kf.

375	(severe adj2 (need or needs or illness$ or disease$1 or disabilit$ or impairment$1 or impediment$1 or condition$1 or disadvant$ or problem$1 or syndrome$1 or disorder$1)).ti,ab,kf.
376	(complex adj2 (need or needs or illness$ or disease$1 or disabilit$ or impairment$1 or impediment$1 or condition$1 or disadvant$ or problem$1 or syndrome$1 or disorder$1)).ti,ab,kf.
377	(rare adj2 (illness$ or disease$ or disabilit$ or impairment$ or impediment$ or condition$1 or syndrome$1 or disorder$1)).ti,ab,kf.
378	(multiple adj2 (need or needs or illness$ or disease$1 or disabilit$ or impairment$1 or impediment$ or condition$1 or disadvant$ or health or syndrome$1 or disorder$1)).ti,ab,kf.
379	(profound adj2 (need or needs or illness$ or disease$ or disabilit$ or impairment$ or impediment$ or condition$1 or syndrome$1 or disorder$1)).ti,ab,kf.
380	(intense adj2 (need or needs or illness$ or disease$ or disabilit$ or impairment$ or impediment$ or condition$1 or syndrome$1 or disorder$1)).ti,ab,kf.
381	(serious adj2 (disabilit$ or impairment$ or impediment$ or condition$1 or disadvant$)).ti,ab,kf.
382	exp HIV/
383	(HIV or human immunodeficiency virus$).ti,ab,kf.
384	(htlv or human t-lymphotropic virus$ or human t cell lymphotropic virus$).ti,ab,kf.
385	(acquired immune deficiency syndrome$ or acquired immunodeficiency syndrome$).ti,ab,kf.
386	(AIDS adj3 (virus$ or infection$)).ti,ab,kf.
387	(AIDS adj (related or associated)).ti,ab,kf.
388	exp Neoplasms/
389	(cancer$ or carcin$ or tumor$ or tumour$ or neoplas$ or adenocarcin$ or oncol$ or malignan$).ti,ab,kf.
390	Cystic Fibrosis/
391	(cystic fibrosis or fibrocystic or fibro-cystic or mucoviscidosis or cf).ti,ab,kf.
392	Cerebral Palsy/
393	(cerebr$ adj3 pals$).ti,ab,kf.
394	spasticit$.ti,ab,kf.
395	Quadriplegia/
396	(spastic$ and (quadripleg$ or tetrapleg$)).ti,ab,kf.
397	((kidney$ or renal) adj3 (failure$ or insufficienc$)).ti,ab,kf.
398	(end stage adj3 (kidney or renal)).ti,ab,kf.
399	(("stage 5" or "stage V") adj3 (kidney or renal)).ti,ab,kf.
400	(ESRD or ESKD or ESRF or ESKF or CRF or CKF).ti,ab,kf.
401	or/1-400
402	((Young adj1 people$) or Youth$ or Care leaver$ or residential child$ or Adolescen$ or Young adult$ or Young person$ or Young men$ or Young women$ or Teen$ or juvenile$ or Younger people or Youngster$ or Looked after or Child welfare or paediatric$ or pediatric$ or peadiatric$ or Young male$ or Young female$ or juvenile or children$ or child or childhood or (young adj1 patient$) or young carer$ or minors or puber$ or pubescen$ or ((secondary or high*) adj2 (school* or education))).ti,ab.
403	pediatrics/
404	402 or 403

405	((transition$ or transfer$ or handoff or handover or hand over) and (Service$ or care or clinic$ or healthcare or hospital$ or center$ or centre$ or facility or facilities or unit$ or department$ or institution$ or agency or agencies or hospice$ or provider$ or program$ or Coordinat$ or Framework$ or Managing or Managed or preparedness or Planning or Preparing or Preparation$ or Plan$ or Protocol$ or planned or Support or Supporting or Trajectory or Trajectories or Pathway$ or Process or Processes or Readiness or Partnership$ or programme$ or program$ or training or strateg$ or Failure$ or Barrier$ or system?)).ti.
406	((transition$ or transfer$ or handoff or handover or hand over) adj3 (Service$ or care or clinic$ or healthcare or hospital$ or center$ or centre$ or facility or facilities or unit$ or department$ or institution$ or agency or agencies or hospice$ or provider$ or program$ or Coordinat$ or Framework$ or Managing or Managed or preparedness or Planning or Preparing or Preparation$ or Plan$ or Protocol$ or planned or Support or Supporting or Trajectory or Trajectories or Pathway$ or Process or Processes or Readiness or Partnership$ or programme$ or program$ or training or strateg$ or Failure$ or Barrier$ or system?)).ab.
407	(continu$ and (care or healthcare or Support or Supporting or Failure$ or Barrier$)).ti.
408	(continu$ adj3 (care or healthcare or Support or Supporting or Failure$ or Barrier$)).ab.
409	Continuum of Care/
410	patient handoff/
411	Treatment Planning/
412	Patient transfer/
413	(transition$ or transfer$ or handoff or handover or hand$ over).ti,ab.
414	(409 or 411) and 413
415	or/405-408,410,412,414
416	401 and 404 and 415
417	limit 416 to (english language and yr="1990 -Current")

CINAHL (EBSCOHost)

Concepts:

1. LLC: lines 1-525
2. Child/young adult: lines 526-536
3. Transition: lines 537-547

1	MH "Creutzfeldt-Jakob Syndrome"
2	TI (creutzfeldt-jakob* or jakob-creutzfeldt* or cjd or spongiform encephalopath*) OR AB ((creutzfeldt-jakob* or jakob-creutzfeldt* or cjd or spongiform encephalopath*)
3	MH “Subacute Sclerosing Panencephalitis”
4	TI (subacute sclerosing panencephalit* or sub-acute sclerosing panencephalit* or sspe or subacute sclerosing leukoencephalit* or sub-acute sclerosing leukoencephalit* or van bogaert* leukoencephalit* or measles inclusion body encephalit* or mibe) OR AB (subacute sclerosing panencephalit* or sub-acute sclerosing panencephalit* or sspe or subacute sclerosing leukoencephalit* or sub-acute sclerosing leukoencephalit* or van bogaert* leukoencephalit* or measles inclusion body encephalit* or mibe)
5	MH "beta-Thalassemia"
6	TI (beta N (thalass#emi* or thalas#emi*)) OR AB (beta N (thalass#emi* or thalas#emi*))
7	TI ((thalass#emi* or thalas#emi*) N1 major) OR AB ((thalass#emi* or thalas#emi*) N1 major)
8	MH “Anemia, Aplastic”
9	TI ((hypoplastic or aplastic) N1 an#emi*) OR AB ((hypoplastic or aplastic) N1 an#emi*)
10	TI (medullary N3 hypoplas*) OR AB (medullary N3 hypoplas*)
11	MH "Neutropenia+"
12	TI ((severe or chronic*) N3 neutropeni*) OR AB ((severe or chronic*) N3 neutropeni*)
13	MH "immunologic deficiency syndromes" OR MH "acquired immunodeficiency syndrome"
14	TI (immun* deficiency N1 (syndrome* or disease* or disorder*)) OR AB (immun* deficiency N1 (syndrome* or disease* or disorder*))
15	TI (immunodeficiency N1 (syndrome* or disease* or disorder*)) OR AB (immunodeficiency N1 (syndrome* or disease* or disorder*))
16	MH "DiGeorge Syndrome"
17	TI (digeorge* or di george* or sedlackova* or opitz g-bbb or velocardiofacial or velo-cardiofacial or velo-cardio-facial or shprintzen* or ctaf) OR AB (digeorge* or di george* or sedlackova* or opitz g-bbb or velocardiofacial or velo-cardiofacial or velo-cardio-facial or shprintzen* or ctaf)
18	TI ((deletion or vcf or pharyngeal pouch or thymic aplasia or anomaly face) N1 (syndrome* or disease* or disorder*)) OR AB (((deletion or vcf or pharyngeal pouch or thymic aplasia or anomaly face) N1 (syndrome* or disease* or disorder*))
19	MH "Common Variable Immunodeficiency"

20	TI ((common variable or late onset) N3 (immunodeficienc* or immune deficienc* or immunoglobulin deficienc* or hypogammaglobulin*)) OR AB ((common variable or late onset) N3 (immunodeficienc* or immune deficienc* or immunoglobulin deficienc* or hypogammaglobulin*))
21	TI (acquired hypogammaglobulin*) OR AB (acquired hypogammaglobulin*)
22	TI (cryoglobulin#em*) OR AB (cryoglobulin#em*)
23	TI ((autoimmune or failure*) N3 (polyglandular* or polyendocrin*)) OR AB ((autoimmune or failure*) N3 (polyglandular* or polyendocrin*))
24	MH "Hutchinson-Gilford Progeria Syndrome"
25	TI (progeria or hutchinson-gilford*) OR AB (progeria or hutchinson-gilford*)
26	TI (tyrosin#em*) OR AB (tyrosin#em*)
27	MH "Maple Syrup Urine Disease"
28	TI (maple syrup urine or msud) OR AB (maple syrup urine or msud)
29	TI (branched chain) OR AB (branched chain)
30	TI (bckd N5 (deficienc* or ketoacid* or keto-acid*)) OR AB (bckd N5 (deficienc* or ketoacid* or keto-acid*))
31	TI (hyperleucine-isoleucin*) OR AB (hyperleucine-isoleucin*)
32	MH "Methylmalonic Acid"
33	TI (methylmalonic acid#emi* or methylmalonic aciduri* or methyl malonic acid#emi* or methyl malonic aciduri*) OR AB (methylmalonic acid#emi* or methylmalonic aciduri* or methyl malonic acid#emi* or methyl malonic aciduri*)
34	TI (propionic acid#em* or propionic acidur* or propionyl-CoA carboxylase deficienc* or ketotic glycin#em*) OR AB (propionic acid#em* or propionic acidur* or propionyl-CoA carboxylase deficienc* or ketotic glycin#em*)
35	MH "Adrenoleukodystrophy"
36	TI (adrenoleukodystroph* or x-ald or schilder-addison* or addison-schilder* or adrenomyeloneuropath*) OR AB (adrenoleukodystroph* or x-ald or schilder-addison* or addison-schilder* or adrenomyeloneuropath*)
37	TI ((carnitine palmityltransferase or carnitine palmitoyltransferase or carnitine o-palmityltransferase or carnitine o-palmitoyltransferase) N3 deficienc*) OR AB ((carnitine palmityltransferase or carnitine palmitoyltransferase or carnitine o-palmityltransferase or carnitine o-palmitoyltransferase) N3 deficienc*)
38	MH "Fanconi Syndrome"
39	TI (fanconi* N (syndrome* or disease* or disorder*)) OR AB (fanconi* N (syndrome* or disease* or disorder*))
40	TI (ocular N3 (renal or kidney)) OR AB (ocular N3 (renal or kidney))
41	TI (cystinos* or cystine storage or cystine diathes* or cystine disease*) OR AB (cystinos* or cystine storage or cystine diathes* or cystine disease*)
42	MH "Oculocerebrorenal Syndrome"
43	TI ((lowe or lowes or oculocerebrorenal or cerebrooculorenal or cerebro-oculo-renal) N3 (syndrome* or disease* or disorder*)) OR AB ((lowe or lowes or oculocerebrorenal or cerebrooculorenal or cerebro-oculo-renal) N3 (syndrome* or disease* or disorder*))
44	MH "Metalloproteins/df"
45	MH "Molybdenum/df"
46	TI (molybdenum cofactor deficien* or molybdenum co-factor deficien*) OR AB (molybdenum cofactor deficien* or molybdenum co-factor deficien*)

47	TI ((sulphite* or sulfite*) N3 oxidase deficien*) OR AB ((sulphite* or sulfite*) N3 oxidase deficien*)
48	MH "Argininosuccinic Acid"
49	TI (argininosuccinic acidur* or argininosuccinic acid#emi*) OR AB (argininosuccinic acidur* or argininosuccinic acid#emi*)
50	TI (citrullin#emi* or citrullinuri*) OR AB (citrullin#emi* or citrullinuri*)
51	MH "Amino Acid Metabolism, Inborn Errors"
52	TI (glutaric acid#emi* or glutaric aciduri*) OR AB (glutaric acid#emi* or glutaric aciduri*)
53	TI (glycine encephalopath* or non-ketotic hyperglycin#emi* or nonketotic hyperglycin#emi*) OR AB (glycine encephalopath* or non-ketotic hyperglycin#emi* or nonketotic hyperglycin#emi*)
54	TI (arginin#emi* or arginase deficien* or hyperarginin#emi*) OR AB (arginin#emi* or arginase deficien* or hyperarginin#emi*)
55	MH "Renal Aminoacidurias"
56	TI (aminoaciduri* or aminoacid#emi*) OR AB (arginin#emi* or arginase deficien* or hyperarginin#emi*)
57	MH "glycogen storage disease+"
58	TI (glycogen storage N1 (disease* or syndrome* or disorder*)) OR AB (glycogen storage N1 (disease* or syndrome* or disorder*))
59	TI (pompe* N1 (disease* or syndrome* or disorder*)) OR AB (pompe* N1 (disease* or syndrome* or disorder*))
60	MH "Galactosemia"
61	TI (galactos#emi*) OR AB (galactos#emi*)
62	MH "Pyruvate Dehydrogenase Complex Deficiency Disease"
63	TI (pyruvate dehydrogenase N3 deficien*) OR AB (pyruvate dehydrogenase N3 deficien*)
64	TI (oxalosis and (renal or kidney*)) OR AB (oxalosis and (renal or kidney*))
65	TI (gangliosidos*) OR AB (gangliosidos*)
66	TI (sandhoff* N1 (disease* or syndrome* or disorder*)) OR AB (sandhoff* N1 (disease* or syndrome* or disorder*))
67	TI (tay sach*) OR AB (tay sach*)
68	MH "Mucolipidoses"
69	TI (mucolipidos*) OR AB (mucolipidos*)
70	TI (canavan* leucodystroph* or aspartoacylase deficien* or aminoacylase 2 deficien*) OR AB (canavan* leucodystroph* or aspartoacylase deficien* or aminoacylase 2 deficien*)
71	TI ((canavan* or canavan-van bogaert-bertrand*) N (disease* or syndrome* or disorder*)) OR AB ((canavan* or canavan-van bogaert-bertrand*) N (disease* or syndrome* or disorder*))
72	MH "Gaucher Disease"
73	TI (gaucher* N1 (disease* or syndrome* or disorder*)) OR AB (gaucher* N1 (disease* or syndrome* or disorder*))
74	TI (glucocerebrosidase deficien* or glucosylceramidase deficien*) OR AB (glucocerebrosidase deficien* or glucosylceramidase deficien*)

75	TI (metachromatic leukodystroph* or arylsulfatase A deficien* or metachromic leukodystroph*) OR AB (metachromatic leukodystroph* or arylsulfatase A deficien* or metachromic leukodystroph*)
76	MH "Niemann-Pick Diseases+"
77	TI (niemann-pick* or sphingomyelinase deficien*) OR AB (niemann-pick* or sphingomyelinase deficien*)
78	MH "Sphingolipidoses"
79	TI (sphingolipidos*) OR AB (sphingolipidos*)
80	MH "Fabry Disease"
81	TI (fabry* N1 (disease* or syndrome* or disorder*)) OR AB (fabry* N1 (disease* or syndrome* or disorder*))
82	TI (angiokeratoma corporis diffusum or alpha-galactosidase A deficien*) OR AB (angiokeratoma corporis diffusum or alpha-galactosidase A deficien*)
83	MH"Leukodystrophy, Globoid Cell"
84	TI (krabbe* N1 (disease* or syndrome* or disorder*)) OR AB (krabbe* N1 (disease* or syndrome* or disorder*))
85	TI (globoid cell leukodystroph* or galactosylceramide lipidos* or galactosylcerebrosidase deficien* or galactosylceramidase deficien*) OR AB (globoid cell leukodystroph* or galactosylceramide lipidos* or galactosylcerebrosidase deficien* or galactosylceramidase deficien*)
86	TI (farber* N1 (disease* or syndrome* or disorder*)) OR AB (farber* N1 (disease* or syndrome* or disorder*))
87	TI (farber* lipogranulomatos* or ceramidase deficien* or fibrocytic dysmucopolysaccharidos*) OR AB (farber* lipogranulomatos* or ceramidase deficien* or fibrocytic dysmucopolysaccharidos*)
88	TI (pelizaeus-merzbacher*) OR AB (pelizaeus-merzbacher*)
89	TI (sulfatase deficien* or sulphatase deficien* or mucosulfatidos*) OR AB (sulfatase deficien* or sulphatase deficien* or mucosulfatidos*)
90	TI (austin* N1 (disease* or syndrome* or disorder*)) OR AB (austin* N1 (disease* or syndrome* or disorder*))
91	TI (sulfatidos*) OR AB (sulfatidos*)
92	TI (sea-blue histiocyt*) OR AB (sea-blue histiocyt*)
93	MH "Neuronal Ceroid-Lipofuscinoses"
94	TI (batten* N1 (disease* or syndrome* or disorder*)) OR AB (batten* N1 (disease* or syndrome* or disorder*))
95	TI (neuronal ceroid lipofuscinos* or santavuori-haltia* or jansky-bielschowsky* or bielschowsky-jansky*) OR AB (neuronal ceroid lipofuscinos* or santavuori-haltia* or jansky-bielschowsky* or bielschowsky-jansky*)
96	TI (kuf* N1 (disease* or syndrome* or disorder*)) OR AB (kuf* N1 (disease* or syndrome* or disorder*))
97	TI (spielmeyer vogt*) OR AB (spielmeyer vogt*)
98	MH "Lipid Metabolism, Inborn Errors"
99	TI ((cerebrotendineous or cerebrotendinous or cerebrotendious or cerebral) N3 (xanthomatos* or cholesteros*)) OR AB ((cerebrotendineous or cerebrotendinous or cerebrotendious or cerebral) N3 (xanthomatos* or cholesteros*))
100	TI (bogaert-scherer-epstein*) OR AB (bogaert-scherer-epstein*)

101	TI (wolman* N1 (disease* or syndrome* or disorder*)) OR AB (wolman* N1 (disease* or syndrome* or disorder*))
102	TI (lysosomal acid lipase deficien*) OR AB (lysosomal acid lipase deficien*)
103	MH "Mucopolysaccharidoses+"
104	TI (mucopolysaccharidos*) OR AB (mucopolysaccharidos*)
105	TI (hurler* N2 (syndrome* or disease* or disorder*)) OR AB (hurler* N2 (syndrome* or disease* or disorder*))
106	TI (hunter* N2 (syndrome* or disease* or disorder*)) OR AB (hunter* N2 (syndrome* or disease* or disorder*))
107	TI (MPS1 or MPS2 or MPS3 or MPS4 or MPS5 or MPS6 or MPS7 or MPS-1 or MPS-2 or MPS-3 or MPS-4 or MPS-5 or MPS-6 or MPS-7 or MPSI or MPSII or MPSIII or MPSIV or MPSV or MPSVI or MPSVII or MPS-I or MPS-II or MPS-III or MPS-IV or MPS-V or MPS-VI or MPS-VII) OR AB (MPS1 or MPS2 or MPS3 or MPS4 or MPS5 or MPS6 or MPS7 or MPS-1 or MPS-2 or MPS-3 or MPS-4 or MPS-5 or MPS-6 or MPS-7 or MPSI or MPSII or MPSIII or MPSIV or MPSV or MPSVI or MPSVII or MPS-I or MPS-II or MPS-III or MPS-IV or MPS-V or MPS-VI or MPS-VII)
108	TI (beta glucuronidase deficien* or sly syndrome* or sly disorder* or sly disease*) OR AB (beta glucuronidase deficien* or sly syndrome* or sly disorder* or sly disease*)
109	TI (maroteaux-lamy* or marotaeux-lamy* or polydystrophic dwarfism) OR AB (maroteaux-lamy* or marotaeux-lamy* or polydystrophic dwarfism)
110	TI (morquio* or moriquio* or beta galactosidase deficien*) OR AB (morquio* or moriquio* or beta galactosidase deficien*)
111	TI (sanfilippo* or sanfillipo*) OR AB (sanfilippo* or sanfillipo*)
112	MH "Mucolipidoses"
113	TI (mucolipidos* or pseudo-hurler* or pseudohurler*) OR AB (mucolipidos* or pseudo-hurler* or pseudohurler*)
114	TI ((inclusion-cell or i-cell) N1 (disease* or syndrome* or disorder*)) OR AB ((inclusion-cell or i-cell) N1 (disease* or syndrome* or disorder*))
115	TI (fucosidos* or fucidos*) OR AB (fucosidos* or fucidos*)
116	TI ((cdg or ctg) N1 (disease* or disorder* or syndrome*)) OR AB ((cdg or ctg) N1 (disease* or disorder* or syndrome*))
117	TI (carbohydrate-deficient glycoprotein N (disease* or disorder* or syndrome*)) OR AB (carbohydrate-deficient glycoprotein N (disease* or disorder* or syndrome*))
118	TI (congenital disorder* N3 glycosylation) OR AB (congenital disorder* N3 glycosylation)
119	TI (juvenile gout) OR AB (juvenile gout)
120	MH "Kinky Hair Syndrome"
121	TI (menkes*) OR AB (menkes*)
122	TI ((copper transport or steely hair or kinky hair) N1 (disease* or syndrome* or disorder*)) OR AB ((copper transport or steely hair or kinky hair) N1 (disease* or syndrome* or disorder*))
123	MH "alpha 1-Antitrypsin Deficiency"
124	TI (antitrypsin deficien* or A1AD) OR AB (antitrypsin deficien* or A1AD)
125	TI (AAT deficien* or alpha-1 protease deficien*) OR AB (AAT deficien* or alpha-1 protease deficien*)
126	TI (bisalbumin#emi*) OR AB (bisalbumin#emi*)
127	MH "Lipodystrophy, Congenital Generalized"

128	TI (congenital generali#ed lipodystroph* or berardinelli* or bernardnelli*) OR AB (congenital generali#ed lipodystroph* or berardinelli* or bernardnelli*)
129	MH "Landau-Kleffner Syndrome"
130	TI (landau-kleffner* or infantile acquired aphasia* or acquired epileptic aphasia*) OR AB (landau-kleffner* or infantile acquired aphasia* or acquired epileptic aphasia*)
131	TI (aphasia* N5 convulsive) OR AB (aphasia* N5 convulsive)
132	MH "Rett Syndrome"
133	TI (rett* N (syndrome* or disease* or disorder*)) OR AB (rett* N (syndrome* or disease* or disorder*))
134	TI (cerebroatrophic hyperammon#emi*) OR AB (cerebroatrophic hyperammon#emi*)
135	MH "Huntington's Disease"
136	TI (huntington*) OR AB (huntington*)
137	MH "Spinocerebellar Ataxias+"
138	TI ((nyhan* or kelley-seegmiller*) N1 (syndrome* or disorder* or disease*)) OR AB ((nyhan* or kelley-seegmiller*) N1 (syndrome* or disorder* or disease*))
139	TI (spinocerebellar ataxia* or ataxia* telangiectasia* or louis-bar* syndrome* or louis-bar* disease* or louis-bar* disorder* or machado-joseph* or joseph* disease* or joseph* disorder* or joseph* syndrome*) OR AB (spinocerebellar ataxia* or ataxia* telangiectasia* or louis-bar* syndrome* or louis-bar* disease* or louis-bar* disorder* or machado-joseph* or joseph* disease* or joseph* disorder* or joseph* syndrome*)
140	MH "Friedreich's Ataxia"
141	TI ((friedreich* or friedrich*) N3 ataxia*) OR AB ((friedreich* or friedrich*) N3 ataxia*)
142	TI (spinocerebellar degenerat*) OR AB (spinocerebellar degenerat*)
143	TI (spinal muscular atroph* or werdnig hoffman*) OR AB (spinal muscular atroph* or werdnig hoffman*)
144	TI (dubowitz* or kugelberg-welander*) OR AB (dubowitz* or kugelberg-welander*)
145	MH "Bulbar Palsy, Progressive"
146	TI (fazio-londe* or faziolonde* or progressive bulbar pals*) OR AB (fazio-londe* or faziolonde* or progressive bulbar pals*)
147	MH "parkinson disease" OR MH "parkinson disease, secondary"
148	TI (parkinson* or hypokinetic rigid syndrome* or hypokinetic rigid disease* or hypokinetic rigid disorder* or paralysis agitan* or shaking pals*) OR AB (parkinson* or hypokinetic rigid syndrome* or hypokinetic rigid disease* or hypokinetic rigid disorder* or paralysis agitan* or shaking pals*)
149	TI (pantothenate kinase-associated neurodegenerat* or PKAN or hallervorden-spatz*) OR AB (pantothenate kinase-associated neurodegenerat* or PKAN or hallervorden-spatz*)
150	TI ((neurodegeneration N3 brain iron accumulation) or NBIA#) OR AB ((neurodegeneration N3 brain iron accumulation) or NBIA#)
151	TI (olivopontocerebellar atroph* or OPCA or olivopontocerebellar degenerat*) OR AB (olivopontocerebellar atroph* or OPCA or olivopontocerebellar degenerat*)
152	TI (multiple system atrophy N5 cerebellar) OR AB (multiple system atrophy N5 cerebellar)

153	TI (alper* N1 (disease* or syndrome* or disorder*)) OR AB (alper* N1 (disease* or syndrome* or disorder*))
154	TI (progressive sclerosing poliodystroph* or progressive infantile poliodystroph*) OR AB (progressive sclerosing poliodystroph* or progressive infantile poliodystroph*)
155	TI (diffuse cerebral sclerosis N5 schilder*) OR AB (diffuse cerebral sclerosis N5 schilder*)
156	MH "Leigh Disease"
157	TI (leigh* N (syndrome* or disease* or disorder*)) OR AB (leigh* N (syndrome* or disease* or disorder*))
158	TI (subacute necrotizing encephalomyelopath* or subacute necrotising encephalomyelopath* or sub-acute necrotizing encephalomyelopath* or sub-acute necrotising encephalomyelopath* or SNEM) OR AB (subacute necrotizing encephalomyelopath* or subacute necrotising encephalomyelopath* or sub-acute necrotizing encephalomyelopath* or sub-acute necrotising encephalomyelopath* or SNEM)
159	TI (aicardi-gouti#res or aicardia-gouti#res) OR AB (aicardi-gouti#res or aicardia-gouti#res)
160	TI (worster-drought* or congenital suprabulbar pares*) OR AB (worster-drought* or congenital suprabulbar pares*)
161	MH "multiple sclerosis" OR MH "multiple sclerosis, chronic progressive" OR MH "multiple sclerosis, relapsing-remitting"
162	TI (multiple sclerosis or disseminated sclerosis or encephalomyelitis disseminata*) OR AB (multiple sclerosis or disseminated sclerosis or encephalomyelitis disseminata*)
163	TI (demyelinating N1 (disease* or syndrome* or disorder*)) OR AB (demyelinating N1 (disease* or syndrome* or disorder*))
164	MH "Epilepsies, Myoclonic+"
165	TI (myoclonic epileps*) OR AB (myoclonic epileps*)
166	TI ((lafora* or merrf* or unverricht-lundborg* or janz*) N1 (disease* or syndrome* or disorder*)) OR AB ((lafora* or merrf* or unverricht-lundborg* or janz*) N1 (disease* or syndrome* or disorder*))
167	TI (lennox-gastaut*) OR AB (lennox-gastaut*)
168	TI (lennox* N1 (syndrome* or disease* or disorder*)) OR AB (lennox* N1 (syndrome* or disease* or disorder*))
169	MH "Spasms, Infantile"
170	TI (west* N1 (syndrome* or disease* or disorder*)) OR AB (west* N1 (syndrome* or disease* or disorder*))
171	TI (epilepsia partialis continua or kojevnikov* or epilepsia partialis continuoa or kozhevnikof*) OR AB (epilepsia partialis continua or kojevnikov* or epilepsia partialis continuoa or kozhevnikof*)
172	MH "Charcot-Marie-Tooth Disease"
173	TI (charcot-marie-tooth* or peroneal muscular atroph*) OR AB (charcot-marie-tooth* or peroneal muscular atroph*)
174	TI (progressive neuropathic muscular atroph* or hereditary peroneal nerve dysfunction* or peroneal neuropath*) OR AB (progressive neuropathic muscular atroph* or hereditary peroneal nerve dysfunction* or peroneal neuropath*)
175	MH "Neuropathies, Hereditary Motor and Sensory"

176	TI (hereditary sensory N3 motor neuropath*) OR AB (hereditary sensory N3 motor neuropath*)
177	TI (hereditary motor N3 sensory neuropath*) OR AB (hereditary motor N3 sensory neuropath*)
178	MH "Peroxisomal Disorders"
179	TI (infantile refsum or infantile phytanic acid storage) OR AB (infantile refsum or infantile phytanic acid storage)
180	TI (congenital myasth#eni*) OR AB (congenital myasth#eni*)
181	MH "Muscular Dystrophy, Duchenne"
182	TI (duchenne muscular dystroph* or dmd) OR AB (duchenne muscular dystroph* or dmd)
183	TI (limb-girdle or erb* muscular dystroph*) OR AB (limb-girdle or erb* muscular dystroph*)
184	TI (sarcoglycanopath* or sarcoglycaopath*) OR AB (sarcoglycanopath* or sarcoglycaopath*)
185	MH "Osteochondrodysplasias"
186	TI (osteochondrodysplas* or schwartz-jampel or chondrodystrophi* myotoni* or myotoni* chondrodystrophi*) OR AB (osteochondrodysplas* or schwartz-jampel or chondrodystrophi* myotoni* or myotoni* chondrodystrophi*)
187	TI (congenita* myotoni* or myotoni* congenita*) OR AB (congenita* myotoni* or myotoni* congenita*)
188	TI (thomsen* N1 (disease* or disorder* or syndrome*)) OR AB (thomsen* N1 (disease* or disorder* or syndrome*))
189	TI ((recessive N3 myotoni*) or becker* myotoni*) OR AB ((recessive N3 myotoni*) or becker* myotoni*)
190	MH "Isaacs’ Syndrome"
191	TI (isaac* N1 (syndrome* or disease* or disorder*)) OR AB (isaac* N1 (syndrome* or disease* or disorder*))
192	TI (neuromyotoni*) OR AB (neuromyotoni*)
193	MH "Myotonic Disorders"
194	TI (paramyotoni* congenita* or congenita* paramyotoni*) OR AB (paramyotoni* congenita* or congenita* paramyotoni*)
195	TI (eulenburg* N1 (disease* or syndrome* or disorder*)) OR AB (eulenburg* N1 (disease* or syndrome* or disorder*))
196	TI (myotoni* N1 (disease* or disorder* or syndrome*)) OR AB (myotoni* N1 (disease* or disorder* or syndrome*))
197	TI (pseudomyotoni*) OR AB (pseudomyotoni*)
198	TI (congenital N3 myopath*) OR AB (congenital N3 myopath*)
199	TI (myopathycongenital) OR AB (myopathycongenital)
200	TI ((nemaline or rod) N3 myopath*) OR AB ((nemaline or rod) N3 myopath*)
201	TI ((central core or mini-core or minicore or multicore or multi-core) N1 (disease* or disorder* or syndrome* or myopath*)) OR AB ((central core or mini-core or minicore or multicore or multi-core) N1 (disease* or disorder* or syndrome* or myopath*))
202	TI (fiber type disproportion) OR AB (fiber type disproportion)
203	TI (fibre type disproportion) OR AB (fibre type disproportion)
204	MH "Muscular Dystrophy" AND (TI (congen*) OR AB (congen*))
205	TI (congenital* N5 muscular dystroph*) OR AB (congenital* N5 muscular dystroph*)

206	TI ((centronuclear or myotubular) N1 myopath*) OR AB ((centronuclear or myotubular) N1 myopath*)
207	MH "Mitochondrial Myopathies+"
208	TI (mitochondrial myopath* or mitochondrial encephalomyopath* or chronic progressive external ophthalmopleg*) OR AB (mitochondrial myopath* or mitochondrial encephalomyopath* or chronic progressive external ophthalmopleg*)
209	TI ((melas or kearns-sayre*) N1 (syndrome* or disease* or disorder*)) OR AB ((melas or kearns-sayre*) N1 (syndrome* or disease* or disorder*))
210	MH "Quadriplegia" AND (TI (spastic*) OR AB (spastic*))
211	TI (spastic quadriplegi* or spastic tetraplegi*) OR AB (spastic quadriplegi* or spastic tetraplegi*)
212	MH "Reye's Syndrome"
213	TI (reye* N1 (syndrome* or disease* or disorder*)) OR AB (reye* N1 (syndrome* or disease* or disorder*))
214	TI (multiple pterygium) OR AB (multiple pterygium)
215	MH "Hypertension, Pulmonary" AND (TI (primary*) OR AB (primary*))
216	TI ((primary pulmonary or precapillary pulmonary or idiopathic pulmonary) N1 (hypertension or ht or arterial hypertension)) OR AB ((primary pulmonary or precapillary pulmonary or idiopathic pulmonary) N1 (hypertension or ht or arterial hypertension))
217	TI ((primary bronchopulmonary or precapillary bronchopulmonary or idiopathic bronchopulmonary) N1 (hypertension or ht or arterial hypertension)) OR AB ((primary bronchopulmonary or precapillary bronchopulmonary or idiopathic bronchopulmonary) N1 (hypertension or ht or arterial hypertension))
218	TI ((primary lung or precapillary lung or idiopathic lung) N1 (hypertension or ht or arterial hypertension)) OR AB ((primary lung or precapillary lung or idiopathic lung) N1 (hypertension or ht or arterial hypertension))
219	TI (ipah) OR AB (ipah)
220	MH "Cardiomyopathy, Dilated"
221	TI ((congestive or dilated) N1 cardiomyopath*) OR AB ((congestive or dilated) N1 cardiomyopath*)
222	MH "Cardiomyopathy, Hypertrophic+"
223	TI (hypertrophic N1 cardiomyopath*) OR AB (hypertrophic N1 cardiomyopath*)
224	MH "Cardiomyopathy, Dilated" AND (TI (congen*) OR AB (congen*))
225	TI (congenital N3 cardiomyopath*) OR AB (congenital N3 cardiomyopath*)
226	TI (restrictive cardiomyopath* or obliterative cardiomyopath* or constrictive cardiomyopath*) OR AB (restrictive cardiomyopath* or obliterative cardiomyopath* or constrictive cardiomyopath*)
227	MH "Pulmonary Fibrosis+"
228	TI (pulmonary fibros* or lung fibros* or bronchopulmonary fibros* or fibrosing alveolit* or interstitial pneumonit*) OR AB (pulmonary fibros* or lung fibros* or bronchopulmonary fibros* or fibrosing alveolit* or interstitial pneumonit*)
229	MH "Respiratory Failure"
230	TI (respiratory N1 (failure* or insufficienc*)) OR AB (respiratory N1 (failure* or insufficienc*))
231	MH "Cystic Adenomatoid Malformation of Lung, Congenital"

232	TI ((cystic lung or cystic pulmonary or cystic bronchopulmonary) N1 (disease* or disorder or syndrome*)) OR AB ((cystic lung or cystic pulmonary or cystic bronchopulmonary) N1 (disease* or disorder or syndrome*))
233	TI (bronchogenic cyst* or bronchopulmonary foregut malformation*) OR AB (bronchogenic cyst* or bronchopulmonary foregut malformation*)
234	TI (cystic adenomatoid malformation*) OR AB (cystic adenomatoid malformation*)
235	TI (lobar emphysem*) OR AB (lobar emphysem*)
236	TI (pulmonary sequestration* or bronchopulmonary sequestration* or lung sequestration* or extralobar sequestration* or extra-lobar sequestration* or intralobar sequestration* or intra-lobar sequestration*) OR AB (pulmonary sequestration* or bronchopulmonary sequestration* or lung sequestration* or extralobar sequestration* or extra-lobar sequestration* or intralobar sequestration* or intra-lobar sequestration*)
237	TI (pulmolithias*) OR AB (pulmolithias*)
238	MH "Liver Failure+"
239	TI ((liver# or hepatic) N3 fail*) OR AB ((liver# or hepatic) N3 fail*)
240	MH "Liver Cirrhosis+"
241	TI (cirrhosis N3 liver#) OR AB (cirrhosis N3 liver#)
242	MH "Sinusoidal Obstruction Syndrome"
243	TI ((veno-occlusive or venous occlusive) N1 (disease* or syndrome* or disorder*)) OR AB ((veno-occlusive or venous occlusive) N1 (disease* or syndrome* or disorder*))
244	MH "Exocrine Pancreatic Insufficiency"
245	TI (swachman-diamond or shwachman-bodian or schwachmann-diamond or shwachmann-bodian) OR AB (swachman-diamond or shwachman-bodian or schwachmann-diamond or shwachmann-bodian)
246	MH "Wegener's Granulomatosis"
247	TI (wegener* granulomatos*) OR AB (wegener* granulomatos*)
248	TI (granulomatos* N3 polyangiit*) OR AB (granulomatos* N3 polyangiit*)
249	MH "Osteolysis, Essential"
250	TI (essential osteolys*) OR AB (essential osteolys*)
251	TI ((gorham* or gorham-stout* or vanishing bone or phantom bone) N1 (disease* or syndrome* or disorder)) OR AB ((gorham* or gorham-stout* or vanishing bone or phantom bone) N1 (disease* or syndrome* or disorder))
252	TI ((arc or arthrogryposis renal dysfunction cholestasis) N1 (disease* or syndrome* or disorder)) OR AB ((arc or arthrogryposis renal dysfunction cholestasis) N1 (disease* or syndrome* or disorder))
253	MH "Cerebral Hemorrhage" AND (TI (congen*) OR AB (congen*))
254	MH "Cerebral Hemorrhage" AND TI ((trauma*) OR AB (trauma*))
255	MH "Cerebral Hemorrhage" AND MH "Birth Injuries"
256	TI (cerebral h#emorrhage* and (birth* N3 injur*)) OR AB (cerebral h#emorrhage* and (birth* N3 injur*))
257	MH "Asphyxia Neonatorum"
258	TI (asphyxia neonatorum) OR AB (asphyxia neonatorum)
259	TI ((perinatal* or neonatal* or birth*) N3 asphyxia*) OR AB ((perinatal* or neonatal* or birth*) N3 asphyxia*)
260	MH "Rubella Syndrome, Congenital"
261	TI (congenital rubella) OR AB (congenital rubella)

262	MH "Cytomegalovirus Infections" AND (TI (congen*) OR AB (congen*))
263	TI (congenital N1 (cytomegalovirus* or cmv)) OR AB (congenital N1 (cytomegalovirus* or cmv))
264	MH "Chickenpox" AND (TI congen* or AB congen*)
265	MH "Herpes Zoster+" AND (TI congen* or AB congen*)
266	TI ((congenital or fetal or foetal) N3 (varicella* or chicken pox* or VZV)) Or AB ((congenital or fetal or foetal) N3 (varicella* or chicken pox* or VZV))
267	TI (congenital toxoplasmos*) OR AB (congenital toxoplasmos*)
268	MH "Hypoxia, Brain+"
269	TI ((brain* or cerebral) N3 hypoxi*) OR AB ((brain* or cerebral) N3 hypoxi*)
270	MH "Renal Insufficiency" AND (TI (congen*) OR AB (congen*))
271	MH "Kidney Failure, Acute" AND (TI (congen*) OR AB (congen*))
272	MH "Kidney Failure, Chronic" AND (TI (congen*) OR AB (congen*))
273	MH "Renal Insufficiency, Chronic" AND (TI (congen*) OR AB (congen*))
274	TI (congenital* N3 (kidney failure* or renal failure* or kidney insufficienc* or renal insufficienc*)) OR AB (congenital* N3 (kidney failure* or renal failure* or kidney insufficienc* or renal insufficienc*))
275	TI (congenital* N3 (kidney disease* or renal disease*)) OR AB (congenital* N3 (kidney disease* or renal disease*))
276	MH "Anencephaly"
277	TI (anencephal* or meroanencephal* or craniorachischis*) Or AB (anencephal* or meroanencephal* or craniorachischis*)
278	TI (aprosencephal* N3 open cranium) OR AB (aprosencephal* N3 open cranium)
279	TI (encephalocele* or cranium bifidum) OR AB (encephalocele* or cranium bifidum)
280	TI (dandy-walker*) OR AB (dandy-walker*)
281	MH "Acrocallosal Syndrome"
282	TI (acrocallosal or acro-callosal or acrocolossal or acro colossal) OR AB (acrocallosal or acro-callosal or acrocolossal or acro colossal)
283	MH "Aicardi Syndrome"
284	TI (aicardi* N1 (syndrome* or disease* or disorder*)) OR AB (aicardi* N1 (syndrome* or disease* or disorder*))
285	TI (holoprosencephal* or arhinencephal* or holosprosencephal*) OR AB (holoprosencephal* or arhinencephal* or holosprosencephal*)
286	TI (hydranencephal* or hydrancephal* or hydroanencephal*) OR AB (hydranencephal* or hydrancephal* or hydroanencephal*)
287	MH "Lissencephaly+"
288	MH "Microcephaly"
289	TI (lissencephal* or walker-warburg* or miller-dieker* or norman-robert* or microlissencephal*) OR AB (lissencephal* or walker-warburg* or miller-dieker* or norman-robert* or microlissencephal*)
290	TI ((fukuyama* or muscle-eye-brain) N1 (syndrome* or disease* or disorder*)) OR AB ((fukuyama* or muscle-eye-brain) N1 (syndrome* or disease* or disorder*))
291	MH "Malformations of Cortical Development"
292	TI (microgyria* or microgyrus or micro-gyria* or micro-gyrus) OR AB (microgyria* or microgyrus or micro-gyria* or micro-gyrus)
293	TI (pachygyria* or pachgyria*) OR AB (pachygyria* or pachgyria*)

294	TI (agyria*) OR AB (agyria*)
295	TI ((septo-optic or septooptic) N1 dysplas*) OR AB ((septo-optic or septooptic) N1 dysplas*)
296	TI (de morsier*) OR AB (de morsier*)
297	TI (schizencephal* or schizzencephal*) OR AB (schizencephal* or schizzencephal*)
298	MH "Arnold-Chiari Malformation"
299	TI (chiari* malformation*) OR AB (chiari* malformation*)
300	MH "Truncus Arteriosus, Persistent"
301	TI (truncus or common arterial trunk*) OR AB (truncus or common arterial trunk*)
302	MH "Transposition of Great Arteries"
303	TI ((transposition* or dextrotransposition* or dtransposition* or levotransposition* or ltransposition*) N3 (great arter* or main arter* or aorta* or pulmonary arter* or great vessel* or main vessel*)) OR AB ((transposition* or dextrotransposition* or dtransposition* or levotransposition* or ltransposition*) N3 (great arter* or main arter* or aorta* or pulmonary arter* or great vessel* or main vessel*))
304	TI (dextro-tga or d-tga or levo-tga or l-tga) OR AB (dextro-tga or d-tga or levo-tga or l-tga)
305	TI (double inlet N3 ventricle*) OR AB (double inlet N3 ventricle*)
306	TI (DILV) OR AB (DILV)
307	TI (single ventricle*) OR AB (single ventricle*)
308	MH "Heart Defects, Congenital" AND "Atrial Appendage"
309	TI (isomerism N3 atrial appendage*) OR AB (isomerism N3 atrial appendage*)
310	TI (aspleni* or polyspleni* or poly-spleni*) OR AB (aspleni* or polyspleni* or poly-spleni*)
311	MH "Tetralogy of Fallot"
312	TI (tetralogy N3 fallot*) OR AB (tetralogy N3 fallot*)
313	MH "Eisenmenger Complex"
314	TI (eisenmenger* or tardive cyanos* or eisenmeyer*) OR AB (eisenmenger* or tardive cyanos* or eisenmeyer*)
315	TI (pentalogy N3 fallot*) OR AB (pentalogy N3 fallot*)
316	MH "Pulmonary Atresia"
317	TI ((pulmonary or bronchopulmonary or lung*) N3 atresia*) OR AB ((pulmonary or bronchopulmonary or lung*) N3 atresia*)
318	MH "Tricuspid Atresia"
319	TI ((tricuspid or tri) N3 atresia*) OR AB ((tricuspid or tri) N3 atresia*)
320	MH "Ebstein's Anomaly"
321	TI (ebstein* N (anomal* or malformation*)) OR AB (ebstein* N (anomal* or malformation*))
322	MH "Hypoplastic Left Heart Syndrome"
323	TI (hypoplastic left heart N1 (syndrome* or disease* or disorder*)) OR AB (hypoplastic left heart N1 (syndrome* or disease* or disorder*))
324	TI ((aortic or aorta*) N3 atresia*) OR AB ((aortic or aorta*) N3 atresia*)
325	TI (mitral N3 atresia*) OR AB (mitral N3 atresia*)
326	TI ((absence* or absent*) N3 (aorta* or aortic)) OR AB ((absence* or absent*) N3 (aorta* or aortic))
327	TI (aplas* N3 (aorta* or aortic)) OR AB (aplas* N3 (aorta* or aortic))

328	MH "Aortic Aneurysm+" AND (TI (congen*) OR AB (congen*))
329	TI (((aorta* or aortic) N3 aneurys*) and congenital*) OR AB (((aorta* or aortic) N3 aneurys*) and congenital*)
330	TI (hypoplas* N3 (aorta* or aortic)) OR AB (hypoplas* N3 (aorta* or aortic))
331	TI (convulsion* N3 (aorta* or aortic)) OR AB (convulsion* N3 (aorta* or aortic))
332	TI (persistent right N3 (aorta* or aortic)) OR AB (persistent right N3 (aorta* or aortic))
333	TI ((anomalous pulmonary venous or anamolous pulmonary venous) N1 (connection or drainage or return)) OR AB ((anomalous pulmonary venous or anamolous pulmonary venous) N1 (connection or drainage or return))
334	TI ((absence* or absent*) N3 vena* cava*) OR AB ((absence* or absent*) N3 vena* cava*)
335	TI (persistent left N3 cardinal vein*) OR AB (persistent left N3 cardinal vein*)
336	MH "Scimitar Syndrome"
337	TI ((scimitar* or pulmonary venolobar) N1 (syndrome* or disease* or disorder*)) OR AB ((scimitar* or pulmonary venolobar) N1 (syndrome* or disease* or disorder*))
338	(MH "arteriovenous malformations" OR MH "intracranial arteriovenous malformations") AND (TI (bilateral) OR AB (bilateral))
339	TI ((bilateral AV or bilateral arteriovenous or bilateral arterio-venous) N3 malform*) OR AB ((bilateral AV or bilateral arteriovenous or bilateral arterio-venous) N3 malform*)
340	TI ((trachea* or windpipe* or wind-pipe*) N3 atresia*) OR AB ((trachea* or windpipe* or wind-pipe*) N3 atresia*)
341	TI ((trachea* or laryngotrachea* or glottic or subglottic or sub-glottic) N3 stenosis) OR AB ((trachea* or laryngotrachea* or glottic or subglottic or sub-glottic) N3 stenosis)
342	MH "Bronchopulmonary Dysplasia"
343	TI ((lung* or pulmonary or bronchopulmonary) N3 (hypoplas* or dysplas*)) OR AB ((lung* or pulmonary or bronchopulmonary) N3 (hypoplas* or dysplas*))
344	TI ((absence* or absent*) N3 (esophag* or oesophag* or foodpipe or food-pipe* or gullet*)) OR AB ((absence* or absent*) N3 (esophag* or oesophag* or foodpipe or food-pipe* or gullet*))
345	TI (duoden* N3 atresia*) OR AB (duoden* N3 atresia*)
346	TI ((absence* or absent*) N3 (intestin* or gastrointestin*)) OR AB ((absence* or absent*) N3 (intestin* or gastrointestin*))
347	TI ((intestin* or gastrointestin*) N3 atresia*) OR AB ((intestin* or gastrointestin*) N3 atresia*)
348	TI ((intestin* or gastrointestin*) N3 stenos*) OR AB ((intestin* or gastrointestin*) N3 stenos*)
349	TI (cloaca* N3 (abnor* or malform* or anomal*)) OR AB (cloaca* N3 (abnor* or malform* or anomal*))
350	TI (cloaca* N3 exopthalmo*) OR AB (cloaca* N3 exopthalmo*)
351	MH "Biliary Atresia"
352	TI (biliary N3 atresia*) OR AB (biliary N3 atresia*)
353	TI (extrahepatic ductopen* or extra-hepatic ductopen* or progressive obliterative cholangiopath*) OR AB (extrahepatic ductopen* or extra-hepatic ductopen* or progressive obliterative cholangiopath*)

354	TI (biliary N3 hypoplas*) OR AB (biliary N3 hypoplas*)
355	TI (alagille* N3 atresia*) OR AB (alagille* N3 atresia*)
356	TI ((absence* or absent*) N3 kidney*) OR AB ((absence* or absent*) N3 kidney*)
357	TI (potter* N1 (sequence* or syndrome* or disease* or disorder*)) OR AB (potter* N1 (sequence* or syndrome* or disease* or disorder*))
358	MH "Oligohydramnios"
359	TI (oligohydramn*) OR AB (oligohydramn*)
360	MH "Multicystic Dysplastic Kidney"
361	TI ((kidney* or renal) N3 dysplas*) OR AB ((kidney* or renal) N3 dysplas*)
362	TI ((meckel* or meckelgruber* or gruber*) N1 (syndrome* or disease* or disorder*)) OR AB ((meckel* or meckelgruber* or gruber*) N1 (syndrome* or disease* or disorder*))
363	TI (dysencephalia splanchnocystica*) OR AB (dysencephalia splanchnocystica*)
364	TI (pena-shokeir* or penn-shokeir*) OR AB (pena-shokeir* or penn-shokeir*)
365	TI (larsen* N1 (syndrome* or disease* or disorder*)) OR AB (larsen* N1 (syndrome* or disease* or disorder*))
366	MH "Acrocephalosyndactylia"
367	TI (acrocephalosyndactyl*) OR AB (acrocephalosyndactyl*)
368	TI (pfeiffer* N1 (syndrome* or disease* or syndrome*)) OR AB (pfeiffer* N1 (syndrome* or disease* or syndrome*))
369	MH "Short-Rib Polydactyly Syndrome"
370	TI (short rib#) OR AB (short rib#)
371	TI (saldino-noonan* or majewski* or verma-naumoff* or beemer-langer*) OR AB (saldino-noonan* or majewski* or verma-naumoff* or beemer-langer*)
372	TI (jeune* N1 (syndrome* or disease* or disorder*)) OR AB (jeune* N1 (syndrome* or disease* or disorder*))
373	TI (asphyxiating thoracic dysplas*) OR AB (asphyxiating thoracic dysplas*)
374	TI (chondrodysplasia punctata*) OR AB (chondrodysplasia punctata*)
375	TI ((conradi* or h#nermann* or happle*) N3 (syndrome* or disease* or disorder*)) OR AB ((conradi* or h#nermann* or happle*) N3 (syndrome* or disease* or disorder*))
376	MH "Osteogenesis Imperfecta"
377	TI (osteogenesis imperfecta) OR AB (osteogenesis imperfecta)
378	TI ((brittle bone or lobstein*) N1 (disease* or disorder* or syndrome*)) OR AB ((brittle bone or lobstein*) N1 (disease* or disorder* or syndrome*))
379	MH "Osteochondrodysplasias"
380	TI (spondyloepimetaphyseal or spondyloepiphyseal or spendylo metaphyseal) OR AB (spondyloepimetaphyseal or spondyloepiphyseal or spendylo metaphyseal)
381	MH "Hernia, Umbilical"
382	TI (omphalocele* or omphalocoele* or exomphalos) OR AB (omphalocele* or omphalocoele* or exomphalos)
383	TI (hernia* N3 umbilic*) OR AB (hernia* N3 umbilic*)
384	MH "Gastroschisis"
385	TI (gastroschis*) OR AB (gastroschis*)
386	TI (lamellar* N3 ichthyos*) OR AB (lamellar* N3 ichthyos*)

387	TI ((harlequin* or harloquin*) N3 (ichthyos* or baby or babies or f#etus*)) OR AB ((harlequin* or harloquin*) N3 (ichthyos* or baby or babies or f#etus*))
388	TI (ichthyosis congenita* or ichthyosis fetalis or keratosis diffusa fetalis) OR AB (ichthyosis congenita* or ichthyosis fetalis or keratosis diffusa fetalis)
389	MH "Epidermolysis Bullosa+"
390	TI (epidermolysis bullosa*) OR AB (epidermolysis bullosa*)
391	TI (johanson-blizzard* or johanna-blizzard*) OR AB (johanson-blizzard* or johanna-blizzard*)
392	MH "Xeroderma Pigmentosum"
393	TI (xeroderma pigmentosum) OR AB (xeroderma pigmentosum)
394	MH "Ectodermal Dysplasia"
395	TI (lacrimo-auriculo-dento-digital) OR AB (lacrimo-auriculo-dento-digital)
396	TI (ectodermal dysplas*) OR AB (ectodermal dysplas*)
397	TI ((ladd or eec) N1 (syndrome* or disease* or disorder*)) Or AB ((ladd or eec) N1 (syndrome* or disease* or disorder*))
398	MH "Sturge-Weber Syndrome"
399	TI (sturge-weber or encephalotrigeminal angiomatos*) OR AB (sturge-weber or encephalotrigeminal angiomatos*)
400	MH "Fetal Alcohol Syndrome"
401	TI (f#etal alcohol) OR AB (f#etal alcohol)
402	MH "Pierre Robin Syndrome"
403	TI (pierre robin*) OR AB (pierre robin*)
404	MH "Acrocephalosyndactylia"
405	TI (acrocephalosyndact* or acrocephalopolysyndact*) OR AB (acrocephalosyndact* or acrocephalopolysyndact*)
406	TI ((apert* or crouzon* or saethre-chotzen* or noack* or carpenter* or sakati-nyhan-tisdale* or goodman*) N1 (syndrome* or disorder* or disease*)) OR AB ((apert* or crouzon* or saethre-chotzen* or noack* or carpenter* or sakati-nyhan-tisdale* or goodman*) N1 (syndrome* or disorder* or disease*))
407	TI (fraser* N1 (syndrome* or disease* or disorder*)) OR AB (fraser* N1 (syndrome* or disease* or disorder*))
408	TI (cryptophthalmos) OR AB (cryptophthalmos)
409	TI (cyclopia# or cyclocephal* or synophthalmi*) Or AB (cyclopia# or cyclocephal* or synophthalmi*)
410	MH "Goldenhar Syndrome"
411	TI (goldenhar* or oculo-auriculo-vertebral) OR AB (goldenhar* or oculo-auriculo-vertebral)
412	MH "Mobius Syndrome"
413	TI ((m#bius* or moebius*) N1 (syndrome* or disease* or disorder*)) OR AB ((m#bius* or moebius*) N1 (syndrome* or disease* or disorder*))
414	MH "Orofaciodigital Syndromes"
415	TI (orofaciodigital or oro-facial-digital or oral-facial-digital or papillon-league* or psaume*) OR AB (orofaciodigital or oro-facial-digital or oral-facial-digital or papillon-league* or psaume*)
416	TI (robin* N1 (syndrome* or disorder* or disease*)) OR AB (robin* N1 (syndrome* or disorder* or disease*))

417	TI (freeman-sheldon* or distal arthrogrypos* or craniocarpotarsal dysplas* or craniocarpotarsal dystroph* or canio-carpo-tarsal or windmill-vane-hand* or whistling-face) OR AB (freeman-sheldon* or distal arthrogrypos* or craniocarpotarsal dysplas* or craniocarpotarsal dystroph* or canio-carpo-tarsal or windmill-vane-hand* or whistling-face)
418	MH "De Lange Syndrome"
419	TI ((de lange* or bushy*) N1 (syndrome* or disorder* or disease*)) OR AB ((de lange* or bushy*) N1 (syndrome* or disorder* or disease*))
420	TI (amsterdam dwarfism) OR AB (amsterdam dwarfism)
421	TI (aarskog or faciodigitogenital or facio-digito-genital or facial digital genital or shawl scrotum or faciogenital or facio-genital) OR AB (aarskog or faciodigitogenital or facio-digito-genital or facial digital genital or shawl scrotum or faciogenital or facio-genital)
422	MH "Cockayne Syndrome"
423	TI (cockayne* or neill-dingwall*) OR AB (cockayne* or neill-dingwall*)
424	TI (cerebro-oculo-facio-skeletal or cerebro-oculo-facial-skeletal) OR AB (cerebro-oculo-facio-skeletal or cerebro-oculo-facial-skeletal)
425	TI (dubowitz* N1 (syndrome* or disease* or disorder*)) OR AB (dubowitz* N1 (syndrome* or disease* or disorder*))
426	TI (robinow* or robinhow*) OR AB (robinow* or robinhow*)
427	TI (f#etal face or f#etal facies or f#etal faces or acral dysostos* or mesomelic dwarfism or covesdem*) OR AB (f#etal face or f#etal facies or f#etal faces or acral dysostos* or mesomelic dwarfism or covesdem*)
428	MH "Silver-Russell Syndrome"
429	TI (silver-russell* or russell-silver*) OR AB (silver-russell* or russell-silver*)
430	TI (silver* N1 (syndrome* or disease* or disorder*)) OR AB (silver* N1 (syndrome* or disease* or disorder*))
431	TI ((seckel* or harper*) N1 (syndrome* or disease* or disorder*)) OR AB ((seckel* or harper*) N1 (syndrome* or disease* or disorder*))
432	TI (microcephalic primordial dwarfism or bird-headed dwarf* or virchow-seckel dwarfism) OR AB (microcephalic primordial dwarfism or bird-headed dwarf* or virchow-seckel dwarfism)
433	MH "Smith-Lemli-Opitz Syndrome"
434	TI (smith-lemli-opitz* or dehydrocholesterol reductase deficien*) OR AB (smith-lemli-opitz* or dehydrocholesterol reductase deficien*)
435	MH "Prader-Willi Syndrome"
436	TI (prader-willi* or pradar-willi*) OR AB (prader-willi* or pradar-willi*)
437	MH "Rubinstein-Taybi Syndrome"
438	TI (rubinstein-taybi* or rubenstein-tabyii* or broad thumb-hallux) OR AB (rubinstein-taybi* or rubenstein-tabyii* or broad thumb-hallux)
439	TI ((rubinstein* or rubenstein*) N2 (syndrome* or disease* or disorder*)) OR AB ((rubinstein* or rubenstein*) N2 (syndrome* or disease* or disorder*))
440	MH "Nephritis, Hereditary"
441	TI (alport* N1 (syndrome* or disease* or disorder*)) OR AB (alport* N1 (syndrome* or disease* or disorder*))
442	TI (hereditary nephritis or h#emorrhagic familial nephritis) OR AB (hereditary nephritis or h#emorrhagic familial nephritis)

443	TI (hereditary deafness N3 nephropath*) OR AB (hereditary deafness N3 nephropath*)
444	TI (h#ematuria N3 nephropath* N3 deafness) OR AB (h#ematuria N3 nephropath* N3 deafness)
445	TI (laurence-moon*) OR AB (laurence-moon*)
446	MH "Bardet-Biedl Syndrome"
447	TI (bardet-biedl* or biedl-bardet*) OR AB (bardet-biedl* or biedl-bardet*)
448	MH "Zellweger Syndrome"
449	TI (zellweger*) OR AB (zellweger*)
450	TI ((cerebrohepatorenal or cerebro-hepato-renal) N1 (syndrome* or disease* or disorder*)) OR AB ((cerebrohepatorenal or cerebro-hepato-renal) N1 (syndrome* or disease* or disorder*))
451	TI (edward* N1 (syndrome* or disease* or disorder*)) OR AB (edward* N1 (syndrome* or disease* or disorder*))
452	TI ("trisomy 18") OR AB ("trisomy 18")
453	TI (patau* N1 (syndrome* or disease* or disorder*)) OR AB (patau* N1 (syndrome* or disease* or disorder*))
454	TI ("trisomy 13" or "trisomy D") OR AB ("trisomy 13" or "trisomy D")
455	TI ("trisomy 22") OR AB ("trisomy 22")
456	TI ("trisomy 9") OR AB ("trisomy 9")
457	TI ("trisomy 10") OR AB ("trisomy 10")
458	TI (duplication syndrome*) OR AB (duplication syndrome*)
459	TI (("chromosome 8" or "chr 8") N5 duplicat*) OR AB (("chromosome 8" or "chr 8") N5 duplicat*)
460	TI (("chromosome x" or "chr x") and duplicat*) OR AB (("chromosome x" or "chr x") and duplicat*)
461	TI (chromosom* abnormality N5 duplicat*) OR AB (chromosom* abnormality N5 duplicat*)
462	TI ("tetrasomy 5p") OR AB ("tetrasomy 5p")
463	TI (tetrasomy N3 mosaic*) OR AB (tetrasomy N3 mosaic*)
464	MH "Pallister-Killian Syndrome"
465	TI (delet* N5 short arm N5 "chrom* 4") OR AB (delet* N5 short arm N5 "chrom* 4")
466	TI ((wolf-hirschhorn* or wolff hirschorn* or chromosome deletion dillan* or pitt-rogers-dank* or pitt*) N3 (syndrome* or disease* or disorder*)) OR AB ((wolf-hirschhorn* or wolff hirschorn* or chromosome deletion dillan* or pitt-rogers-dank* or pitt*) N3 (syndrome* or disease* or disorder*))
467	MH "Cri-du-Chat Syndrome"
468	TI ((cri du chat* or crying cat* or 5p or lejeune*) N3 (syndrome* or disease* or disorder*)) OR AB ((cri du chat* or crying cat* or 5p or lejeune*) N3 (syndrome* or disease* or disorder*))
469	TI ((jacobsen* or 11q deletion) N5 (syndrome* or disease* or disorder*)) OR AB ((jacobsen* or 11q deletion) N5 (syndrome* or disease* or disorder*))
470	TI (9p minus or 9p deletion) OR AB (9p minus or 9p deletion)
471	TI (alfi* N1 (syndrome* or disease* or disorder*)) OR AB (alfi* N1 (syndrome* or disease* or disorder*))
472	TI (degouchy* or de gouchy* or degrouchy* or de grouchy*) OR AB (degouchy* or de gouchy* or degrouchy* or de grouchy*)

473	TI (distal 18q) OR AB (distal 18q)
474	MH "Hypoventilation" AND (TI (congen*) Or Ab (congen*))
475	TI (ondine* curse or congenital central hypoventilation or primary alveolar hypoventilation) OR AB (ondine* curse or congenital central hypoventilation or primary alveolar hypoventilation)
476	MH "Graft versus Host Disease" AND (MH "Chronic Disease" or TI (chronic*) OR AB (chronic*))
477	TI (((graft vs host or graft versus host) N1 (disease* or syndrome* or disorder)) and chronic*) OR AB (((graft vs host or graft versus host) N1 (disease* or syndrome* or disorder)) and chronic*)
478	MH "Human Immunodeficiency Virus+"
479	MH "HIV Infections+"
480	TI (HIV or human immunodeficiency virus*) OR AB (HIV or human immunodeficiency virus*)
481	TI (htlv or human t-lymphotropic virus* or human t cell lymphotropic virus*) OR AB (htlv or human t-lymphotropic virus* or human t cell lymphotropic virus*)
482	TI (acquired immune deficiency syndrome* or acquired immunodeficiency syndrome*) OR AB (acquired immune deficiency syndrome* or acquired immunodeficiency syndrome*)
483	TI (AIDS N3 (virus* or infection*)) OR AB (AIDS N3 (virus* or infection*))
484	TI (AIDS N3 (virus* or infection*)) OR AB (AIDS N3 (virus* or infection*))
485	MH "Neoplasms+"
486	TI (cancer* or carcin* or tumor* or tumour* or neoplas* or adenocarcin* or oncol* or malignan*) OR AB (cancer* or carcin* or tumor* or tumour* or neoplas* or adenocarcin* or oncol* or malignan*)
487	MH "Cystic Fibrosis"
488	TI (cystic fibrosis or fibrocystic or fibro-cystic or mucoviscidosis or cf) OR AB (cystic fibrosis or fibrocystic or fibro-cystic or mucoviscidosis or cf)
489	MH "Cerebral Palsy"
490	TI (cerebr* N3 pals*) OR AB (cerebr* N3 pals*)
491	MH "Muscle Spasticity"
492	TI (spasticit*) OR AB (spasticit*)
493	MH "Quadriplegia"
494	TI (spastic* and (quadripleg* or tetrapleg*)) OR AB (spastic* and (quadripleg* or tetrapleg*))
495	MH "Renal Insufficiency+"
496	TI ((kidney* or renal) N3 (failure* or insufficienc*)) OR AB ((kidney* or renal) N3 (failure* or insufficienc*))
497	TI (end stage N3 (kidney or renal)) OR AB (end stage N3 (kidney or renal))
498	TI (("stage 5" or "stage V") N3 (kidney or renal)) OR AB (("stage 5" or "stage V") N3 (kidney or renal))
499	TI (ESRD or ESKD or ESRF or ESKF or CRF or CKF) OR AB (ESRD or ESKD or ESRF or ESKF or CRF or CKF)

500	S2 OR S3 OR S4 OR S5 OR S6 OR S7 OR S8 OR S9 OR S10 OR S11 OR S12 OR S13 OR S14 OR S15 OR S16 OR S17 OR S18 OR S19 OR S20 OR S21 OR S22 OR S23 OR S24 OR S25 OR S26 OR S27 OR S28 OR S29 OR S30 OR S31 OR S32 OR S33 OR S34 OR S35 OR S36 OR S37 OR S38 OR S39 OR S40 OR S41 OR S42 OR S43 OR S44 OR S45 OR S46 OR S47 OR S48 OR S49 OR S50 OR S51 OR S52 OR S53 OR S54 OR S55 OR S56 OR S57 OR S58 OR S59 OR S60 OR S61 OR S62 OR S63 OR S64 OR S65 OR S66 OR S67 OR S68 OR S69 OR S70 OR S71 OR S72 OR S73 OR S74 OR S75 OR S76 OR S77 OR S78 OR S79 OR S80 OR S81 OR S82 OR S83 OR S84 OR S85 OR S86 OR S87 OR S88 OR S89 OR S90 OR S91 OR S92 OR S93 OR S94 OR S95 OR S96 OR S97 OR S98 OR S99 OR S100 OR S101 OR S102 OR S103 OR S104 OR S105 OR S106 OR S107 OR S108 OR S109 OR S110 OR S111 OR S112 OR S113 OR S114 OR S115 OR S116 OR S117 OR S118 OR S119 OR S120 OR S121 OR S122 OR S123 OR S124 OR S125 OR S126 OR S127 OR S128 OR S129 OR S130 OR S131 OR S132 OR S133 OR S134 OR S135 OR S136 OR S137 OR S138 OR S139 OR S140 OR S141 OR S142 OR S143 OR S144 OR S145 OR S146 OR S147 OR S148 OR S149 OR S150 OR S151 OR S152 OR S153 OR S154 OR S155 OR S156 OR S157 OR S158 OR S159 OR S160 OR S161 OR S162 OR S163 OR S164 OR S165 OR S166 OR S167 OR S168 OR S169 OR S170 OR S171 OR S172 OR S173 OR S174 OR S175 OR S176 OR S177 OR S178 OR S179 OR S180 OR S181 OR S182 OR S183 OR S184 OR S185 OR S186 OR S187 OR S188 OR S189 OR S190 OR S191 OR S192 OR S193 OR S194 OR S195 OR S196 OR S197 OR S198 OR S199 OR S200 OR S201 OR S202 OR S203 OR S204 OR S205 OR S206 OR S207 OR S208 OR S209 OR S210 OR S211 OR S212 OR S213 OR S214 OR S215 OR S216 OR S217 OR S218 OR S219 OR S220 OR S221 OR S222 OR S223 OR S224 OR S225 OR S226 OR S227 OR S228 OR S229 OR S230 OR S231 OR S232 OR S233 OR S234 OR S235 OR S236 OR S237 OR S238 OR S239 OR S240 OR S241 OR S242 OR S243 OR S244 OR S245 OR S246 OR S247 OR S248 OR S249 OR S250 OR S251 OR S252 OR S253 OR S254 OR S255 OR S256 OR S257 OR S258 OR S259 OR S260 OR S261 OR S262 OR S263 OR S264 OR S265 OR S266 OR S267 OR S268 OR S269 OR S270 OR S271 OR S272 OR S273 OR S274 OR S275 OR S276 OR S277 OR S278 OR S279 OR S280 OR S281 OR S282 OR S283 OR S284 OR S285 OR S286 OR S287 OR S288 OR S289 OR S290 OR S291 OR S292 OR S293 OR S294 OR S295 OR S296 OR S297 OR S298 OR S299 OR S300 OR S301 OR S302 OR S303 OR S304 OR S305 OR S306 OR S307 OR S308 OR S309 OR S310 OR S311 OR S312 OR S313 OR S314 OR S315 OR S316 OR S317 OR S318 OR S319 OR S320 OR S321 OR S322 OR S323 OR S324 OR S325 OR S326 OR S327 OR S328 OR S329 OR S330 OR S331 OR S332 OR S333 OR S334 OR S335 OR S336 OR S337 OR S338 OR S339 OR S340 OR S341 OR S342 OR S343 OR S344 OR S345 OR S346 OR S347 OR S348 OR S349 OR S350 OR S351 OR S352 OR S353 OR S354 OR S355 OR S356 OR S357 OR S358 OR S359 OR S360 OR S361 OR S362 OR S363 OR S364 OR S365 OR S366 OR S367 OR S368 OR S369 OR S370 OR S371 OR S372 OR S373 OR S374 OR S375 OR S376 OR S377 OR S378 OR S379 OR S380 OR S381 OR S382 OR S383 OR S384 OR S385 OR S386 OR S387 OR S388 OR S389 OR S390 OR S391 OR S392 OR S393 OR S394 OR S395 OR S396 OR S397 OR S398 OR S399 OR S400 OR S401 OR S402 OR S403 OR S404 OR S405 OR S406 OR S407 OR S408 OR S409 OR S410 OR S411 OR S412 OR S413 OR S414 OR S415 OR S416 OR S417 OR S418 OR S419 OR S420 OR S421 OR S422 OR S423 OR S424 OR S425 OR S426 OR S427 OR S428 OR S429 OR S430 OR S431 OR S432 OR S433 OR S434 OR S435 OR S436 OR S437 OR S438 OR S439 OR S440 OR S441 OR S442 OR S443 OR S444 OR S445 OR S446 OR S447 OR S448 OR S449 OR S450 OR S451 OR S452 OR

	S453 OR S454 OR S455 OR S456 OR S457 OR S458 OR S459 OR S460 OR S461 OR S462 OR S463 OR S464 OR S465 OR S466 OR S467 OR S468 OR S469 OR S470 OR S471 OR S472 OR S473 OR S474 OR S475 OR S476 OR S477 OR S478 OR S479 OR S480 OR S481 OR S482 OR S483 OR S484 OR S485 OR S486 OR S487 OR S488 OR S489 OR S490 OR S491 OR S492 OR S493 OR S494 OR S495 OR S496 OR S497 OR S498 OR S499 OR S500
501	MH "Terminally Ill Patients"
502	MH "Terminal Care"
503	MH "Palliative Care"
504	MH "Hospices" OR MH "Hospice Care"
505	TI (life N2 limit*) OR AB (life N2 limit*)
506	TI (life N2 threaten*) OR AB (life N2 threaten*)
507	TI (end of life) OR AB (end of life)
508	TI (eol) OR AB (eol)
509	TI (terminal* N2 (ill or illness* or condition# or disease# or syndrome* or disorder*)) OR AB (terminal* N2 (ill or illness* or condition# or disease# or syndrome* or disorder*))
510	TI (terminal N2 (care* or caring)) OR AB (terminal N2 (care* or caring))
511	TI (palliat*) Or AB (palliat*)
512	TI (care N2 dying) OR AB (care N2 dying)
513	TI (technology N2 dependent) OR AB (technology N2 dependent)
514	TI (hospice*) OR AB (hospice*)
515	MH "Rare Diseases"
516	MH "Metabolic Diseases"
517	TI (severe N2 (need or needs or illness* or disease# or disabilit* or impairment# or impediment# or condition# or disadvant* or problem# or syndrome# or disorder#)) OR AB (severe N2 (need or needs or illness* or disease# or disabilit* or impairment# or impediment# or condition# or disadvant* or problem# or syndrome# or disorder#))
518	TI (complex N2 (need or needs or illness* or disease# or disabilit* or impairment# or impediment# or condition# or disadvant* or problem# or syndrome# or disorder#)) OR AB (complex N2 (need or needs or illness* or disease# or disabilit* or impairment# or impediment# or condition# or disadvant* or problem# or syndrome# or disorder#))
519	TI (rare N2 (illness* or disease* or disabilit* or impairment* or impediment* or condition# or syndrome# or disorder#)) OR AB (rare N2 (illness* or disease* or disabilit* or impairment* or impediment* or condition# or syndrome# or disorder#))
520	TI (multiple N2 (need or needs or illness* or disease# or disabilit* or impairment# or impediment* or condition# or disadvant* or health or syndrome# or disorder#)) OR AB (multiple N2 (need or needs or illness* or disease# or disabilit* or impairment# or impediment* or condition# or disadvant* or health or syndrome# or disorder#))
521	TI (profound N2 (need or needs or illness* or disease* or disabilit* or impairment* or impediment* or condition# or syndrome# or disorder#)) OR AB (profound N2 (need or needs or illness* or disease* or disabilit* or impairment* or impediment* or condition# or syndrome# or disorder#))

522	TI (intense N2 (need or needs or illness* or disease* or disabilit* or impairment* or impediment* or condition# or syndrome# or disorder#)) OR AB (intense N2 (need or needs or illness* or disease* or disabilit* or impairment* or impediment* or condition# or syndrome# or disorder#))
523	TI (serious N2 (disabilit* or impairment* or impediment* or condition# or disadvant*)) OR AB (serious N2 (disabilit* or impairment* or impediment* or condition# or disadvant*))
524	S502 OR S503 OR S504 OR S505 OR S506 OR S507 OR S508 OR S509 OR S510 OR S511 OR S512 OR S513 OR S514 OR S515 OR S516 OR S517 OR S518 OR S519 OR S520 OR S521 OR S522 OR S523 OR S524
525	S501 OR S525
526	TI ((Young N1 people*) or Youth* or Care leaver* or residential child* or Adolescen* or Young adult* or Young person* or Young men* or Young women* or Teen* or juvenile* or Younger people or Youngster* or Looked after or Child welfare or paediatric* or pediatric* or peadiatric* or Young male* or Young female* or juvenile or children* or child or childhood or (young N1 patient*) or young carer* or minors or puber* or pubescen* or ((secondary or high*) N2 (school* or education))) OR AB ((Young N1 people*) or Youth* or Care leaver* or residential child* or Adolescen* or Young adult* or Young person* or Young men* or Young women* or Teen* or juvenile* or Younger people or Youngster* or Looked after or Child welfare or paediatric* or pediatric* or peadiatric* or Young male* or Young female* or juvenile or children* or child or childhood or (young N1 patient*) or young carer* or minors or puber* or pubescen* or ((secondary or high*) N2 (school* or education)))
527	MH "infant+"
528	MH "Child"
529	S529 NOT S528
530	MH "Child, disabled"
531	MH " Young Adult+"
532	MH "Adolescent, Hospitalized"
533	MH "Child, Institutionalized"
534	MH "Child, Hospitalized"
535	MH "Adolescence+"
536	S527 OR S528 OR S529 OR S530 OR S531 OR S532 OR S533 OR S534 OR S535 OR S536
537	TI ((transition* or transfer* or handoff or handover or hand over) and (Service* or care or clinic* or healthcare or hospital* or center* or centre* or facility or facilities or unit* or department* or institution* or agency or agencies or hospice* or provider* or program* or Coordinat* or Framework* or Managing or Managed or preparedness or Planning or Preparing or Preparation* or Plan* or Protocol* or planned or Support or Supporting or Trajectory or Trajectories or Pathway* or Process or Processes or Readiness or Partnership* or programme* or program* or training or strateg* or Failure* or Barrier* or system#))

538	AB ((transition* or transfer* or handoff or handover or hand over) N3 (Service* or care or clinic* or healthcare or hospital* or center* or centre* or facility or facilities or unit* or department* or institution* or agency or agencies or hospice* or provider* or program* or Coordinat* or Framework* or Managing or Managed or preparedness or Planning or Preparing or Preparation* or Plan* or Protocol* or planned or Support or Supporting or Trajectory or Trajectories or Pathway* or Process or Processes or Readiness or Partnership* or programme* or program* or training or strateg* or Failure* or Barrier* or system#))
539	TI (continu* and (care or healthcare or Support or Supporting or Failure* or Barrier*))
540	AB (continu* N3 (care or healthcare or Support or Supporting or Failure* or Barrier*)
541	MH "Transitional care"
542	MH "continuity of patient care"
543	MH "Patient Care Plans"
544	TI (transition* or transfer* or handoff or handover or hand* over) OR AB (transition* or transfer* or handoff or handover or hand* over)
545	(S543 OR S544) AND S545
546	MH "Transfer, Discharge"
547	S538 OR S539 OR S540 OR S541 OR S542 OR S546 OR S547
548	S526 AND S537 AND S548
549	PT (letter or editorial or comment or news)
550	S549 NOT S550

Social Sciences Citation Index (Web of Science)

Concepts:

1. LLC: lines 1-583
2. Child/young adult: lines 584-595
3. Transition: lines 596-607

1	Creutzfeldt-Jakob Syndrome
2	(creutzfeldt-jakob* or jakob-creutzfeldt* or cjd or spongiform encephalopath*)
4	(subacute sclerosing panencephalit* or sub-acute sclerosing panencephalit* or sspe or subacute sclerosing leukoencephalit* or sub-acute sclerosing leukoencephalit* or van bogaert* leukoencephalit* or measles inclusion body encephalit* or mibe)
5	beta-Thalassemia
6	(beta NEAR/1 (thalass$emi* or thalas$emi*))
7	((thalass$emi* or thalas$emi*) NEAR/1 major)
8	Anemia, Aplastic
9	((hypoplastic or aplastic) NEAR/1 an$emi*)
10	(medullary NEAR/3 hypoplas*)
11	Neutropenia
12	((severe or chronic*) NEAR/3 neutropeni*)
13	immunologic deficiency syndromes or acquired immunodeficiency syndrome
14	(immun* deficiency NEAR/1 (syndrome* or disease* or disorder*))
15	(immunodeficiency NEAR/1 (syndrome* or disease* or disorder*))
16	DiGeorge Syndrome
17	(digeorge* or di george* or sedlackova* or opitz g-bbb or velocardiofacial or velo-cardiofacial or velo-cardio-facial or shprintzen* or ctaf)
18	((deletion or vcf or pharyngeal pouch or thymic aplasia or anomaly face) NEAR/1 (syndrome* or disease* or disorder*))
19	Common Variable Immunodeficiency
20	((common variable or late onset) NEAR/3 (immunodeficienc* or immune deficienc* or immunoglobulin deficienc* or hypogammaglobulin*))
21	acquired hypogammaglobulin*
22	Cryoglobulinemia
23	cryoglobulin$em*
24	Polyendocrinopathies, Autoimmune
25	((autoimmune or failure*) NEAR/3 (polyglandular* or polyendocrin*))
26	Progeria
27	(progeria or hutchinson-gilford*)
28	Tyrosinemias
29	tyrosin$em*
30	Maple Syrup Urine Disease
31	(maple syrup urine or msud)
32	branched chain
33	(bckd NEAR/5 (deficienc* or ketoacid* or keto-acid*))
34	hyperleucine-isoleucin*

35	Methylmalonic Acid
36	(methylmalonic acid$emi* or methylmalonic aciduri* or methyl malonic acid$emi* or methyl malonic aciduri*)
37	Propionic Acidemia
38	(propionic acid$em* or propionic acidur* or propionyl-CoA carboxylase deficienc* or ketotic glycin$em*)
39	Adrenoleukodystrophy
40	(adrenoleukodystroph* or x-ald or schilder-addison* or addison-schilder* or adrenomyeloneuropath*)
41	Carnitine O-Palmitoyltransferase
42	((carnitine palmityltransferase or carnitine palmitoyltransferase or carnitine o-palmityltransferase or carnitine o-palmitoyltransferase) NEAR/3 deficienc*)
43	Fanconi Syndrome
44	(fanconi* NEAR/1 (syndrome* or disease* or disorder*))
45	(ocular NEAR/3 (renal or kidney))
46	Cystinosis
47	(cystinos* or cystine storage or cystine diathes* or cystine disease*)
48	Oculocerebrorenal Syndrome
49	((lowe or lowes or oculocerebrorenal or cerebrooculorenal or cerebro-oculo-renal) NEAR/3 (syndrome* or disease* or disorder*))
50	Metalloproteinsdf
51	Molybdenumdf
52	(molybdenum cofactor deficien* or molybdenum co-factor deficien*)
53	Oxidoreductases Acting on Sulfur Group Donorsdf
54	Sulfite Oxidasedf
55	((sulphite* or sulfite*) NEAR/3 oxidase deficien*)
56	Argininosuccinic Acid
57	(argininosuccinic acid* or argininosuccinic acid$emi*)
58	Citrullinemia
59	(citrullin$emi* or citrullinuri*)
60	Amino Acid Metabolism, Inborn Errors
61	(glutaric acid$emi* or glutaric aciduri*)
62	Hyperglycinemia, Nonketotic
63	(glycine encephalopath* or non-ketotic hyperglycin$emi* or nonketotic hyperglycin$emi*)
64	Hyperargininemia
65	(arginin$emi* or arginase deficien* or hyperarginin$emi*)
66	Renal Aminoacidurias
67	(aminoaciduri* or aminoacid$emi*)
68	glycogen storage disease
69	(glycogen storage NEAR/1 (disease* or syndrome* or disorder*))
70	(pompe* NEAR/1 (disease* or syndrome* or disorder*))
71	Galactosemias
72	galactos$emi*
73	Pyruvate Dehydrogenase Complex Deficiency Disease
74	(pyruvate dehydrogenase NEAR/3 deficien*)
75	(oxalosis and (renal or kidney*))
76	Gangliosidoses
77	gangliosidos*
78	(sandhoff* NEAR/1 (disease* or syndrome* or disorder*))

79	tay sach*
80	Mucolipidoses
81	mucolipidos*
82	Canavan Disease
83	(canavan* leucodystroph* or aspartoacylase deficien* or aminoacylase 2 deficien*)
84	((canavan* or canavan-van bogaert-bertrand*) NEAR/1 (disease* or syndrome* or disorder*))
85	Gaucher Disease
86	(gaucher* NEAR/1 (disease* or syndrome* or disorder*))
87	(glucocerebrosidase deficien* or glucosylceramidase deficien*)
88	Leukodystrophy, Metachromatic
89	(metachromatic leukodystroph* or arylsulfatase A deficien* or metachromic leukodystroph*)
90	Niemann-Pick Diseases
91	(niemann-pick* or sphingomyelinase deficien*)
92	Sphingolipidoses
93	sphingolipidos*
94	Fabry Disease
95	(fabry* NEAR/1 (disease* or syndrome* or disorder*))
96	(angiokeratoma corporis diffusum or alpha-galactosidase A deficien*)
97	Leukodystrophy, Globoid Cell
98	(krabbe* NEAR/1 (disease* or syndrome* or disorder*))
99	(globoid cell leukodystroph* or galactosylceramide lipidos* or galactosylcerebrosidase deficien* or galactosylceramidase deficien*)
100	Farber Lipogranulomatosis
101	(farber* NEAR/1 (disease* or syndrome* or disorder*))
102	(farber* lipogranulomatos* or ceramidase deficien* or fibrocytic dysmucopolysaccharidos*)
103	Pelizaeus-Merzbacher Disease
104	pelizaeus-merzbacher*
105	Sulfatasesdf
106	Multiple Sulfatase Deficiency Disease
107	(sulfatase deficien* or sulphatase deficien* or mucosulfatidos*)
108	(austin* NEAR/1 (disease* or syndrome* or disorder*))
109	sulfatidosis
110	sulfatidos*
111	Sea-Blue Histiocyte Syndrome
112	sea-blue histiocyt*
113	Neuronal Ceroid-Lipofuscinoses
114	(batten* NEAR/1 (disease* or syndrome* or disorder*))
115	(neuronal ceroid lipofuscinos* or santavuori-haltia* or jansky-bielschowsky* or bielschowsky-jansky*)
116	(kuf* NEAR/1 (disease* or syndrome* or disorder*))
117	spielmeyer vogt*
118	Xanthomatosis, Cerebrotendinous
119	((cerebrotendineous or cerebrotendinous or cerebrotendious or cerebral) NEAR/3 (xanthomatos* or cholesteros*))
120	bogaert-scherer-epstein*
121	Wolman Disease

122	(wolman* NEAR/1 (disease* or syndrome* or disorder*))
123	lysosomal acid lipase deficien*
124	Mucopolysaccharidoses
125	mucopolysaccharidos*
126	(hurler* NEAR/2 (syndrome* or disease* or disorder*))
127	(hunter* NEAR/2 (syndrome* or disease* or disorder*))
128	(MPS1 or MPS2 or MPS3 or MPS4 or MPS5 or MPS6 or MPS7 or MPS-1 or MPS-2 or MPS-3 or MPS-4 or MPS-5 or MPS-6 or MPS-7 or MPSI or MPSII or MPSIII or MPSIV or MPSV or MPSVI or MPSVII or MPS-I or MPS-II or MPS-III or MPS-IV or MPS-V or MPS-VI or MPS-VII)
129	(beta glucuronidase deficien* or sly syndrome* or sly disorder* or sly disease*)
130	(maroteaux-lamy* or marotaeux-lamy* or polydystrophic dwarfism)
131	(morquio* or moriquio* or beta galactosidase deficien*)
132	(sanfilippo* or sanfillipo*)
133	Mucolipidoses
134	(mucolipidos* or pseudo-hurler* or pseudohurler*)
135	((inclusion-cell or i-cell) NEAR/1 (disease* or syndrome* or disorder*))
136	Fucosidosis
137	(fucosidos* or fucidos*)
138	"Congenital Disorders of Glycosylation"
139	((cdg or ctg) NEAR/1 (disease* or disorder* or syndrome*))
140	(carbohydrate-deficient glycoprotein NEAR/1 (disease* or disorder* or syndrome*))
141	(congenital disorder* NEAR/3 glycosylation)
142	Lesch-Nyhan Syndrome
143	juvenile gout
144	Menkes Kinky Hair Syndrome
145	menkes*
146	((copper transport or steely hair or kinky hair) NEAR/1 (disease* or syndrome* or disorder*))
147	alpha 1-Antitrypsin Deficiency
148	(antitrypsin deficien* or A1AD)
149	(AAT deficien* or alpha-1 protease deficien*)
150	bisalbumin$emi*
151	Lipodystrophy, Congenital Generalized
152	(congenital generali$ed lipodystroph* or berardinelli* or bernardnelli*)
153	Landau-Kleffner Syndrome
154	(landau-kleffner* or infantile acquired aphasia* or acquired epileptic aphasia*)
155	(aphasia* NEAR/5 convulsive)
156	Rett Syndrome
157	(rett* NEAR/1 (syndrome* or disease* or disorder*))
158	cerebroatrophic hyperammon$emi*
159	Huntington Disease
160	huntington*
161	Spinocerebellar Ataxias
162	((nyhan* or kelley-seegmiller*) NEAR/1 (syndrome* or disorder* or disease*))

163	(spinocerebellar ataxia* or ataxia* telangiectasia* or louis-bar* syndrome* or louis-bar* disease* or louis-bar* disorder* or machado-joseph* or joseph* disease* or joseph* disorder* or joseph* syndrome*)
164	Friedreich Ataxia
165	((friedreich* or friedrich*) NEAR/3 ataxia*)
166	spinocerebellar degenerat*
167	"Spinal Muscular Atrophies of Childhood"
168	(spinal muscular atroph* or werdnig hoffman*)
169	(dubowitz* or kugelberg-welander*)
170	Bulbar Palsy, Progressive
171	(fazio-londe* or faziolonde* or progressive bulbar pals*)
172	parkinson disease or parkinson disease, secondary
173	(parkinson* or hypokinetic rigid syndrome* or hypokinetic rigid disease* or hypokinetic rigid disorder* or paralysis agitan* or shaking pals*)
174	Pantothenate Kinase-Associated Neurodegeneration
175	(pantothenate kinase-associated neurodegenerat* or PKAN or hallervorden-spatz*)
176	((neurodegeneration NEAR/3 brain iron accumulation) or NBIA$)
177	Olivopontocerebellar Atrophies
178	(olivopontocerebellar atroph* or OPCA or olivopontocerebellar degenerat*)
179	(multiple system atrophy NEAR/5 cerebellar)
180	"Diffuse Cerebral Sclerosis of Schilder"
181	(alper* NEAR/1 (disease* or syndrome* or disorder*))
182	(progressive sclerosing poliodystroph* or progressive infantile poliodystroph*)
183	(diffuse cerebral sclerosis NEAR/5 schilder*)
184	Leigh Disease
185	(leigh* NEAR/1 (syndrome* or disease* or disorder*))
186	(subacute necrotizing encephalomyelopath* or subacute necrotising encephalomyelopath* or sub-acute necrotizing encephalomyelopath* or sub-acute necrotising encephalomyelopath* or SNEM)
187	(aicardi-gouti$res or aicardia-gouti$res)
188	(worster-drought* or congenital suprabulbar pares*)
189	multiple sclerosis or multiple sclerosis, chronic progressive or multiple sclerosis, relapsing-remitting
190	(multiple sclerosis or disseminated sclerosis or encephalomyelitis disseminata*)
191	(demyelinating NEAR/1 (disease* or syndrome* or disorder*))
192	Epilepsies, Myoclonic
193	myoclonic epileps*
194	((lafora* or merrf* or unverricht-lundborg* or janz*) NEAR/1 (disease* or syndrome* or disorder*))
195	lennox-gastaut*
196	(lennox* NEAR/1 (syndrome* or disease* or disorder*))
197	Spasms, Infantile
198	(west* NEAR/1 (syndrome* or disease* or disorder*))
199	Epilepsia Partialis Continua
200	(epilepsia partialis continua or kojevnikov* or epilepsia partialis continuoa or kozhevnikof*)
201	Charcot-Marie-Tooth Disease

202	(charcot-marie-tooth* or peroneal muscular atroph*)
203	(progressive neuropathic muscular atroph* or hereditary peroneal nerve dysfunction* or peroneal neuropath*)
204	"Hereditary Sensory and Motor Neuropathy"
205	(hereditary sensory NEAR/3 motor neuropath*)
206	(hereditary motor NEAR/3 sensory neuropath*)
207	Refsum Disease, Infantile
208	Peroxisomal Disorders
209	(infantile refsum or infantile phytanic acid storage)
210	Myasthenic Syndromes, Congenital
211	congenital myasth$eni*
212	Muscular Dystrophy, Duchenne
213	(duchenne muscular dystroph* or dmd)
214	Muscular Dystrophies, Limb-Girdle
215	(limb-girdle or erb* muscular dystroph*)
216	(sarcoglycanopath* or sarcoglycaopath*)
217	Osteochondrodysplasias
218	(osteochondrodysplas* or schwartz-jampel or chondrodystrophi* myotoni* or myotoni* chondrodystrophi*)
219	Myotonia Congenita
220	(congenita* myotoni* or myotoni* congenita*)
221	(thomsen* NEAR/1 (disease* or disorder* or syndrome*))
222	((recessive NEAR/3 myotoni*) or becker* myotoni*)
223	Isaacs Syndrome
224	(isaac* NEAR/1 (syndrome* or disease* or disorder*))
225	neuromyotoni*
226	Myotonic Disorders
227	(paramyotoni* congenita* or congenita* paramyotoni*)
228	(eulenburg* NEAR/1 (disease* or syndrome* or disorder*))
229	(myotoni* NEAR/1 (disease* or disorder* or syndrome*))
230	pseudomyotoni*
231	Myopathies, Structural, Congenital
232	(congenital NEAR/3 myopath*)
233	myopathycongenital
234	((nemaline or rod) NEAR/3 myopath*)
235	((central core or mini-core or minicore or multicore or multi-core) NEAR/1 (disease* or disorder* or syndrome* or myopath*))
236	fiber type disproportion
237	fibre type disproportion
238	Muscular Dystrophiescn
239	(congenital* NEAR/5 muscular dystroph*)
240	((centronuclear or myotubular) NEAR/1 myopath*)
241	Mitochondrial Myopathies
242	(mitochondrial myopath* or mitochondrial encephalomyopath* or chronic progressive external ophthalmopleg*)
243	((melas or kearns-sayre*) NEAR/1 (syndrome* or disease* or disorder*))
244	Quadriplegia and spastic*
245	(spastic quadriplegi* or spastic tetraplegi*)
246	Reye Syndrome
247	(reye* NEAR/1 (syndrome* or disease* or disorder*))

248	multiple pterygium
249	Hypertension, Pulmonary and primary*
250	((primary pulmonary or precapillary pulmonary or idiopathic pulmonary) NEAR/1 (hypertension or ht or arterial hypertension))
251	((primary bronchopulmonary or precapillary bronchopulmonary or idiopathic bronchopulmonary) NEAR/1 (hypertension or ht or arterial hypertension))
252	((primary lung or precapillary lung or idiopathic lung) NEAR/1 (hypertension or ht or arterial hypertension))
253	ipah
254	Cardiomyopathy, Dilated
255	((congestive or dilated) NEAR/1 cardiomyopath*)
256	Cardiomyopathy, Hypertrophic
257	(hypertrophic NEAR/1 cardiomyopath*)
258	Cardiomyopathiescn
259	(congenital NEAR/3 cardiomyopath*)
260	Cardiomyopathy, Restrictive
261	(restrictive cardiomyopath* or obliterative cardiomyopath* or constrictive cardiomyopath*)
262	Pulmonary Fibrosis
263	(pulmonary fibros* or lung fibros* or bronchopulmonary fibros* or fibrosing alveolit* or interstitial pneumonit*)
264	Respiratory Insufficiency
265	(respiratory NEAR/1 (failure* or insufficienc*))
266	"Cystic Adenomatoid Malformation of Lung, Congenital"
267	((cystic lung or cystic pulmonary or cystic bronchopulmonary) NEAR/1 (disease* or disorder or syndrome*))
268	(bronchogenic cyst* or bronchopulmonary foregut malformation*)
269	cystic adenomatoid malformation*
270	lobar emphysem*
271	(pulmonary sequestration* or bronchopulmonary sequestration* or lung sequestration* or extralobar sequestration* or extra-lobar sequestration* or intralobar sequestration* or intra-lobar sequestration*)
272	pulmolithias*
273	Liver Failure
274	((liver*1 or hepatic) NEAR/3 fail*)
275	Liver Cirrhosis
276	(cirrhosis NEAR/3 liver*1)
277	Hepatic Veno-Occlusive Disease
278	((veno-occlusive or venous occlusive) NEAR/1 (disease* or syndrome* or disorder*))
279	Exocrine Pancreatic Insufficiency
280	(swachman-diamond or shwachman-bodian or schwachmann-diamond or shwachmann-bodian)
281	Wegener Granulomatosis
282	wegener* granulomatos*
283	(granulomatos* NEAR/3 polyangiit*)
284	Osteolysis, Essential
285	essential osteolys*
286	((gorham* or gorham-stout* or vanishing bone or phantom bone) NEAR/1 (disease* or syndrome* or disorder))

287	((arc or arthrogryposis renal dysfunction cholestasis) NEAR/1 (disease* or syndrome* or disorder))
288	Cerebral Hemorrhagecn
289	Cerebral Hemorrhage, Traumatic
290	Cerebral Hemorrhage and Birth Injuries
291	(cerebral h$emorrhage* and (birth* NEAR/3 injur*))
292	Asphyxia Neonatorum
293	asphyxia neonatorum
294	((perinatal* or neonatal* or birth*) NEAR/3 asphyxia*)
295	Rubella Syndrome, Congenital
296	congenital rubella
297	Cytomegalovirus Infectionscn
298	(congenital NEAR/1 (cytomegalovirus* or cmv))
299	Chickenpoxcn
300	Herpes Zostercn
301	Herpesvirus 3, Human and congenital*
302	((congenital or fetal or foetal) NEAR/3 (varicella* or chicken pox* or VZV))
303	Toxoplasmosis, Congenital
304	congenital toxoplasmos*
305	Hypoxia, Brain
306	((brain* or cerebral) NEAR/3 hypoxi*)
307	Renal Insufficiencycn
308	Acute Kidney Injurycn
309	Renal Insufficiency, Chroniccn
310	Kidney Failure, Chroniccn
311	(congenital* NEAR/3 (kidney failure* or renal failure* or kidney insufficienc* or renal insufficienc*))
312	(congenital* NEAR/3 (kidney disease* or renal disease*))
313	Anencephaly
314	(anencephal* or meroanencephal* or craniorachischis*)
315	(aprosencephal* NEAR/3 open cranium)
316	Encephalocele
317	(encephalocele* or cranium bifidum)
318	Dandy-Walker Syndrome
319	dandy-walker*
320	Acrocallosal Syndrome
321	(acrocallosal or acro-callosal or acrocolossal or acro colossal)
322	Aicardi Syndrome
323	(aicardi* NEAR/1 (syndrome* or disease* or disorder*))
324	Holoprosencephaly
325	(holoprosencephal* or arhinencephal* or holosprosencephal*)
326	Hydranencephaly
327	(hydranencephal* or hydrancephal* or hydroanencephal*)
328	Lissencephaly
329	Microcephaly
330	(lissencephal* or walker-warburg* or miller-dieker* or norman-robert* or microlissencephal*)
331	((fukuyama* or muscle-eye-brain) NEAR/1 (syndrome* or disease* or disorder*))
332	"Malformations of Cortical Development"

333	(microgyria* or microgyrus or micro-gyria* or micro-gyrus)
334	(pachygyria* or pachgyria*)
335	agyria*
336	Septo-Optic Dysplasia
337	((septo-optic or septooptic) NEAR/1 dysplas*)
338	de morsier*
339	(schizencephal* or schizzencephal*)
340	Arnold-Chiari Malformation
341	chiari* malformation*
342	Truncus Arteriosus, Persistent
343	(truncus or common arterial trunk*)
344	"Transposition of Great Vessels"
345	((transposition* or dextrotransposition* or dtransposition* or levotransposition* or ltransposition*) NEAR/3 (great arter* or main arter* or aorta* or pulmonary arter* or great vessel* or main vessel*))
346	(dextro-tga or d-tga or levo-tga or l-tga)
347	(double inlet NEAR/3 ventricle*)
348	DILV
349	single ventricle*
350	Heart Defects, Congenital and Atrial Appendage
351	(isomerism NEAR/3 atrial appendage*)
352	(aspleni* or polyspleni* or poly-spleni*)
353	"Tetralogy of Fallot"
354	(tetralogy NEAR/3 fallot*)
355	Eisenmenger Complex
356	(eisenmenger* or tardive cyanos* or eisenmeyer*)
357	(pentalogy NEAR/3 fallot*)
358	Pulmonary Atresia
359	((pulmonary or bronchopulmonary or lung*) NEAR/3 atresia*)
360	Tricuspid Atresia
361	((tricuspid or tri) NEAR/3 atresia*)
362	Ebstein Anomaly
363	(ebstein* NEAR/1 (anomal* or malformation*))
364	Hypoplastic Left Heart Syndrome
365	(hypoplastic left heart NEAR/1 (syndrome* or disease* or disorder*))
366	((aortic or aorta*) NEAR/3 atresia*)
367	(mitral NEAR/3 atresia*)
368	((absence* or absent*) NEAR/3 (aorta* or aortic))
369	(aplas* NEAR/3 (aorta* or aortic))
370	Aortic Aneurysmcn
371	(((aorta* or aortic) NEAR/3 aneurys*) and congenital*)
372	(hypoplas* NEAR/3 (aorta* or aortic))
373	(convulsion* NEAR/3 (aorta* or aortic))
374	(persistent right NEAR/3 (aorta* or aortic))
375	((anomalous pulmonary venous or anamolous pulmonary venous) NEAR/1 (connection or drainage or return))
376	((absence* or absent*) NEAR/3 vena* cava*)
377	(persistent left NEAR/3 cardinal vein*)
378	Scimitar Syndrome

379	((scimitar* or pulmonary venolobar) NEAR/1 (syndrome* or disease* or disorder*))
380	(arteriovenous malformations or intracranial arteriovenous malformations) and bilateral
381	((bilateral AV or bilateral arteriovenous or bilateral arterio-venous) NEAR/3 malform*)
382	((trachea* or windpipe* or wind-pipe*) NEAR/3 atresia*)
383	Tracheal Stenosis
384	((trachea* or laryngotrachea* or glottic or subglottic or sub-glottic) NEAR/3 stenosis)
385	Bronchopulmonary Dysplasia
386	((lung* or pulmonary or bronchopulmonary) NEAR/3 (hypoplas* or dysplas*))
387	((absence* or absent*) NEAR/3 (esophag* or oesophag* or foodpipe or food-pipe* or gullet*))
388	Intestinal Atresia
389	(duoden* NEAR/3 atresia*)
390	((absence* or absent*) NEAR/3 (intestin* or gastrointestin*))
391	((intestin* or gastrointestin*) NEAR/3 atresia*)
392	((intestin* or gastrointestin*) NEAR/3 stenos*)
393	(cloaca* NEAR/3 (abnor* or malform* or anomal*))
394	(cloaca* NEAR/3 exopthalmo*)
395	Biliary Atresia
396	(biliary NEAR/3 atresia*)
397	(extrahepatic ductopen* or extra-hepatic ductopen* or progressive obliterative cholangiopath*)
398	(biliary NEAR/3 hypoplas*)
399	(alagille* NEAR/3 atresia*)
400	((absence* or absent*) NEAR/3 kidney*)
401	(potter* NEAR/1 (sequence* or syndrome* or disease* or disorder*))
402	Oligohydramnios
403	oligohydramn*
404	Multicystic Dysplastic Kidney
405	((kidney* or renal) NEAR/3 dysplas*)
406	((meckel* or meckelgruber* or gruber*) NEAR/1 (syndrome* or disease* or disorder*))
407	dysencephalia splanchnocystica*
408	(pena-shokeir* or penn-shokeir*)
409	(larsen* NEAR/1 (syndrome* or disease* or disorder*))
410	Acrocephalosyndactylia
411	acrocephalosyndactyl*
412	(pfeiffer* NEAR/1 (syndrome* or disease* or syndrome*))
413	Short Rib-Polydactyly Syndrome
414	short rib*1
415	(saldino-noonan* or majewski* or verma-naumoff* or beemer-langer*)
416	(jeune* NEAR/1 (syndrome* or disease* or disorder*))
417	asphyxiating thoracic dysplas*
418	Chondrodysplasia Punctata
419	chondrodysplasia punctata*
420	((conradi* or h$nermann* or happle*) NEAR/3 (syndrome* or disease* or disorder*))

421	Osteogenesis Imperfecta
422	osteogenesis imperfecta
423	((brittle bone or lobstein*) NEAR/1 (disease* or disorder* or syndrome*))
424	Osteochondrodysplasias
425	(spondyloepimetaphyseal or spondyloepiphyseal or spendylo metaphyseal)
426	Hernia, Umbilical
427	(omphalocele* or omphalocoele* or exomphalos)
428	(hernia* NEAR/3 umbilic*)
429	Gastroschisis
430	gastroschis*
431	Ichthyosis, Lamellar
432	(lamellar* NEAR/3 ichthyos*)
433	((harlequin* or harloquin*) NEAR/3 (ichthyos* or baby or babies or f$etus*))
434	(ichthyosis congenita* or ichthyosis fetalis or keratosis diffusa fetalis)
435	Epidermolysis Bullosa
436	epidermolysis bullosa*
437	(johanson-blizzard* or johanna-blizzard*)
438	Xeroderma Pigmentosum
439	xeroderma pigmentosum
440	Ectodermal Dysplasia
441	lacrimo-auriculo-dento-digital
442	ectodermal dysplas*
443	((ladd or eec) NEAR/1 (syndrome* or disease* or disorder*))
444	Sturge-Weber Syndrome
445	(sturge-weber or encephalotrigeminal angiomatos*)
446	Fetal Alcohol Spectrum Disorders
447	f$etal alcohol
448	Pierre Robin Syndrome
449	pierre robin*
450	Acrocephalosyndactylia
451	(acrocephalosyndact* or acrocephalopolysyndact*)
452	((apert* or crouzon* or saethre-chotzen* or noack* or carpenter* or sakati-nyhan-tisdale* or goodman*) NEAR/1 (syndrome* or disorder* or disease*))
453	Fraser Syndrome
454	(fraser* NEAR/1 (syndrome* or disease* or disorder*))
455	cryptophthalmos
456	(cyclopia*1 or cyclocephal* or synophthalmi*)
457	Goldenhar Syndrome
458	(goldenhar* or oculo-auriculo-vertebral)
459	Mobius Syndrome
460	((m$bius* or moebius*) NEAR/1 (syndrome* or disease* or disorder*))
461	Orofaciodigital Syndromes
462	(orofaciodigital or oro-facial-digital or oral-facial-digital or papillon-league* or psaume*)
463	(robin* NEAR/1 (syndrome* or disorder* or disease*))
464	(freeman-sheldon* or distal arthrogrypos* or craniocarpotarsal dysplas* or craniocarpotarsal dystroph* or canio-carpo-tarsal or windmill-vane-hand* or whistling-face)
465	De Lange Syndrome
466	((de lange* or bushy*) NEAR/1 (syndrome* or disorder* or disease*))

467	amsterdam dwarfism
468	(aarskog or faciodigitogenital or facio-digito-genital or facial digital genital or shawl scrotum or faciogenital or facio-genital)
469	Cockayne Syndrome
470	(cockayne* or neill-dingwall*)
471	(cerebro-oculo-facio-skeletal or cerebro-oculo-facial-skeletal)
472	(dubowitz* NEAR/1 (syndrome* or disease* or disorder*))
473	(robinow* or robinhow*)
474	(f$etal face or f$etal facies or f$etal faces or acral dysostos* or mesomelic dwarfism or covesdem*)
475	Silver-Russell Syndrome
476	(silver-russell* or russell-silver*)
477	(silver* NEAR/1 (syndrome* or disease* or disorder*))
478	((seckel* or harper*) NEAR/1 (syndrome* or disease* or disorder*))
479	(microcephalic primordial dwarfism or bird-headed dwarf* or virchow-seckel dwarfism)
480	Smith-Lemli-Opitz Syndrome
481	(smith-lemli-opitz* or dehydrocholesterol reductase deficien*)
482	Prader-Willi Syndrome
483	(prader-willi* or pradar-willi*)
484	Rubinstein-Taybi Syndrome
485	(rubinstein-taybi* or rubenstein-tabyii* or broad thumb-hallux)
486	((rubinstein* or rubenstein*) NEAR/2 (syndrome* or disease* or disorder*))
487	Nephritis, Hereditary
488	(alport* NEAR/1 (syndrome* or disease* or disorder*))
489	(hereditary nephritis or h$emorrhagic familial nephritis)
490	(hereditary deafness NEAR/3 nephropath*)
491	(h$ematuria NEAR/3 nephropath* NEAR/3 deafness)
492	Laurence-Moon Syndrome
493	laurence-moon*
494	Bardet-Biedl Syndrome
495	(bardet-biedl* or biedl-bardet*)
496	Zellweger Syndrome
497	zellweger*
498	((cerebrohepatorenal or cerebro-hepato-renal) NEAR/1 (syndrome* or disease* or disorder*))
499	(edward* NEAR/1 (syndrome* or disease* or disorder*))
500	"trisomy 18"
501	(patau* NEAR/1 (syndrome* or disease* or disorder*))
502	("trisomy 13" or "trisomy D")
503	"trisomy 22"
504	"trisomy 9"
505	"trisomy 10"
506	duplication syndrome*
507	(("chromosome 8" or "chr 8") NEAR/5 duplicat*)
508	Chromosome Duplication
509	X Chromosomeab
510	X Chromosome and duplicat*
511	(("chromosome x" or "chr x") and duplicat*)
512	(chromosom* abnormality NEAR/5 duplicat*)

513	"tetrasomy 5p"
514	(tetrasomy NEAR/3 mosaic*)
515	Chromosomes, Human, Pair 5 and Mosaicism
516	Tetrasomy
517	Trisomy and (chromosomes, human, pair 9 or chromosomes, human, pair 10 or chromosomes, human, pair 13 or Chromosomes, Human, Pair 18 or chromosomes, human, pair 22)
518	Chromosome Deletion and Chromosomes, Human, Pair 4
519	(delet* NEAR/5 short arm NEAR/5 "chrom* 4")
520	Wolf-Hirschhorn Syndrome
521	((wolf-hirschhorn* or wolff hirschorn* or chromosome deletion dillan* or pitt-rogers-dank* or pitt*) NEAR/3 (syndrome* or disease* or disorder*))
522	Cri-du-Chat Syndrome
523	((cri du chat* or crying cat* or 5p or lejeune*) NEAR/3 (syndrome* or disease* or disorder*))
524	Jacobsen Distal 11q Deletion Syndrome
525	((jacobsen* or 11q deletion) NEAR/5 (syndrome* or disease* or disorder*))
526	Monosomy and Chromosomes, Human, Pair 9
527	(9p minus or 9p deletion)
528	(alfi* NEAR/1 (syndrome* or disease* or disorder*))
529	(degouchy* or de gouchy* or degrouchy* or de grouchy*)
530	distal 18q
531	Hypoventilationcn
532	(ondine* curse or congenital central hypoventilation or primary alveolar hypoventilation)
533	Graft vs Host Disease and (Chronic Disease or chronic*)
534	(((graft vs host or graft versus host) NEAR/1 (disease* or syndrome* or disorder)) and chronic*)
535	or1-534
536	Terminally Ill
537	Terminal Care
538	Palliative Care
539	Hospices or Hospice Care
540	(life NEAR/2 limit*)
541	(life NEAR/2 threaten*)
542	end of life
543	eol
544	(terminal* NEAR/2 (ill or illness* or condition*1 or disease*1 or syndrome* or disorder*))
545	(terminal NEAR/2 (care* or caring))
546	palliat*
547	(care NEAR/2 dying)
548	(technology NEAR/2 dependent)
549	hospice*
550	Rare Diseases
551	Metabolic Diseases
552	(severe NEAR/2 (need or needs or illness* or disease*1 or disabilit* or impairment*1 or impediment*1 or condition*1 or disadvant* or problem*1 or syndrome*1 or disorder*1))

553	(complex NEAR/2 (need or needs or illness* or disease*1 or disabilit* or impairment*1 or impediment*1 or condition*1 or disadvant* or problem*1 or syndrome*1 or disorder*1))
554	(rare NEAR/2 (illness* or disease* or disabilit* or impairment* or impediment* or condition*1 or syndrome*1 or disorder*1))
555	(multiple NEAR/2 (need or needs or illness* or disease*1 or disabilit* or impairment*1 or impediment* or condition*1 or disadvant* or health or syndrome*1 or disorder*1))
556	(profound NEAR/2 (need or needs or illness* or disease* or disabilit* or impairment* or impediment* or condition*1 or syndrome*1 or disorder*1))
557	(intense NEAR/2 (need or needs or illness* or disease* or disabilit* or impairment* or impediment* or condition*1 or syndrome*1 or disorder*1))
558	(serious NEAR/2 (disabilit* or impairment* or impediment* or condition*1 or disadvant*))
559	or536-558
560	HIV
561	HIV Infections
562	(HIV or human immunodeficiency virus*)
563	(htlv or human t-lymphotropic virus* or human t cell lymphotropic virus*)
564	(acquired immune deficiency syndrome* or acquired immunodeficiency syndrome*)
565	(AIDS NEAR/3 (virus* or infection*))
566	(AIDS NEAR/1 (related or associated))
567	Neoplasms
568	(cancer* or carcin* or tumor* or tumour* or neoplas* or adenocarcin* or oncol* or malignan*)
569	Cystic Fibrosis
570	(cystic fibrosis or fibrocystic or fibro-cystic or mucoviscidosis or cf)
571	Cerebral Palsy
572	(cerebr* NEAR/3 pals*)
573	Muscle Spasticity
574	spasticit*
575	Quadriplegia
576	(spastic* and (quadripleg* or tetrapleg*))
577	Renal Insufficiency
578	((kidney* or renal) NEAR/3 (failure* or insufficienc*))
579	(end stage NEAR/3 (kidney or renal))
580	(("stage 5" or "stage V") NEAR/3 (kidney or renal))
581	(ESRD or ESKD or ESRF or ESKF or CRF or CKF)
582	or560-581
583	535 or 559 or 582
584	((Young NEAR/1 people*) or Youth* or Care leaver* or residential child* or Adolescen* or Young adult* or Young person* or Young men* or Young women* or Teen* or juvenile* or Younger people or Youngster* or Looked after or Child welfare or paediatric* or pediatric* or peadiatric* or Young male* or Young female* or juvenile or children* or child or childhood or (young NEAR/1 patient*) or young carer* or minors or puber* or pubescen* or ((secondary or high*) NEAR/2 (school* or education)))
585	infant
586	Child

587	586 not 585
588	Disabled children
589	Young Adult
590	Adolescent, Hospitalized
591	Adolescent, Institutionalized
592	Child, Institutionalized
593	Child, Hospitalized
594	Adolescent
595	or584,587-594
596	((transition* or transfer* or handoff or handover or hand over) and (Service* or care or clinic* or healthcare or hospital* or center* or centre* or facility or facilities or unit* or department* or institution* or agency or agencies or hospice* or provider* or program* or Coordinat* or Framework* or Managing or Managed or preparedness or Planning or Preparing or Preparation* or Plan* or Protocol* or planned or Support or Supporting or Trajectory or Trajectories or Pathway* or Process or Processes or Readiness or Partnership* or programme* or program* or training or strateg* or Failure* or Barrier* or system$))
597	((transition* or transfer* or handoff or handover or hand over) NEAR/3 (Service* or care or clinic* or healthcare or hospital* or center* or centre* or facility or facilities or unit* or department* or institution* or agency or agencies or hospice* or provider* or program* or Coordinat* or Framework* or Managing or Managed or preparedness or Planning or Preparing or Preparation* or Plan* or Protocol* or planned or Support or Supporting or Trajectory or Trajectories or Pathway* or Process or Processes or Readiness or Partnership* or programme* or program* or training or strateg* or Failure* or Barrier* or system$))
598	(continu* and (care or healthcare or Support or Supporting or Failure* or Barrier*))
599	(continu* NEAR/3 (care or healthcare or Support or Supporting or Failure* or Barrier*))
600	transition to adult care
601	continuity of patient care
602	patient handoff
603	Patient Care Planning
604	Patient transfer
605	(transition* or transfer* or handoff or handover or hand* over)
606	(601 or 603) and 605
607	or596-600,602,604,606
608	583 and 595 and 607
609	(letter or editorial or comment or news).pt.
610	animals not humans
611	608 not (609 or 610)
612	limit 611 to (english language and yr="1990 -Current")

Data extraction form

Study ID: from Covidence
Paper title:
Authors:
Date of publication:
Type of publication: e.g. peer reviewed journal, conference abstract
Publisher: e.g. journal
Extractor: DR or SJ
Date of extraction:

Item	Description	Page number
**Study setting and data**		
Country	Must be OECD for inclusion	
Setting	e.g. hospital, transition clinic	
Overall aim	e.g. looking at care through transition, looking at care variations by age	
Date of data collection		
Data sources	e.g. survey, case note review, routine medical records	
Study design	e.g. cohort, quasi-experimental, trial	
Interventions	If any – e.g. a transition programme	
**Participants**		
Number of participants		
Number pre-transition		
Number post-transition		
Diagnoses	e.g. LLC in general, heart conditions, oncology	
Age range		
Transition age	If an explicit pre-transition group identified, age or age range of transition, if not note no explicit grouping	
Genders	Male, female, both (with balance)	
Ethnic groups	Groups included, split if available	
Deprivation categories	Groups included, split if available	
**Outcomes and analyses**		
Outcome measured	e.g. number of inpatient admissions	
Groups compared, comparision period	detail on the comparison groups - e.g. range of ages in groups compared; length of time compared	
Type of measurement	e.g. mean difference, odds-ratios, risk ratios	
Subgroups	e.g. by ethnic group, diagnosis, sex – record results by subgroup below	
Statistical methods/tests	e.g. regression type, t-tests etc	
**Results**		
Measured changes in care	e.g. point estimates, 95%CI, p-values of observed changes	
Other relevant measures	Any other relevant results	

Missing data	% missing, imputation?	
**Summary**		
Study conclusions	e.g. change or no change at transition	
Limitations	Any obvious limitations/conflicts of interest	

**Newcastle Ottawa Scale**	Item	Score
Selection	Representativeness of exposed cohort	
	Selection on non-exposed cohort	
	Exposure ascertainment	
	Demonstration outcome not present at start of study	N/A
Comparability	Comparability of cohorts	
Outcome	Assessment of outcome	
	Follow-up long enough	
	Adequacy of follow-up	
**Overall**	**Total score**	

Modified Newcastle-Ottawa Scale

<u>Selection</u>

1) Representativeness of the transitioned group (in cross section) or the cohort after transition is

Score 1 if:
a) truly representative of the average child with the given condition in the community
or
b) somewhat representative of the average child with the given condition in the community
Score 0 if
c) selected group of users eg nurses, volunteers
or
d) no description of the derivation of the cohort

2) Selection of the non exposed group

Score 1 if
a) drawn from the same community as the exposed group (or if a single cohort is followed through transition)
Score 0 if
b) drawn from a different source
or
c) no description of the derivation of the non exposed cohort

3) Ascertainment of transition

Score 1 if
a) secure record (eg clinic or medical records) or age based if evidence provided that transition definitively happens at a set age
or
b) structured interview
Score 0 if
c) written self report/based on simple age cut off when not all transitioned at that age (or evidence not provided)
or
d) no description

4) Demonstration that outcome of interest was not present at start of study

Not relevant for this review. No score given.

<u>Comparability</u>

1) Comparability of cohorts on the basis of the design or analysis

Score 1 if
a) study controls for demographic differences including age (cross-sectional study) or age (cohort study)
Score an additional 1 if
b) study controls for disease severity/progression in the two groups or matching was used

Outcome

1) Assessment of outcome

Score 1 if

a) independent blind assessment

or

b) record linkage, e.g. medical/clinic/administrative records

Score 0 if

c) self report

or

d) no description

2) Was follow-up long enough for outcomes to occur

Score 1 if

a) yes (at least 1 year for both pre and post transition)

Score 0 if

b) no (less than 1 year for both pre and post transition)

3) Adequacy of follow up of cohorts

Score 1 if

a) complete follow up - all subjects accounted for

or

b) subjects lost to follow up unlikely to introduce bias - small number lost (≤10%) or description provided of those lost justifying lack of bias due to loss to follow-up

Score 0 if

c) follow up rate < 90% and no description of those lost

or

d) no statement
